# Intersectionality in quantitative research: A systematic review of its emergence and applications of theory and methods

**DOI:** 10.1016/j.ssmph.2021.100798

**Published:** 2021-04-16

**Authors:** Greta R. Bauer, Siobhan M. Churchill, Mayuri Mahendran, Chantel Walwyn, Daniel Lizotte, Alma Angelica Villa-Rueda

**Affiliations:** a Epidemiology and Biostatistics, Western University, London, ON, Canada, N6A 5C1; b Computer Science, Western University, London, ON, Canada, N6A 5C1; c Nursing School, Autonomous University of Baja California, J Street Nueva,Z.C, 21100, Mexicali, BC, Mexico

**Keywords:** Intersectionality, Epidemiology, Research methods, Systematic review, Statistics

## Abstract

**Background:**

Intersectionality is a theoretical framework rooted in the premise that human experience is jointly shaped by multiple social positions (e.g. race, gender), and cannot be adequately understood by considering social positions independently. Used widely in qualitative studies, its uptake in quantitative research has been more recent.

**Objectives:**

To characterize quantitative research applications of intersectionality from 1989 to mid-2020, to evaluate basic integration of theoretical frameworks, and to identify innovative methods that could be applied to health research.

**Methods:**

Adhering to PRISMA guidelines, we conducted a systematic review of peer-reviewed articles indexed within Scopus, Medline, ProQuest Political Science and Public Administration, and PsycINFO. Original English-language quantitative or mixed-methods research or methods papers that explicitly applied intersectionality theoretical frameworks were included. Experimental studies on perception/stereotyping and measures development or validation studies were excluded. We extracted data related to publication, study design, quantitative methods, and application of intersectionality.

**Results:**

707 articles (671 applied studies, 25 methods-only papers, 11 methods plus application) met inclusion criteria. Articles were published in journals across a range of disciplines, most commonly psychology, sociology, and medical/life sciences; 40.8% studied a health-related outcome. Results supported concerns among intersectionality scholars that core theoretical tenets are often lost or misinterpreted in quantitative research; about one in four applied articles (26.9%) failed to define intersectionality, while one in six (17.5%) included intersectional position components not reflective of social power. Quantitative methods were simplistic (most often regression with interactions, cross-classified variables, or stratification) and were often misapplied or misinterpreted. Several novel methods were identified.

**Conclusions:**

Intersectionality is frequently misunderstood when bridging theory into quantitative methodology. Further work is required to (1) ensure researchers understand key features that define quantitative intersectionality analyses, (2) improve reporting practices for intersectional analyses, and (3) develop and adapt quantitative methods.

## Highlights

• Multidisciplinary statistical methods for intersectional research were identified.
• Regression with intersectional variables was the most frequently used method.
• Decomposition analyses and decision trees were identified as promising methods.
• Intersectionality theory and methods literature was infrequently cited in studies.
• Improvements in reporting of quantitative intersectional research are needed.

## 1 Introduction

Intersectionality is a theoretical framework wherein consideration of heterogeneity across different intersections of social positions is integral to understanding health and social experiences. First published by legal scholar Kimberlé Crenshaw (1989, pp. 139–168) and developed within Black feminist theory to better explicate the situation of Black women in the U.S. (Collins, 1990; Combahee River Collective, 1977; Crenshaw, 1989, 1991), it is extendable to a wide range of intersections of ethnoracial group, gender, socioeconomic status, sexual orientation, and other social identities or positions (Bowleg, 2012; Hancock, 2007). Intersectionality posits that social positions that exist on a hierarchy of social power are not independent (Crenshaw, 1991), but rather that they shape human experience jointly. As social positions intersect at the individual level (e.g., race and gender), experiences at those intersections are influenced by larger interpersonal and structural systems of oppression such as racism and sexism (Bowleg, 2012; Collins, 1995).

While intersectionality has long been considered a primary theoretical and methodological tool for qualitative studies of identity and marginalization (Bowleg, 2008; Nash, 2008), it has emerged more recently in quantitative research across disciplines, including epidemiology and public health (Bauer, 2014; Bowleg, 2012). Concerns have been raised that intersectionality risks becoming detached from its foundations in Black feminist theory or flattened of its complexity and focus on social power dynamics and structural inequality as it travels across disciplines and nations (Carbado, 2013; Cho et al., 2013; May 2015; Salem, 2018). To integrate intersectionality, Bowleg (2012) argues that public health researchers need to understand its core tenets: multiple intersecting identities, historically oppressed and marginalized populations, and the social-structural context of health.

In adapting intersectionality for quantitative research, the works of Hancock and McCall are particularly influential. Hancock (2007) differentiates an intersectional approach from an “additive” approach that considers effects of social identities singly and assumes that effects at an intersection of identities can be understood as a sum of their parts. In contrast, intersectionality posits that experiences at an intersection are co-constituted and must be considered jointly. This distinction between additive and intersectional approaches maps onto quantitative distinctions between main effects and heterogeneity of effect. McCall (2005) further differentiates between intracategorical approaches that focus on complexity of experience within a particular social position or intersection, intercategorical approaches that focus on heterogeneity across a range of intersections, and anticategorical approaches that critique rigid social categorization itself. Most work on study design or data analysis methods has been intercategorical, generally describing inequalities across intersections. Scholars have expressed concern that repeatedly documenting inequalities, even in finer intersectional detail, can serve to reinforce ideas of inherent differences between groups rather than to point towards actionable solutions (Bauer, 2014; Bauer & Scheim, 2019b; Lofters & O'Campo, 2012).

While there are few standard practices for intersectional statistical analysis, multiple methods have been proposed, including conventional methods such as cross-tabulation analyses stratifying measures of central tendency by intersectional groups (Spierings, 2012) or regression models (Warner, 2008; Weldon, 2006). Even with common techniques such as regression, issues in mapping applications onto intersectionality frameworks are an area of robust discussion. While Else-Quest and Hyde (2016) proposed the use of multiple main effect regression models where the effects of social positions are considered independent and additive, Bowleg and Bauer (2016) argue that main effects models violate intersectionality's core premise that multiple social positions shape experience jointly, rather than independently. Regression models with interaction terms between two or more social positions allow effects of social position to vary across intersections (Bowleg & Bauer, 2016; Jackson et al., 2016; Spierings, 2012), and are commonly used. However, common log-scale models (e.g., logistic, Cox) by default produce interaction results that are in the multiplicative scale, identifying combined effects that differ from the product of the individual effects. These are less relevant to understanding both public health impact and causation than additive-scale interactions, which identify combined effects differing from the sum of the individual effects. It is unclear to what extent researchers are taking the additional steps necessary to produce additive-scale interaction results from log-scale models (Bauer, 2014; de Mutsert et al., 2009; Jackson et al., 2016), and when and how to address confounding in such analyses remains an issue (Jackson, 2017; Jackson & VanderWeele, 2019). There has also been a growing emphasis on the need for multilevel modelling to examine structural inequity by incorporating group-level variables such as state-level policies or neighbourhood-level resource indicators (Bauer, 2014; Bowleg & Bauer, 2016; Scott & Siltanen, 2017; Spierings, 2012).

Concerns have been raised that the common practice of statistical hypothesis testing—here for interactions or differences across intersections—can lead to conflation of hypothesis testing with a test of intersectionality itself. Hancock (2013) labels this the “intersectionality-as-testable-explanation” approach. Researchers sometimes appear to equate intersectionality with a “double jeopardy” hypothesis, as if it assumes greater adverse effects for marginalized positions and synergistic interactions that produce the worst outcomes at multiply marginalized intersections (Carbado, 2013). Purdie-Vaughns and Eibach (2008) note that such intersections do not necessarily have the poorest outcomes, because social identities and power relations are contextual in nature. Thus, intersectionality structures the question, rather than hypothesizing the answer. It can be considered an “analytic sensibility” (Cho et al., 2013), a theoretical framework that requires quantitative researchers to avoid assuming homogeneity across intersections both in outcomes and processes, and to structure their research and its interpretation around social power (Bauer, 2014; Bowleg, 2012).

As an intersectionality framework takes root within quantitative research, new methodologies are being applied and methodological debates advanced. This systematic review aims to document the disciplinary, geographic and temporal spread of intersectionality through quantitative research; assess whether studies met a basic threshold of engagement with intersectionality; and describe characteristics of studies applying an intersectionality framework and the methods used. A final aim was to identify emerging quantitative intersectional statistical methods, as well as areas for further development.

## 2 METHODS

### 2.1 Search strategy

In consultation with library scientists and in compliance with Preferred Reporting Items for Systematic Reviews and Meta-Analyses (PRISMA) guidelines (Moher et al., 2009), we developed a systematic review protocol. The search strategy covered disciplines where intersectionality has taken root: political science, sociology, psychology, epidemiology, and education, using Scopus (including Medline) and ProQuest Political Science and Public Administration (including PsycINFO). A multi-field search identified English-language journal articles with titles, abstracts, or keywords containing “intersectional*” and titles and keywords not containing “qualitative”; search strings and detailed inclusion and exclusion criteria are in online Appendix A. We included papers published online or in print from 1989 (when the term “intersectionality” was first published) through May 12, 2020.

Following import and de-duplication using Covidence (*Covidence Systematic Review Software*, n.d.), articles underwent joint title and abstract screening by two independent reviewers, followed by single-reviewer full-text screening. Conflicts were resolved by reviewer consensus. Articles were filed for inclusion if they appeared to be (1) original quantitative or mixed-methods research, or quantitative methods papers, and (2) explicitly applied intersectionality as a framework. Exclusion criteria included: articles not peer-reviewed, experimental studies of perceptions of others, and studies developing or validating measures. Quantitative methods suited to perception experiments or measure evaluation are relatively homogeneous and less applicable to other objectives (see B.1. and B.2. in Appendix B).

### 2.2 Data extraction strategy

We developed a data extraction table with discrete response options, capturing *article characteristics* (e.g., publication year, journal discipline), *incorporation of intersectionality* (e.g., citation of key authors, social identities/positions studied), and use of *quantitative methods* (e.g., study design, statistical methods). This table was pilot tested and refined by four independent reviewers. For methods papers without applied examples, only *article characteristics* were extracted. After several rounds of testing, *article characteristics* components were extracted by individual reviewers. For other components, each paper was initially extracted by two reviewers then finalized by consensus, with reviewer pairs alternating after each block of ten papers. After extracting about one-eighth of included papers, reviewers met to confirm concept clarity before moving towards independent review with spot checking.

### 2.3 Measurement of key variables

#### 2.3.1 Article characteristics

Journal disciplines were captured using the Ulrich's Web database ((Ulrichs Serials Analysis System USAS, 2020)), which specifies one to five disciplines per journal, and collapsed into broader categories (see Appendix C). Reviewers captured the countries of data collection and the first author's home institution. Citation counts were assessed using Google Scholar over a 12-h period on September 1, 2020.

#### 2.3.2 Incorporation of intersectionality

As the remaining analyses focus on how intersectionality is applied, we extracted measures only for papers including original data analysis (B.2. and B.3. in Appendix B). Three measures assessed engagement with intersectionality in the paper's text: 1) inclusion of a definition or explanation of intersectionality, 2) citation of any of three foundational authors (Combahee River Collective, Kimberlé Crenshaw, Patricia Hill Collins), and 3) number of quantitative intersectionality methods papers cited. The latter was based on a bibliography of 45 papers (B.1. and B.2. in Appendix B): 36 methods papers identified in this review, seven highly cited methods papers that apply across qualitative and quantitative studies (Bowleg, 2008, 2012; Cole, 2009; Hancock, 2007; McCall, 2005; Nash, 2008; Shields, 2008), and two additional commentaries responding to included methods papers (Del Toro & Yoshikawa, 2016; Schwartz, 2017).

Intersecting social identities/positions authors purported to examine were identified, and incongruent measures reassigned (e.g., claimed to examine race but measured immigration status). Each paper was then classified based on whether all position variables reflected categories tied to social power, which sometimes depended on authors’ justification. As a central tenet of intersectionality is embodiment and co-constitution of social identities/positions at intersections, this must be reflected in methods allowing outcomes or effects to be estimated independently for all intersections under study. For example, a regression with interaction terms for all the intersection-related variables would allow for such estimation. Comparatively, a main effects regression would only estimate effects for intersections as the sum of their social identity parts. Studies met this criterion if at least one method used allowed for such independent estimation. Reviewers also recorded whether results were reported for all intersections.

#### 2.3.3 Quantitative methods

Reviewers recorded quantitative only versus mixed methods, study design type, study sample information, and sample size (largest where multiple samples used). Reviewers captured up to three statistical methods specified by authors to be intersectional (explicitly or based on a theoretical model), regardless of whether we would consider them intersectional. Because it is conventional to conduct descriptive bivariate analyses in conjunction with more complex statistical methods, the descriptive analysis category was limited to articles using only uni/bivariate analysis. Where a paper applied multiple regression with intersectional interaction terms, the type of regression (e.g., linear, logistic) and the scale on which interactions were reported (additive, multiplicative) were extracted.

### 2.4 Data analysis

Frequencies were estimated for each extracted variable, with a geographic heat map used to display countries of first author affiliation and of data collection.

### 2.5 Data quality statement

This analysis serves as a descriptive review of an emerging field, with assessment of application of theory and methods, rather than as an outcome assessment; thus, data extraction invited a higher degree of subjectivity than expected. For example, social positions or methods purported to be intersectional were not always explicitly stated. In all cases, reviewers attempted to extract data to be as reflective as possible of the authors’ stated objectives. Measures taken to ensure data accuracy included the use of a set of common definitions for all variables, as well as random and targeted post-data extraction quality checks. As with all quantitative research, we acknowledge that some nuance may have been lost to the discrete nature of the data extraction process. We attempted to mitigate this limitation wherever possible by using illustrative examples.

## 3 RESULTS

### 3.1 Study characteristics and theoretical engagement

**Fig. 1:**
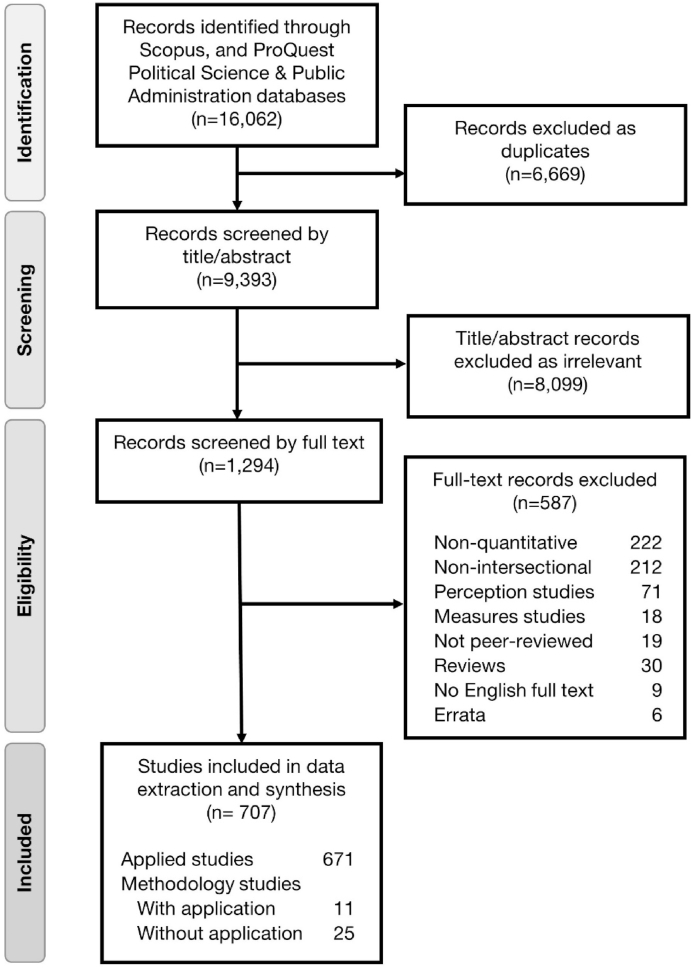
Flow diagram.

**Fig. 2:**
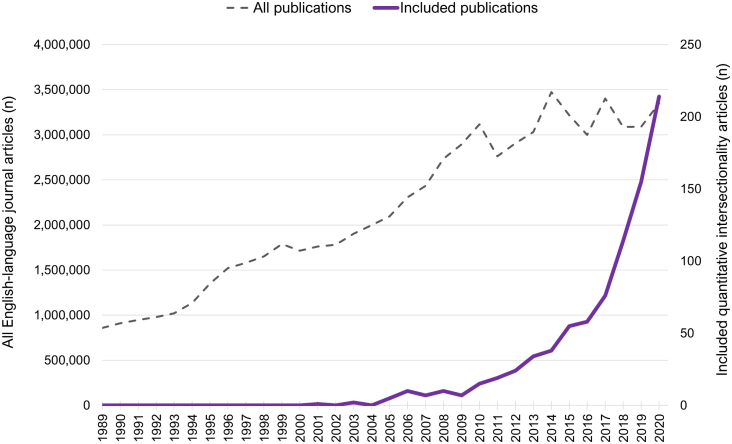
Time trend of quantitative intersectionality publications in comparison with all peer-reviewed publications 2020 numbers are rescaled full-year estimates from partial-year data. N peer-reviewed publications (all, including 2020 estimates) = 69.8 million. N peer-reviewed publications (included only) = 707.

**Fig. 3:**
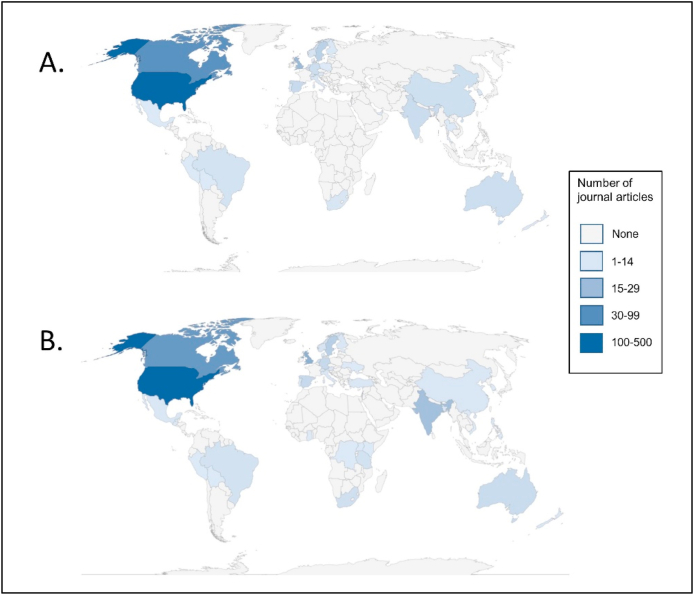
Geographical heat map of quantitative intersectionality articles by A. country of first author (n = 707), and B. country of data collection (n = 681).

Search results and exclusions are shown in Fig. 1. The final sample consisted of 707 articles: 671 applied-only studies and 36 methods papers, 11 of which also included applications (one used only published and hypothetical data, and was excluded from application subgroup analyses). Article characteristics are shown in Table 1 and Fig. 2, Fig. 3. While the term “intersectionality” was first published in 1989, the first quantitative intersectionality paper appeared in 2001, and 94.1% of papers have been published since 2010. Most common journal disciplines included psychology, sociology, medical and life sciences, other social sciences, and gender and sexuality; of applied papers, 40.8% studied a health-related outcome and 21.9% focused on children or youth. Among methods studies, journals in gender and sexuality and in medical and life sciences were most prominent. Of first authors, 73.8% were based at U.S. institutions. Geographic distribution of authors and data differed, with some countries (e.g., in Africa) represented in data but not first authorships.

**Table 1:** Characteristics of included articles (n = 707).

	Total (n = 707)		Applied papers (n = 671)		Methods papers (n = 36)	
	n	%	n	%	n	%
Publication Decade						
1989 a-1999	0	0	0	0	0	0
2000–2009	42	5.1	38	5.7	4	11.1
2010–2020 (through May 12)	665	94.1	633	94.3	32	88.9
Journal Discipline b						
Psychology	170	24.0	159	23.7	11	30.6
Sociology	163	23.1	162	24.1	1	2.8
Medical and Life Science	150	21.2	133	19.8	17	47.2
Other Social Sciences	118	16.7	99	14.8	19	52.8
Gender and Sexuality	105	14.9	96	14.3	9	25.0
Population/Public Health and Safety	81	11.5	80	11.9	1	2.8
Political Science	56	7.9	55	8.2	5	2.8
Law & Criminology	51	7.2	51	7.6	0	0.0
Education	50	7.1	49	7.3	1	2.8
Ethnic Studies	47	6.6	47	7.0	0	0.0
Business and Economics	29	4.1	29	4.3	0	0.0
Children and Youth	26	3.7	26	3.9	0	0.0
Physical, Earth & Space Sciences	22	3.1	21	3.1	1	2.8
Other Sciences	17	2.4	15	2.2	2	5.6
Philosophy and Religion	8	1.1	7	1.0	1	2.8
Public Policy	7	1.0	6	0.9	1	2.8
Disability	3	0.4	3	0.4	0	0.0
Sports and Recreation	2	0.3	2	0.3	0	0.0
History	1	0.1	1	0.1	0	0.0
Statistics	1	0.1	1	0.1	0	0.0
Humanities	1	0.1	1	0.1	0	0.0
Country of first author						
United States	522	73.8	500	74.5	22	61.1
Canada	50	7.1	44	6.6	6	16.7
United Kingdom	28	4.0	26	3.9	2	5.6
Sweden	15	2.1	12	1.8	3	8.3
Spain	10	1.4	9	1.3	1	2.8
India	9	1.3	8	1.2	1	2.8
Australia	8	1.1	7	1.0	1	2.8
Germany	8	1.1	8	1.2	0	0.0
Other c	57	8.1	57	8.5	0	0.0
Citation Count						
<10	351	49.6	340	50.7	11	30.6
10-49	245	34.7	232	34.6	13	36.1
50-99	70	9.9	65	9.7	5	13.9
100-199	29	4.1	26	3.9	3	8.3
200-499	10	1.4	8	1.2	2	5.6
≥500	2	0.3	0	0.0	2	5.6

a The term “intersectionality” was published by Kimberlé Crenshaw in 1989.

b Multiple disciplines per journal; proportions do not sum to 100%.

c Countries with <1% of total papers are grouped into “other” and can be seen in Fig. 3.

**Fig. 4:**
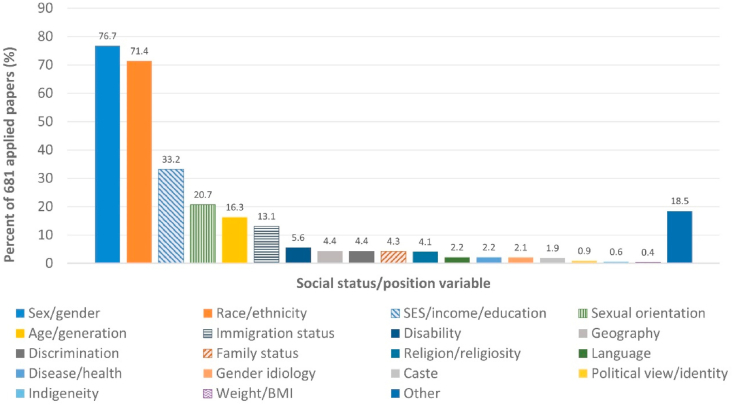
Social positions used in quantitative intersectionality analyses (n = 681 papers).

Table 2 and Fig. 4 display indicators of theoretical engagement in the 681 papers with original data applications. Results suggest a limited understanding and application of intersectionality. Only 73.1% of applied studies provided a definition or explanation of intersectionality. While 68.0% cited at least one of three foundational authors, nearly half (47.0%) did not cite any of the 45 methods papers we queried, and 25.0% cited only one, indicating a low level of engagement with methods literature. We note that some papers pre-dated the publication of many of these methods papers. However, only three predated all of them, and the mean number of methods citations per paper has remained near one over the past decade.

**Table 2:** Application of theory in quantitative analyses (n = 681).

Characteristic	n	%
Intersectionality defined	498	73.1
Cited foundational author(s)	463	68.0
Engagement with methodology papers^a^		
0 cited	320	47.0
1 cited	170	25.0
2-4 cited	165	24.2
5+ cited	26	3.8
All positions based in social power	562	82.5
Number of social positions analyzed in intersections		
1	10	1.5
2	302	44.3
3	197	28.9
4	74	10.9
5+ (maximum = 16)	98	14.4
Methods allow outcomes/effects to vary for all intersections^b^^,^^c^	502	81.4
Paper presents results for all intersections of interest^b^^,^^d^	356	57.7

a List of 45 methodology papers included in online Appendix B (B.1. and B.2.).

b Of n = 617 papers with clear intersectional groups for which we would expect outcomes/effects to be estimated; excluded were 64 papers that assessed one intersection, focused on process variables (e.g, continuous measures of discrimination), or both.

c At least one method, if multiple methods used.

d Including those grouped together in decision tree leaves.

In selecting social positions of interest, most studies included a sex/gender (76.7%) and/or a race/ethnicity (71.4%) perspective (Fig. 4). Other prominent positions included socioeconomic status, including income or education (33.2%), and sexual orientation (20.7%). While most papers used self-reported social identities/positions, some used measures of discrimination or other factors related to social positions, such as measures of racial centrality in a sample that was entirely African American (Oney et al., 2011) or measures of internalized gender norms in a sample that was entirely male (Ojeda et al., 2016) This served to assess the underlying processes driving outcomes within intersections. For 82.5% of papers, all social identities/positions were clearly grounded in concepts of social power, and authors often presented a rationale for why less common social positions (e.g., disease statuses, body weight) may reflect social power based on stigmatization. Ten studies (1.5%) included only one social position; eight were intracategorical analyses within a selected sample (e.g., analysis of race within a sample of women), one measured the same position for two interacting parties (Gilliard-Matthews, 2017), and one conceptualized an intersection using a school-level contextual variable (Valiente & Rambla, 2009). Most commonly, two or three social identities/positions were used (73.2% of applied studies), producing a limited number of intersections that would make most data analysis methods feasible. Of 617 papers with clear intersectional categories for which to estimate outcomes or effects, 81.4% used methods that would allow study outcomes or effects to be independently estimated across intersections, but only 57.7% reported results for all intersections.

### 3.2 Study design and statistical analysis methods

Of 681 articles with quantitative applications, 91.9% were quantitative only and 8.1% mixed methods (Table 3). Study designs were primarily cross-sectional (81.6%) and prospective cohort studies (12.8%). Data in 29.7% of articles were based on complex multi-stage samples, such as national population studies, and in 8.7% were based on census or population registry data. Sample size was very small (n < 100) for 4.0% of articles, but exceeded 10,000 for 30.4%.

**Table 3:** Methods used in quantitative analysis (n = 681).

Characteristic	n	%
Study type		
Quantitative	626	91.9
Mixed-methods	55	8.1
Study design		
Cross-sectional study	556	81.6
Prospective cohort study	87	12.8
Time series	21	3.1
Retrospective cohort study	7	1.0
Randomized controlled trial	4	0.6
Delayed treatment trial	1	0.1
Meta-analysis	1	0.1
Design unspecified	4	0.6
Complex multi-stage sample^a^	202	29.7
Data from census or population records (e.g., birth records)	59	8.7
Sample size^b^		
<100	26	4.0
100–499	145	22.2
500–999	71	10.9
1000–4999	159	24.3
5000–9999	54	8.3
10,000–49,999	118	18.0
50,000–99,999	18	2.8
100,000+	63	9.6
Statistical methods used^c^		
Regression with interactions	196	28.8
Regression using intersection variables^d^	202	29.7
Regression using main effects	118	17.3
Descriptive analysis only	92	13.5
Multilevel modelling^e^	55	8.1
Structural equation modelling	31	4.6
MANOVA	17	2.5
MAIHDA	10	1.5
Decomposition	9	1.3
Latent class/profile analysis	10	1.5
Cluster analysis	5	0.7
Decision tree	7	1.0
Other	34	5.0
Regression model type^f^		
Logistic	257	50.0
Linear	164	31.9
Poisson	16	3.1
Negative binomial	13	2.5
Proportional hazards	11	2.1
Log linear	6	1.2
Log binomial	4	0.8
Negative log-log	1	0.2
Unspecified type	39	7.6
Scale used for reporting regression interactions^g^		
Additive-scale interaction from linear model	73	37.2
Additive-scale interaction only from log-scale model	3	2.4
Both scales from log-scale model	6	3.1
Multiplicative-scale interaction only from log-scale model	112	57.1
Unspecified	2	1.0

a 3.8% were unspecified.

b Of n = 654 papers with sample size reported; largest sample size, where multiple data sets or analyses included; range was from 10 to 714.3 million (using US census data across four decades).

c Some papers included more than one method; will sum to >100%. Exception is descriptive analysis, which is typically included in conjunction with all other methods; here it is limited to papers with descriptive-only analysis (e.g., frequencies, cross-tabulations).

d Intersections coded and used as independent variables, or as stratification variables.

e Multilevel models had levels above the individual (e.g., schools, neighbourhoods, states) and/or below the individual (e.g., repeated measures).

f Among n = 514 papers with regression analysis; may use more than one type, so will sum to >100%.

g Among n = 196 papers using regression with interactions; n = 73 studies used linear regression and n = 123 used log-scale (multiplicative-scale) models (e.g., logistic, Poisson). Papers may include more than one interaction type for same or different regressions; will sum to >100%.

Data analysis methods stated to be intersectional were frequently descriptive, and statistically simple; descriptive classifications here included contingency table analyses and other tests of difference between descriptive estimates. A moderate proportion of papers (13.5%) used only descriptive statistics (e.g. measures of central tendencies or bivariate statistics) to fulfill their quantitative intersectional objectives, while 17.3% applied main-effects regression, which does not allow for effects to vary across intersections. The most common methods were those applying regression in ways that allow for heterogeneity across intersections (e.g., regression with interactions, stratification, or cross-classified independent variables representing intersections). These three approaches were also carried throughout most methods beyond standard regression, including MANOVA, multilevel modelling, and growth curve analysis.

Multilevel models of multilevel data were used by 8.1% of studies, and multilevel models of individual-level data by 1.5%; the latter are sometimes termed multilevel analysis of individual heterogeneity and discriminatory accuracy (MAIHDA) models. Of 55 papers using multilevel data, 15 (27.3%) included higher-level social context variables (e.g. percentage foreign-born within a population (Berg, 2010)), and 19 (34.5%) used lower-level repeated measures, including for growth curve analysis. Others used multilevel models only to account for study design (e.g., clustering).

Among 514 studies that used at least one type of single-level regression analysis, half (50.0%) used logistic regression, with linear regression being the other most common type (31.9%). Notably, most studies that used regression used multiplicative-scale methods such as logistic, Poisson, Cox, or negative binomial regressions. Among the 196 papers using regression with interaction, 73 (37.2%) used linear models that would produce additive-scale interactions. Of the 123 using interaction terms in log-scale models, only 9 took the extra steps required to report additive-scale interactions, alone or in addition to multiplicative-scale interactions. Most (57.1%) regression with interaction analyses reported interactions only in the multiplicative scale, which is less relevant for both causation and public health impact. Decision-tree methods, which are scale-free, were used much less commonly.

### 3.3 Limitations in data extraction

The final variables in this review excluded several we originally intended to extract. Reviewers were unable to reach sufficient agreement on how typologies applied to quantitative research. These included whether studies mapped onto McCall's (2005) intercategorical, intracategorical, or anticategorical approaches, and Bauer and Scheim's (2019b) descriptive or analytic approaches. Poor reporting also hampered identification of whether studies incorrectly advocated for main-effects as an intersectional approach, represented a multiple-marginalization approach, or framed intersectionality as a testable hypothesis (Hancock, 2013). Other intended variables were altered: multidimensional social positions had to be grouped together (e.g., sex/gender, race/ethnicity), and we were unable to grade the quality of definitions of intersectionality.

## 4 Discussion

Common in qualitative research, intersectionality has only recently been incorporated into quantitative research across disciplines. We note that while intersectionality scholars have advocated for mixed-methods studies (Agénor, 2020; Bowleg & Bauer, 2016), they constituted a small proportion of studies and a potential missed opportunity. While the emergence of intersectionality within U.S. Black feminism is reflected in the high frequencies of U.S. data collection and first authors’ institutions, and in the primacy of race/ethnicity and sex/gender as analytic categories, the framework has been applied well beyond both these geographic and categorical boundaries. Given that we reviewed only English-language publications, there is likely even greater diversity of geographic locations and applications than was captured. In order to graft quantitative intersectionality more tightly to its theoretical roots, we highlight three broad areas for improvement in application and reporting: theoretical conceptualization and approach, methods for sampling and measurement, and statistical analysis.

### 4.1 Theoretical conceptualization and approach

Engagement with intersectionality's core tenets was often superficial, as evidenced by a lack of any definition (26.9% of papers), non-citation of foundational authors (32.0%) or of any intersectionality methods papers (47.0%), and use of ‘intersectional’ categories not explicitly tied to social power (17.5%). These represent basic levels of incorporation of intersectionality. In reviewing papers, we observed weaknesses in deep engagement with ideas of power and in explicitly linking theory with methods and interpretation. These oversights may be a product of concept trendiness (Davis, 2008), in that researchers may latch onto a concept without a foundational understanding of its central tenets (Bowleg, 2012). In order for intersectionality to be clearly understood within quantitative studies, authors must explicitly identify the intersectional positions of interest and how they reflect social power, as well as specify their intersectional approaches, assumptions, and interpretations, making the match between theory and methods clear.

Extraction difficulties our team experienced raised questions regarding whether McCall's (2005) categorization of intercategorical, intracategorical, and anticategorical approaches to intersectionality bridges well from qualitative to quantitative methods. Studies based on intersectional categories within full populations (Agénor et al., 2019; Axelsson Fisk et al., 2018) were easily identifiable as intercategorical, and those exploring experiences within a single intersection, for example cumulative trauma in single mothers on income assistance (Samuels-Dennis et al., 2010, p.), or attitudes toward white privilege among white Christian students (Todd et al., 2014), were clearly intracategorical. Others fell into a grey zone, and few authors specified their approach. Studies examining intersectional categories within selected samples could be construed as inter- or intracategorical. When unspecified, it was also impossible to distinguish whether a main-effects analysis of multiple social positions (e.g. race, sexual orientation) among a selected sample (e.g. women) represented a failed attempt at intercategorical intersectionality, or an intracategorical approach. McCall (2005) acknowledged her typology is not exhaustive and that some research may fall within multiple approaches or none at all.

Just a single method, discriminatory accuracy (DA), was identified as potentially anticategorical; Wemrell et al. (2017a; 2017b) point out that it “direct [s] critique toward categorization itself” through measurement of intragroup heterogeneity, but is not *a priori* anticategorical. It is unclear how findings of within-group heterogeneity on a health measure critique the category itself, though they provide a useful corrective to an overfocus on point estimates. McCall identifies the substantive goal of anticategorical approaches as “deconstructing the normative assumptions of these categories” which serve to reproduce systematic inequalities (McCall, 2005). Under this conceptualization, anticategorical intersectionality appears incompatible with quantitative research, which is deeply dependent on categorization. DA may thus be better described as a useful approach to studying outcome heterogeneity within and across intersections.

We also originally intended to capture whether studies were conceptualized as descriptive intersectionality approaches focused on estimates for intersections and differences across them, or as analytic intersectionality approaches aiming to address causal processes that produce intersectional inequalities (Bauer & Scheim, 2019b). These could not feasibly be distinguished, as the central research question was sometimes neither explicitly stated not implicitly ascertainable from the analysis plan. For example, some studies measured inequitable processes (e.g., racism) in place of social positions, treating them as measures of categorization rather than a causal process. Moreover, some papers used a blockwise or mediation approach that, while not meeting Bauer and Scheim's requirement to allow processes to vary across intersections, also represented an explanatory analysis.

Finally, the mis-theorization of intersectionality as a testable hypothesis (Hancock, 2013) rather than an analytic framework was dropped early in the extraction process, as authors were often unclear on how theory informed their analysis. Reviewers had difficulty distinguishing publications with this conceptual error from those using hypothesis testing without this conceptual assumption. While not quantified in our review, this remains a central misapplication often commented on by intersectionality scholars (e.g., del Río-González et al., 2021).

Another theoretical misapplication noted but not quantified regarded differentiating between what Hancock (2007) called the multiple versus intersectional approaches, or approaches that treated effects of social identities/positions as additive versus intersectional. This was a common misapplication, even in methods papers (Bowleg & Bauer, 2016; Else-Quest & Hyde, 2016). Intercategorical approaches involving use of main effects regression, creation of a metric for number of marginalized groups to which participants belong, or hypothesized unidirectional stepwise effects from each additional marginalized identity, generally do not allow for co-constitution of experience within intersections. These approaches clearly map onto Hancock's multiple (non-intersectional) approach, and in the absence of a clear rationale, cannot be considered intersectional. Mereish and Bradford (2014) acknowledged that while intersectional perspectives do not justify this approach, existing empirical evidence could provide grounds for it.

Ultimately, improvement in theoretical conceptualization and approaches within quantitative research will depend on researchers being explicit regarding their aims, hypotheses, and application of intersectionality within their research approaches. The process of making these explicit may also drive a deeper engagement with ideas in foundational and methods literature. To formalize these and other potential recommendations for reporting, the creation of reporting guidelines for intersectional research may be helpful, in consultation with intersectionality theorists and methodologists.

### 4.2 Methods for sampling and measurement

The vast majority of applied studies (81.6%) were based on cross-sectional samples, and most sample sizes exceeded 1000. Studies often drew upon large national or regional data sets with complex probability samples. Such data sets should be optimized for intersectional analysis, for example with oversampling of any groups that remain too small for precise estimates. The preponderance of cross-sectional data suggests poor fit between available data resources and analyses of causal questions, which are better suited to longitudinal data.

Validity of measures for social identity/position variables was rarely discussed, though some studies explicitly used proxy measures for those of stated interest (e.g., benefits recipients for people with disabilities (Ballo, 2020)). While most studies included a measure of race or ethnicity and sex or gender, the multidimensionality of these constructs was often unacknowledged. For example, race/ethnicity multidimensionality may include racial identity, the race others perceive one as, legal racial/ethnic status, race centrality, tribal membership, ethnic ancestry, country of birth, caste, language and/or skin shade. Within quantitative disciplines such as epidemiology, the multidimensionality of racial categories (Jones, 2001; Muntaner et al., 1996) and sex/gender categories (Krieger, 2003) has long been recognized, though measurement validity and proxy performance are rarely evaluated. Intersectionality's questioning of the boundaries and sociohistorical construction of categories suggests that questioning, or at least acknowledging the limits of categorization is fundamental. Hancock (2007) offered fuzzy-set theory as a potential solution to these limitations, wherein categories are coded with fuzzy boundaries taking a range of values from fully in-group (1) to fully out-group (0); however, no applications of fuzzy-set theory were identified. Further research should continue to push the boundaries of quantitative methods regarding the limitations of social categorization.

While we explicitly excluded studies of measure development and validation, we note that statistical analysis is interdependent with measurement. While intersectional statistical methods papers generally focus on intercategorical complexity, most intersectional measures focus on intracategorical complexity (e.g., discrimination as experienced among racialized sexual minority persons) (Bauer & Scheim, 2019a). Measures for process-type constructs that only exist, or have differential meaning, for specific intersections place constraints on the types of statistical analyses that can be conducted. Bright et al. (2016) have labelled as “switch intersectionality” the concept that certain causal pathways may exist only for certain intersections; the implications for measurement and analysis require additional development.

### 4.3 Methods for statistical analysis

The majority of quantitative intersectionality studies used basic statistical analysis methods, such as descriptive estimates (with or without confidence intervals or statistical tests), main effects regressions, or regression with interaction. Researchers often did not clearly distinguish between regression analyses of intersectional inequalities versus causal effects (Bauer & Scheim, 2019b), or provide rationales behind multivariable analyses. For example, though descriptive analyses should not be adjusted for potential confounders, studies designed for causal understanding must be; yet, covariates were often included without clear rationale as to the role they played.

Several promising methods for estimating outcomes across large numbers of intersections (>100) were published in recent years. These included decision tree methods such as classification and regression trees (Cairney et al., 2014) or chi-square automatic interaction detection analysis (Shaw et al., 2012). These methods allow data-driven exploration of heterogeneity within populations across social identities/positions, though at risk of arbitrary data splits that may not be replicated across data sets (Cairney et al., 2014). Evans et al. (2018) introduced MAIHDA models, a multilevel regression application for large numbers of intersections using individual-level data (Merlo, 2018). This method partitions variance within and between intersectional clusters, where the significant residual values are interpreted as the additional intersectional effect (Axelsson Fisk et al., 2018; Evans et al., 2018). While a simulation study has questioned the intersectional interpretation of these residuals and the fixed effects (Lizotte et al., 2020), this method holds promise as a statistically efficient method for predicting outcomes across large numbers of intersections (Bell et al., 2019; Mahendran, Lizotte, Zhu, & Bauer, n.d.).

Additional newer methods analyse a smaller number of intersections by applying counterfactual causal theory to decompose and explain either inequalities or mediated causal effects. Decomposition of inequalities into individual and joint social identities/positions has been proposed by Jackson (2017; 2016). Bauer and Scheim (2019b) propose an intersectional mediation analysis based on VanderWeele's (2013) three-way decomposition, applied to assessing potential drivers of intersectional inequalities. This method allows for the effect and the level of the mediator to differ between intersectional groups, providing intersection-specific estimates for the effects of modifiable mediators. While structural equations models (SEM) could be similarly used in ways that reflect intersectional heterogeneity encoded in interactions, most SEM applications investigated sequential pathways within a given intersection. Intersectional methods for the study of causal processes need additional attention, including structure of analysis, control of confounding, and how to best inform interventions (Bauer & Scheim, 2019a; Jackson & VanderWeele, 2019).

Discriminatory accuracy analysis emerged as a potential tool to guide intervention planning in public health (Merlo et al., 2017; Wemrell et al., 2017a, 2017b). The substantive goal of DA analysis is to evaluate intragroup heterogeneity, and is an important correction to the “tyranny of the averages” (Merlo et al., 2017). Outcomes of a DA analysis might include implementing an intervention targeted at certain intersectional groups if the given categorization demonstrates high DA, while otherwise opting for either individualized or universal interventions (or alternate categorizations) if it is low, in order to avoid ineffective interventions that may also stigmatize particular groups.

Clustering methods such as latent class or latent profile analysis (LCA or LPA) were most often applied to create process-related classes of experiences of discrimination or violence (e.g. (Byrd & Carter Andrews, 2016)). The resulting process class variables have the potential to be used in different types of analysis (e.g., effect-measure modification, mediation, SEM) to better understand their roles in impacting those at different intersections. Other applications included clustering on social positions, though this obscures individual intersections in favour of creating “intersectional classes” containing varying frequencies of marginalized groups.

We identified some key areas for future focus. Firstly, additional assessment of the quality of methods and their application is needed, particularly regarding their match with intersectionality. Secondly, a clear evaluation of how data analysis methods perform within an intersectionality framework and under various data scenarios (e.g., sample sizes, number of intersections) is also needed. Findings from traditional approaches such as regression with interaction terms and novel techniques such as machine learning may not be exchangeable, and validity and precision of estimates are important to assess. Thirdly, methods for analytic intersectionality can be further expanded to be more applicable to common scenarios such as multiple mediators. Finally, intersectionality research can be better structured to support decision-making in evidence-based policies and interventions. This includes an analytic focus on causal processes, intervenable factors, interventions themselves, and heterogeneity within intersections.

## 5 Conclusion

Although intersectionality has been applied predominantly in qualitative research, its use has risen considerably in quantitative and mixed-methods studies, including the medical and life sciences. Meaningful application, however, requires clear understanding and engagement with the core tenets. This review identified significant room for improvement in explicitly connecting research methods and reporting to intersectionality frameworks, and provides some initial guidance for improvement in reporting. A limitation of this study was that we were unable to assess the quality or correctness of papers’ intersectionality definitions, adherence to central tenets, methods applications, and intersectional interpretation. Measurements and quantitative analyses consistent with the central theoretical tenets of intersectionality can contribute to the analysis of health problems in micro and macro socio-structural ways using large-sample data, with the potential to impact the development and implementation of public policy, and ultimately health equity.

## FUNDING

This work was supported by the 10.13039/501100000024Canadian Institutes of Health Research, 10.13039/501100000029Institute of Gender and Health, through a project grant (MOP-130489) and a Sex and Gender Science Chair (GSB-171372). The funders had no role in the study design, analysis or preparation of results.

## Ethics statement

Analysis for this systematic review is based on published journal articles, and does not constitute human subjects research. No ethics board approval was required.

## CRediT authorship contribution statement

**Greta R. Bauer:** Conceptualization, Methodology, Formal analysis, Resources, Writing – review & editing, Visualization, Funding acquisition. **Siobhan M. Churchill:** Conceptualization, Methodology, Validation, Investigation, Data curation, Writing – review & editing, Project administration. **Mayuri Mahendran:** Methodology, Validation, Formal analysis, Investigation, Data curation, Writing – review & editing, Project administration. **Chantel Walwyn:** Methodology, Validation, Investigation, Data curation, Writing – review & editing. **Daniel Lizotte:** Methodology, Writing – review & editing. **Alma Angelica Villa-Rueda:** Investigation, Methodology, Writing – review & editing.

## Declaration of competing interest

The authors declare that they have no conflicts of interest.
