# Supplementary material for: Intersectionality in quantitative research: A systematic review of its emergence and applications of theory and methods
Source: SSM Popul Health. 2021 Apr 16;14:100798. doi: 10.1016/j.ssmph.2021.100798 (PMC8095182; doi:10.1016/j.ssmph.2021.100798)
Supplement: Multimedia component 1 [file mmc1.pdf]

Information provided by authors to accompany article:

Bauer GR, Churchill SM, Mahendran M, Walwyn C, Lizotte D, Villa-Rueda AA. Intersectionality in quantitative research: A systematic review of its emergence and application of theory and methods. *SSM – Population Health* 2021.
https://doi-org.proxy1.lib.uwo.ca/10.1016/j.ssmph.2021.100798

Contents

APPENDIX A. SUPPLEMENTAL INFORMATION ON SEARCH AND SCREENING ... 1
Table A.1. Search strings ... 1
Table A.2. Decision rules for full-text screening ... 2
APPENDIX B. BIBLIOGRAPHIES ... 3
B.1. Bibliography: Excluded studies—Experimental perception/stereotyping studies that apply intersectionality ... 3
B.2. Bibliography: Excluded studies—Development or validation of intersectional measures ... 9
B.3. Bibliography: Original application papers for quantitative intersectionality ... 11
B.4. Bibliography: Methods papers for quantitative intersectionality that included an original data analysis application ... 62
B.5. Bibliography: Methods-only papers for quantitative intersectionality ... 63
APPENDIX C. JOURNAL DISCIPLINE CATEGORIES ... 67

APPENDIX A. SUPPLEMENTAL INFORMATION ON SEARCH AND SCREENING

Table A.1. Search strings

Database	Results	Search String + Limits
Scopus (including Medline, Embase, more...)	N=6,016	(TITLE-ABS-KEY (intersectional*) AND NOT KEY (qualitativ*) AND NOT TITLE (qualitativ*)) AND PUBYEAR > 1988 AND (LIMIT-TO (LANGUAGE,"English ")) AND (LIMIT-TO (DOCTYPE,"ar") OR LIMIT-TO (DOCTYPE,"ip"))
ProQuest Political Science (including PsycINFO)	N=10,046	if,ti,ab(intersectional*) NOT if,ti(qualitativ*)<br>Date: After 1988<br>Record type: Dissertation, Dissertation Abstract, Journal Article<br>Language: English

Table A.2. Decision rules for full-text screening

Criteria	Included	Excluded
(1) Original quantitative research or quantitative methods papers	- Quantitative studies<br>- Mixed-methods studies<br>- Original methodological papers (applied or not) which provided direction for quantitative intersectional research	- Content or discourse analyses, both qualitative and quantitative<br>- Mixed-methods studies where intersectionality was only applied in the qualitative portion<br>- Studies using the term intersectionality only as it applies to quantitative set theory (e.g. soft or fuzzy set theory)
(2) Explicitly applied intersectionality theory to estimate outcomes or effects at particular intersections of a population	- Studies which explicitly applied an intersectional theoretical framework	- Studies which used intersectionality as a discussion point to interpret results rather than an *a priori* theoretical framework<br>- Intersectional perception/stereotype studies involving subjects for which perceptions of certain targets (often photographs or descriptions of people at different intersectional positions) were observed<br>- Studies developing a measure to capture an intersectional construct (e.g., gendered-racial socialization)<br>- Studies of intersectionality only as a subject rather than intersectionality of participants (e.g., an opinion survey to gauge intersectional consciousness)

APPENDIX B. BIBLIOGRAPHIES

Information provided by authors to accompany article <INSERT FINAL CITATION HERE>

B.1. Bibliography: Excluded studies—Experimental perception/stereotyping studies that apply intersectionality

1. Atkinson, J. L., & Sloan, R. G. (2017). Exploring the impact of age, race, and stereotypes on perceptions of language performance and patronizing speech. *Journal of Language and Social Psychology, 36*(3), 287-305. https://doi.org/10.1177/0261927X16662967
2. Babbitt, L. G., Gaither, S. E., Toosi, N. R., & Sommers, S. R. (2018). The role of gender in racial meta-stereotypes and stereotypes. *Social Cognition, 36*(5), 589-601. https://doi.org/10.1521/soco.2018.36.5.589
3. Barlow, M. R., & Lahey, J. N. (2018). What race is Lacey? Intersecting perceptions of racial minority status and social class. *Social Science Quarterly, 99*(5), 1680-1698. https://doi.org/10.1111/ssqu.12529
4. Baumann, C., Timming, A. R., & Gollan, P. J. (2016). Taboo tattoos? A study of the gendered effects of body art on consumers' attitudes toward visibly tattooed front line staff. *Journal of Retailing and Consumer Services, 29*, 31-39. https://doi.org/10.1016/j.jretconser.2015.11.005
5. Bešić, E., Paleczek, L., & Gasteiger-Klicpera, B. (2020). Don’t forget about us: Attitudes towards the inclusion of refugee children with(out) disabilities. *International Journal of Inclusive Education, 24*(2), 202-217. https://doi.org/10.1080/13603116.2018.1455113
6. Bešić, E., Paleczek, L., Rossmann, P., Krammer, M., & Gasteiger-Klicpera, B. (2020). Attitudes towards inclusion of refugee girls with and without disabilities in Austrian primary schools. *International Journal of Inclusive Education, 24*(5), 463-478. https://doi.org/10.1080/13603116.2018.1467976
7. Biernacka, M., & Jancewicz, B. (2019). Ritual slaughter and feelings of threat amongst Polish young adults. A study on university students’ attitudes in the city of Bialystok. *Studia Migracyjne-Przegląd Polonijny, 45*(2) 172.
8. Bohlmann, C., & Zacher, H. (2020). Making things happen (un)expectedly: Interactive effects of age, gender, and motives on evaluations of proactive behavior. *Journal of Business and Psychology*, 1-23. https://doi.org/10.1007/s10869-020-09691-7
9. Bourabain, D., & Verhaeghe, P. P. (2019). Could you help me, please? Intersectional field experiments on everyday discrimination in clothing stores. *Journal of Ethnic and Migration Studies, 45*(11), 2026-2044. https://doi.org/10.1080/1369183X.2018.1480360

10. Brooks, J. A., Stolier, R. M., & Freeman, J. B. (2018). Stereotypes bias visual prototypes for sex and emotion categories. *Social Cognition*, *36*(5), 481-493. https://doi.org/10.1521/soco.2018.36.5.481
11. Bursell, M., & Jansson, F. (2018). Diversity preferences among employees and ethnoracial workplace segregation. *Social Science Research*, *74*, 62-76. https://doi.org/10.1016/j.ssresearch.2018.03.009
12. Carnaghi, A., Stragà, M., Coladonato, R., Bianchi, M., & Piccoli, V. (2020). Extrapolating stereotypical information on sexual orientation from race categories: The case of Black and Asian men. *Psychology of Men & Masculinities*, *21*(2), 224–234. https://doi.org/10.1037/men0000225
13. Chuang, R., Wilkins, C., Tan, M., & Mead, C. (2020). Racial minorities' attitudes toward interracial couples: An intersection of race and gender. *Group Processes & Intergroup Relations*, 1368430219899482. https://doi.org/10.1177/1368430219899482
14. Corrigan, P., Hafeez, M., & Alkhouja, M. (2018). How does immigration status affect the public stigma of behavioral health disorders?. *Journal of Public Mental Health*, *17*(4), 195-199. https://doi.org/10.1108/JPMH-04-2018-0026
15. Derous, E., & Pepermans, R. (2019). Gender discrimination in hiring: Intersectional effects with ethnicity and cognitive job demands. *Archives of Scientific Psychology*, *7*(1), 40-49. http://dx.doi.org/10.1037/arc0000061
16. Dias, F. A. (2020). How skin color, class status, and gender intersect in the labor market: Evidence from a field experiment. *Research in Social Stratification and Mobility*, *65*, 100477. https://doi.org/10.1016/j.rssm.2020.100477
17. Donovan, R. A. (2011). Tough or tender: (Dis)similarities in white college students' perceptions of Black and white women. *Psychology of Women Quarterly*, *35*(3), 458–468. https://doi.org/10.1177/0361684311406874
18. Eaton, A. A., Saunders, J. F., Jacobson, R. K., & West, K. (2020). How gender and race stereotypes impact the advancement of scholars in STEM: Professors' biased evaluations of physics and biology post-doctoral candidates. *Sex Roles*, *82*(3), 127-141. https://doi.org/10.1007/s11199-019-01052-w
19. Fasoli, F., Cadinu, M., Carnaghi, A., Galdi, S., Guizzo, F., & Tassara, L. (2018). How do you self-categorize? Gender and sexual orientation self-categorization in homosexual/heterosexual men and women. *Personality and Individual Differences*, *123*, 135-139. https://doi.org/10.1016/j.paid.2017.11.011
20. Fasoli, F., & Hegarty, P. (2020). A leader doesn't sound lesbian!: The impact of sexual orientation vocal cues on heterosexual persons' first impression and hiring decision. *Psychology of Women Quarterly*, *44*(2), 234-255. https://doi.org/10.1177/0361684319891168
21. Ghavami, N., & Mistry, R. S. (2019). Urban ethnically diverse adolescents' perceptions of social class at the intersection of race, gender, and sexual orientation. *Developmental Psychology*, *55*(3), 457–470. https://doi.org/10.1037/dev0000572

22. Ghavami, N., & Peplau, L. A. (2018). Urban middle school students' stereotypes at the intersection of sexual orientation, ethnicity, and gender. *Child Development*, *89*(3), 881-896. https://doi.org/10.1111/cdev.12763
23. Goff, P. A., Thomas, M. A., & Jackson, M. C. (2008). "Ain't I a woman?": Towards an intersectional approach to person perception and group-based harms. *Sex Roles*, *59*(5-6), 392-403. https://doi.org/10.1007/s11199-008-9505-4
24. Hall, E. V., Galinsky, A. D., & Phillips, K. W. (2015). Gender profiling: A gendered race perspective on person–position fit. *Personality and Social Psychology Bulletin*, *41*(6), 853-868. https://doi.org/10.1177/0146167215580779
25. Hester, N., & Gray, K. (2018). For Black men, being tall increases threat stereotyping and police stops. *Proceedings of the National Academy of Sciences*, *115*(11), 2711-2715. https://doi.org/10.1073/pnas.1714454115
26. Jensen, C. J., & Tisak, M. S. (2020). Precedents of prejudice: Race and gender differences in young children's intergroup attitudes. *Early Child Development and Care*, *190*(9), 1336-1349. https://doi.org/10.1080/03004430.2018.1534845
27. Jiang, C., Vitiello, C., Axt, J. R., Campbell, J. T., & Ratliff, K. A. (2019). An examination of ingroup preferences among people with multiple socially stigmatized identities. *Self and Identity*, 1-18. https://doi.org/10.1080/15298868.2019.1657937
28. Johnson, I. R., Pietri, E. S., Fullilove, F., & Mowrer, S. (2019). Exploring identity-safety cues and allyship among black women students in STEM environments. *Psychology of Women Quarterly*, *43*(2), 131-150. https://doi.org/10.1177/0361684319830926
29. Kim, H. I., Johnson, K. L., & Johnson, S. P. (2015). Gendered race: Are infants' face preferences guided by intersectionality of sex and race?. *Frontiers in Psychology*, *6*, 1330. https://doi.org/10.3389/fpsyg.2015.01330
30. Knowles, M. L., & Dean, K. K. (2018). Present but invisible: Physical obscurity fosters social disconnection. *European Journal of Social Psychology*, *48*(1), 86-92. https://doi.org/10.1002/ejsp.2274
31. Kulesza, M., Matsuda, M., Ramirez, J. J., Werntz, A. J., Teachman, B. A., & Lindgren, K. P. (2016). Towards greater understanding of addiction stigma: Intersectionality with race/ethnicity and gender. *Drug and Alcohol Dependence*, *169*, 85-91. https://doi.org/10.1016/j.drugalcdep.2016.10.020
32. Lick, D. J., & Johnson, K. L. (2018). Facial cues to race and gender interactively guide age judgments. *Social Cognition*, *36*(5), 497-516. https://doi.org/10.1521/soco.2018.36.5.497
33. Makowski, A. C., Kim, T. J., Luck-Sikorski, C., & von dem Knesebeck, O. (2019). Social deprivation, gender and obesity: Multiple stigma? Results of a population survey from Germany. *BMJ Open*, *9*(4), e023389. http://dx.doi.org/10.1136/bmjopen-2018-023389

34. McMahon, J. M., & Kahn, K. B. (2016). Benevolent racism? The impact of target race on ambivalent sexism. *Group Processes & Intergroup Relations*, *19(*2), 169-183. https://doi.org/10.1177/1368430215583153
35. Miller, A. K. (2019). "Should have known better than to fraternize with a Black man": Structural racism intersects rape culture to intensify attributions of acquaintance rape victim culpability. *Sex Roles,* 81(7), 428-438. https://doi.org/10.1007/s11199-019-1003-3
36. Miles-Johnson, T., & Death, J. (2020). Compensating for sexual identity: How LGB and heterosexual Australian police officers perceive policing of LGBTIQ+ people. *Journal of Contemporary Criminal Justice, 36*(2), 251-273. https://doi.org/10.1177/1043986219894431
37. Muriaas, R. L., Wang, V., Benstead, L., Dulani, B., & Rakner, L. (2019). Why the gender of traditional authorities matters: Intersectionality and women's rights advocacy in Malawi. *Comparative Political Studies, 52*(12), 1881-1924. https://doi.org/10.1177/0010414018774369
38. Ngoubene-Atioky, A. J., Williamson-Taylor, C., Inman, A. G., & Case, J. (2017). Psychotherapists' empathy for childfree women of intersecting age and socioeconomic status. *Journal of Mental Health Counseling, 39*(3), 211-224. https://doi.org/10.17744/mehc.39.3.03
39. Perszyk, D. R., Lei, R. F., Bodenhausen, G. V., Richeson, J. A., & Waxman, S. R. (2019). Bias at the intersection of race and gender: Evidence from preschool-aged children. *Developmental Science, 22*(3), e12788. https://doi.org/10.1111/desc.12788
40. Petsko, C. D., & Bodenhausen, G. V. (2019). Racial stereotyping of gay men: Can a minority sexual orientation erase race?. *Journal of Experimental Social Psychology, 83,* 37-54. https://doi.org/10.1016/j.jesp.2019.03.002
41. Phills, C. E., Williams, A., Wolff, J. M., Smith, A., Arnold, R., Felegy, K., & Kuenzig, M. E. (2018). Intersecting race and gender stereotypes: Implications for group-level attitudes. *Group Processes & Intergroup Relations, 21*(8), 1172-1184. https://doi.org/10.1177/1368430217706742
42. Pietri, E. S., Drawbaugh, M. L., Lewis, A. N., & Johnson, I. R. (2019). Who encourages Latina women to feel a sense of identity-safety in STEM environments?. Journal of Experimental Social Psychology, 84, 103827. https://doi.org/10.1016/j.jesp.2019.103827
43. Pietri, E. S., Johnson, I. R., Majid, S., & Chu, C. (2020). Seeing what's possible: Videos are more effective than written portrayals for enhancing the relatability of scientists and promoting Black female students' interest in STEM. *Sex Roles, 84*(1), 14-33. https://doi.org/10.1007/s11199-020-01153-x
44. Rattan, A., Steele, J., & Ambady, N. (2019). Identical applicant but different outcomes: The impact of gender versus race salience in hiring. *Group Processes & Intergroup Relations*, *22*(1), 80-97. https://doi.org/10.1177/1368430217722035

45. Remedios, J. D., Reiff, J. S., & Hinzman, L. (2020). An identity-threat perspective on discrimination attributions by women of color. *Social Psychological and Personality Science*, *11*(7), 889-898. https://doi.org/10.1177/1948550620908175
46. Remedios, J. D., & Snyder, S. H. (2018). The (in) efficiency of person construal involving intersectional social categories. *Social Cognition*, *36*(5), 517-533. https://doi.org/10.1521/soco.2018.36.5.517
47. Remedios, J. D., Snyder, S. H., & Lizza, C. A. (2016). Perceptions of women of color who claim compound discrimination: Interpersonal judgments and perceived credibility. *Group Processes & Intergroup Relations*, *19*(6), 769-783. https://doi.org/10.1177/1368430215591041
48. Riegle-Crumb, C., & Humphries, M. (2012). Exploring bias in math teachers' perceptions of students' ability by gender and race/ethnicity. *Gender & Society*, *26*(2), 290-322. https://doi.org/10.1177/0891243211434614
49. Rosenthal, L., & Lobel, M. (2016). Stereotypes of Black American women related to sexuality and motherhood. *Psychology of Women Quarterly*, *40*(3), 414-427. https://doi.org/10.1177/0361684315627459
50. Rosette, A. S., Koval, C. Z., Ma, A., & Livingston, R. (2016). Race matters for women leaders: Intersectional effects on agentic deficiencies and penalties. *The Leadership Quarterly*, *27*(3), 429-445. https://doi.org/10.1016/j.leaqua.2016.01.008
51. Rosette, A. S., & Livingston, R. W. (2012). Failure is not an option for Black women: Effects of organizational performance on leaders with single versus dual-subordinate identities. *Journal of Experimental Social Psychology*, *48*(5), 1162-1167. https://doi.org/10.1016/j.jesp.2012.05.002
52. Scheuer, C. L., & Loughlin, C. (2020). Could the aging workforce reduce the agency penalty for female leaders? Re-examining the think manager–think male stereotype. *Journal of Management and Organization*, *26*(1), 29-51. https://doi.org/10.1017/jmo.2018.41
53. Schug, J., Alt, N. P., & Klauer, K. C. (2015). Gendered race prototypes: Evidence for the non-prototypicality of Asian men and Black women. *Journal of Experimental Social Psychology*, *56*, 121-125. https://doi.org/10.1016/j.jesp.2014.09.012
54. Semrow, M., Zou, L. X., Liu, S., & Cheryan, S. (2020). Gay Asian Americans are seen as more American than Asian Americans who are presumed straight. *Social Psychological and Personality Science*, *11*(3), 336-344. https://doi.org/10.1177/1948550619849426
55. Sesko, A. K., & Biernat, M. (2018). Invisibility of Black women: Drawing attention to individuality. *Group Processes & Intergroup Relations*, *21*(1), 141-158. https://doi.org/10.1177/1368430216663017
56. Sheeran, N., Jones, L., & Perolini, J. (2019). Intersecting identities: How race and age influence perceptions of mothers. *Australian Journal of Psychology*, *71*(3), 261-272. https://doi.org/10.1111/ajpy.12243

57. Simpkins, J. (2014). Seeing white men: Bias in gender categorization. *Gender Issues, 31*(1), 21-33. https://doi.org/10.1007/s12147-014-9116-z
58. Smith, J. S., LaFrance, M., & Dovidio, J. F. (2017). Categorising intersectional targets: An "either/and" approach to race-and gender-emotion congruity. *Cognition and Emotion, 31*(1), 83-97. https://doi.org/10.1080/02699931.2015.1081875
59. Steinbugler, A. C., Press, J. E., & Dias, J. J. (2006). Gender, race, and affirmative action: Operationalizing intersectionality in survey research. *Gender & Society*, *20*(6), 805-825. https://doi.org/10.1177/0891243206293299
60. Thiem, K. C., Neel, R., Simpson, A. J., & Todd, A. R. (2019). Are Black women and girls associated with danger? Implicit racial bias at the intersection of target age and gender. *Personality and Social Psychology Bulletin*, *45*(10), 1427-1439. https://doi.org/10.1177/0146167219829182
61. Thomas, E. L., Dovidio, J. F., & West, T. V. (2014). Lost in the categorical shuffle: Evidence for the social non-prototypicality of Black women. *Cultural Diversity and Ethnic Minority Psychology*, *20*(3), 370–376. https://doi.org/10.1037/a0035096
62. Timberlake, J. M., & Estes, S. B. (2007). Do racial and ethnic stereotypes depend on the sex of target group members? Evidence from a survey-based experiment. S*ociological Quarterly*, *48*(3), 399-433. https://doi.org/10.1111/j.1533-8525.2007.00083.x
63. Tinkler, J., Zhao, J., Li, Y., & Ridgeway, C. L. (2019). Honorary whites? Asian American women and the dominance penalty. *Socius. 5*, 2378023119836000. https://doi.org/10.1177/2378023119836000
64. Todd, A. R., & Simpson, A. J. (2017). Perspective taking and member-to-group generalization of implicit racial attitudes: The role of target prototypicality. *European Journal of Social Psychology*, *47*(1), 105-112. https://doi.org/10.1002/ejsp.2204
65. Todd, A. R., Simpson, A. J., Thiem, K. C., & Neel, R. (2016). The generalization of implicit racial bias to young black boys: Automatic stereotyping or automatic prejudice?. *Social Cognition*, *34*(4), 306-323. https://doi.org/10.1521/soco.2016.34.4.306
66. Tresh, F., Steeden, B., Randsley de Moura, G., Leite, A. C., Swift, H. J., & Player, A. (2019). Endorsing and reinforcing gender and age stereotypes: The negative effect on self-rated leadership potential for women and older workers. *Frontiers in Psychology, 10*, 688. https://doi.org/10.3389/fpsyg.2019.00688
67. Varas-Díaz, N., Rivera-Segarra, E., Neilands, T. B., Pedrogo, Y., Carminelli-Corretjer, P., Tollinchi, N., Torres, E., Soto Del Valle, Y., Rivera Díaz, M., & Ortiz, N. (2019). HIV/AIDS and intersectional stigmas: Examining stigma related behaviours among medical students during service delivery. *Global Public Health, 14*(11), 1598–1611. https://doi.org/10.1080/17441692.2019.1633378
68. Wang, K., Walker, K., Pietri, E., & Ashburn-Nardo, L. (2019). Consequences of confronting patronizing help for people with disabilities: Do target gender and

disability type matter?. *The Journal of Social Issues, 75*(3), 904–923. https://doi.org/10.1111/josi.12332
69. Williams, S. L. L., Cabrera-Nguyen, E. P., & Johnson, S. D. (2018). Examining the impact of race/ethnicity and gender intersectionality on preferences of social distance from individuals with mental health conditions. *Journal of Human Behavior in the Social Environment*, *28*(3), 370-380. https://doi.org/10.1080/10911359.2018.1433572
70. Wilson, J. P., Remedios, J. D., & Rule, N. O. (2017). Interactive effects of obvious and ambiguous social categories on perceptions of leadership: When double-minority status may be beneficial. *Personality and Social Psychology Bulletin, 43*(6), 888-900. https://doi.org/10.1177/0146167217702373
71. Zimmermann, C. R. (2018). The penalty of being a young black girl: Kindergarten teachers' perceptions of children's problem behaviors and student–teacher conflict by the intersection of race and gender. *The Journal of Negro Education*, *87*(2), 154-168. https://www.jstor.org/stable/10.7709/jnegroeducation.87.2.0154

B.2. Bibliography: Excluded studies—Development or validation of intersectional measures

1. Bastos, J. L., Reichenheim, M. E., Celeste, R. K., Faerstein, E., Barros, A. J. D., & Paradies, Y. C. (2019). Perceived discrimination south of the equator: Reassessing the Brazilian Explicit Discrimination Scale. *Cultural Diversity and Ethnic Minority Psychology*, *25*(3), 413–423. https://doi.org/10.1037/cdp0000246
2. Bowleg, L., English, D., Del Rio-Gonzalez, A. M., Burkholder, G. J., Teti, M., & Tschann, J. M. (2016). Measuring the pros and cons of what it means to be a Black man: Development and validation of the Black Men's Experiences Scale (BMES). *Psychology of Men & Masculinity*, *17*(2), 177–188. https://doi.org/10.1037/men0000026
3. Bradley, A., Mennie, N., Bibby, P. A., & Cassaday, H. J. (2020). Some animals are more equal than others: Validation of a new scale to measure how attitudes to animals depend on species and human purpose of use. *PloS One*, *15*(1), e0227948. https://doi.org/10.1371/journal.pone.0227948
4. Brown, D. L., Blackmon, S. K., Rosnick, C. B., Griffin-Fennell, F. D., & White-Johnson, R. L. (2017). Initial development of a gendered-racial socialization scale for African American college women. *Sex Roles*, *77*(3), 178-193. https://doi.org/10.1007/s11199-016-0707-x
5. Celis, K., & Mügge, L. M. (2018). Whose equality? Measuring group representation. *Politics*, *38*(2), 197-213. https://doi.org/10.1177/0263395716684527
6. Friedland, B. A., Sprague, L., Nyblade, L., Baral, S. D., Pulerwitz, J., Gottert, A., Amanyeiwe, U., Cheng, A., Mallouris, C., Anam, F., Jackson, A., & Geibel, S. (2018).

Measuring intersecting stigma among key populations living with HIV: Implementing the people living with HIV Stigma Index 2.0. *Journal of the International AIDS Society*, *21*(5), e25131. https://doi.org/10.1002/jia2.25131

7. Galupo, M. P., Mitchell, R. C., & Davis, K. S. (2018). Face validity ratings of sexual orientation scales by sexual minority adults: Effects of sexual orientation and gender identity. *Archives of Sexual Behavior, 47*(4), 1241-1250. https://doi.org/10.1007/s10508-017-1037-y
8. Gamst, G., Arellano-Morales, L., Meyers, L. S., Tolstoy, B., Garcia, S., Sjobeck, G., Blair, R., & Casas, M. (2019). Development and validation of the Latina American Shifting Scale (LASS). *Gender Issues*, *36*(3), 269–294. https://doi.org/10.1007/s12147-018-9225-1
9. Grzanka, P. R., Miles, J. R., Spengler, E. S., Arnett, J. E., & Pruett, J. (2020). Measuring neoliberalism: Development and initial validation of a scale of anti-neoliberal attitudes. *Social Justice Research, 33*(1), 44-80. https://doi.org/10.1007/s11211-019-00339-3
10. Keum, B. T., Brady, J. L., Sharma, R., Lu, Y., Kim, Y. H., & Thai, C. J. (2018). Gendered racial microaggressions scale for Asian American women: Development and initial validation. *Journal of Counseling Psychology, 65*(5), 571-585. https://doi.org/10.1037/cou0000305
11. Keum, B. T., & Miller, M. J. (2018). Measurement invariance of the perceived online racism scale across age and gender. *Cyberpsychology: Journal of Psychosocial Research on Cyberspace, 12*(3). https://doi.org/10.5817/CP2018-3-3
12. Lewis, J. A., & Neville, H. A. (2015). Construction and initial validation of the Gendered Racial Microaggressions Scale for Black women. *Journal of Counseling Psychology*, *62*(2), 289–302. https://doi.org/10.1037/cou0000062
13. Li, X. M., Rasooly, A., Peng, B., & Xiong, S. Y. (2017). An analysis on intersectional collaboration on non-communicable chronic disease prevention and control in China: A cross-sectional survey on main officials of community health service institutions. *BMC health services research, 17*(1), 1-10. https://doi.org/10.1186/s12913-017-2654-9
14. Liu, T., Wong, Y. J., Maffini, C. S., Goodrich Mitts, N., & Iwamoto, D. K. (2018). Gendered racism scales for Asian American men: Scale development and psychometric properties. *Journal of Counseling Psychology, 65*(5), 556–570. https://doi.org/10.1037/cou0000298
15. McEntee, M. L., Serier, K. N., Smith, J. M., & Smith, J. E. (2020). The sum is greater than its parts: Intersectionality and measurement validity of the Eating Disorder Examination Questionnaire (EDE-Q) in Latinx undergraduates in the United States. *Sex Roles, 84*(1), 102-111. https://doi.org/10.1007/s11199-020-01149-7
16. Scheim, A. I., & Bauer, G. R. (2019). The Intersectional Discrimination Index: Development and validation of measures of self-reported enacted and anticipated

discrimination for intercategorical analysis. *Social Science & Medicine*, *226*, 225-235. https://doi.org/10.1016/j.socscimed.2018.12.016
17. Schrager, S. M., Goldbach, J. T., & Mamey, M. R. (2018). Development of the sexual minority adolescent stress inventory. *Frontiers in Psychology, 9,* 319. https://doi.org/10.3389/fpsyg.2018.00319
18. Shin, R. Q., Smith, L. C., Lu, Y., Welch, J. C., Sharma, R., Vernay, C. N., & Yee, S. (2018). The development and validation of the Contemporary Critical Consciousness Measure II. *Journal of Counseling Psychology*, *65*(5), 539–555. https://doi.org/10.1037/cou0000302

B.3. Bibliography: Original application papers for quantitative intersectionality

1. Abbasian, S., & Hellgren, C. (2012). Working conditions for female and immigrant cleaners in Stockholm County - An intersectional approach. *Nordic Journal of Working Life Studies*, *2*(3), 1–21. https://doi.org/10.19154/njwls.v2i3.2369
2. Abichahine, H., & Veenstra, G. (2017). Inter-categorical intersectionality and leisure-based physical activity in Canada. *Health Promotion International*, *32*(4), 691–701. https://doi.org/10.1093/heapro/daw009
3. Adams, G., & Kurtiş, T. (2015). Friendship and gender in cultural-psychological perspective: Implications for research, practice, and consultation. *International Perspectives in Psychology: Research, Practice, Consultation*, *4*(3), 182–194. https://doi.org/10.1037/ipp0000036
4. Agénor, M., Abboud, S., Delgadillo, J. G., Pérez, A. E., Peitzmeier, S. M., & Borrero, S. (2018). Intersectional nativity and racial/ethnic disparities in human papillomavirus vaccination initiation among U.S. women: A national population-based study. *Cancer Causes Control*, *29*(10), 927–936. https://doi.org/10.1007/s10552-018-1069-1
5. Agénor, M., Pérez, A. E., Koma, J. W., Abrams, J. A., McGregor, A. J., & Ojikutu, B. O. (2019). Sexual orientation identity, race/ethnicity, and lifetime HIV testing in a national probability sample of U.S. women and men: An intersectional approach. *LGBT Health*, *6*(6), 306–318. https://doi.org/10.1089/lgbt.2019.0001
6. Agénor, M., Pérez, A. E., Peitzmeier, S. M., Potter, J., & Borrero, S. (2018). Human papillomavirus vaccination initiation among sexual orientation identity and racial/ethnic subgroups of Black and white U.S. women and girls: An intersectional analysis. *Journal of Women's Health*, *27*(11), 1349–1358. https://doi.org/10.1089/jwh.2017.6768
7. Agénor, M., Krieger, N., Austin, S. B., Haneuse, S., & Gottlieb, B. R. (2014). At the intersection of sexual orientation, race/ethnicity, and cervical cancer screening: Assessing Pap test use disparities by sex of sexual partners among Black, Latina, and white U.S. women. *Social Science and Medicine*, *116*, 110–118. https://doi.org/10.1016/j.socscimed.2014.06.039
8. Aguirre, C. G., Bello, M. S., Andrabi, N., Pang, R. D., Hendricks, P. S., Bluthenthal, R. N., & Leventhal, A. M. (2016). Gender, ethnicity, and their intersectionality in the

prediction of smoking outcome expectancies in regular cigarette smokers. *Behavior Modification*, *40*(1-2), 281–302. https://doi.org/10.1177/0145445515608146
9. Ailshire, J. A., & House, J. S. (2011). The unequal burden of weight gain: An intersectional approach to understanding social disparities in BMI trajectories from 1986 to 2001/2002. *Social Forces*, *90*(2), 397–423. https://doi.org/10.1093/sf/sor001
10. Al Dabbagh, M., Bowles, H. R., & Thomason, B. (2016). Status reinforcement in emerging economies: The psychological experience of local candidates striving for global employment. *Organization Science*, *27*(6), 1453–1471. https://doi.org/10.1287/orsc.2016.1099
11. Alberton, A. M., Angell, G. B., Gorey, K. M., & Grenier, S. (2020). Homelessness among Indigenous peoples in Canada: The impacts of child welfare involvement and educational achievement. *Children and Youth Services Review*, *111*, 104846. https://doi.org/10.1016/j.childyouth.2020.104846
12. Alberton, A. M., & Gorey, K. M. (2017). Profound barriers to basic cancer care most notably experienced by uninsured women: Historical note on the present policy considerations. *Social Work in Health Care*, *56*(10), 943–949. https://doi.org/10.1080/00981389.2017.1373724
13. Algarin, A. B., Zhou, Z., Cook, C. L., Cook, R. L., & Ibañez, G. E. (2019). Age, sex, race, ethnicity, sexual orientation: Intersectionality of marginalized-group identities and enacted HIV-related stigma among people living with HIV in Florida. *AIDS and Behavior*, *23*(11), 2992–3001. https://doi.org/10.1007/s10461-019-02629-y
14. Allen, J., Smith, J. L., Thoman, D. B., & Walters, R. W. (2018). Fluctuating team science: Perceiving science as collaborative improves science motivation. *Motivation Science*, *4*(4), 347–361. https://doi.org/10.1037/mot0000099
15. Allen, S. H., & Leslie, L. A. (2019). Considering the role of nativity in the health and psychological wellbeing of Black LGBT adults. *Journal of Homosexuality*, *66*(13), 1769–1796. https://doi.org/10.1080/00918369.2018.1511134
16. Allison, J. E., Herrera, J. S., Struna, J., & Reese, E. (2018). The matrix of exploitation and temporary employment: Earnings inequality among Inland Southern California's blue-collar warehouse workers. *Journal of Labor and Society*, *21*(4), 533–560. https://doi.org/10.1111/wusa.12366
17. Allison, R. (2011). Race, gender, and attitudes toward war in Chicago: An intersectional analysis. *Sociological Forum*, *26*(3), 668–691. https://doi.org/10.1111/j.1573-7861.2011.01267.x
18. Almazan, E. P. (2019). Are Black sexual minority adults more likely to report higher levels of psychological distress than white sexual minority adults? Findings from the 2013-2017 National Health Interview Survey. *Social Sciences*, *8*(1) 14. https://doi.org/10.3390/socsci8010014
19. Alon, S. (2007). Overlapping disadvantages and the racial/ethnic graduation gap among students attending selective institutions. *Social Science Research*, *36*(4), 1475–1499. https://doi.org/10.1016/j.ssresearch.2007.01.006
20. Alves, R. F. S., & Faerstein, E. (2016). Educational inequalities in hypertension: Complex patterns in intersections with gender and race in Brazil. *International Journal for Equity in Health*, *15*(1) 1-9. https://doi.org/10.1186/s12939-016-0441-6

21. Ammons, S. K., Dahlin, E. C., Edgell, P., & Santo, J. B. (2016). Work–family conflict among Black, white, and Hispanic men and women. *Community, Work and Family*, *20*(4), 379-404. https://doi.org/10.1080/13668803.2016.1146231
22. Amroussia, N., Gustafsson, P. E., & Pearson, J. L. (2020). Do inequalities add up? Intersectional inequalities in smoking by sexual orientation and education among U.S. adults. *Preventive Medicine Reports*, *17*, 101032. https://doi.org/10.1016/j.pmedr.2019.101032
23. Amroussia, N., Pearson, J. L., & Gustafsson, P. E. (2019). What drives us apart? Decomposing intersectional inequalities in cigarette smoking by education and sexual orientation among U.S. adults. *International Journal for Equity in Health, 18*(1), 1-14. https://doi.org/10.1186/s12939-019-1015-1
24. Andersson, M., Ivert, A.-K., & Mellgren, C. (2018). When there is more than one motive: A study on self-reported hate crime victimization among Swedish university students. *International Review of Victimology*, *24*(1), 67–81. https://doi.org/10.1177/0269758017736393
25. Andrade, S. B., & Järvinen, M. (2017). More risky for some than others: Negative life events among young risk-takers. *Health, Risk & Society*, *19*(7-8), 387–410. https://doi.org/10.1080/13698575.2017.1413172
26. Ang, S. (2019). Intersectional cohort change: Disparities in mobility limitations among older Singaporeans. *Social Science & Medicine*, *228*, 223–231. https://doi.org/10.1016/j.socscimed.2019.03.039
27. Anyikwa, V. A. (2015). The intersections of race and gender in help-seeking strategies among a battered sample of low-income African American women. *Journal of Human Behavior in the Social Environment*, *25*(8), 948–959. https://doi.org/10.1080/10911359.2015.1047075
28. Aranda, F., Matthews, A. K., Hughes, T. L., Muramatsu, N., Wilsnack, S. C., Johnson, T. P., & Riley, B. B. (2015). Coming out in color: Racial/ethnic differences in the relationship between level of sexual identity disclosure and depression among lesbians. *Cultural Diversity and Ethnic Minority Psychology*, *21*(2), 247–257. https://doi.org/10.1037/a0037644
29. Aspinall, P. J., & Song, M. (2013). Is race a "salient..." or "dominant identity" in the early 21st century: The evidence of UK survey data on respondents' sense of who they are. *Social Science Research*, *42*(2), 547–561. https://doi.org/10.1016/j.ssresearch.2012.10.007
30. Assari, S. (2018). Parental education attainment and educational upward mobility; role of race and gender. *Behavioral Sciences*, *8*(11) 107. https://doi.org/10.3390/bs8110107
31. Assari, S., Lankarani, M. M., Piette, J. D., & Aikens, J. E. (2018). Self-rated health and glycemic control in type 2 diabetes: Race by gender differences. *Journal of Racial and Ethnic Health Disparities*, *5*(4), 721–727. https://doi.org/10.1007/s40615-017-0416-3
32. Assari, S., Nikahd, A., Malekahmadi, M. R., Lankarani, M. M., & Zamanian, H. (2017). Race by gender group differences in the protective effects of socioeconomic factors against sustained health problems across five domains. *Journal of Racial and Ethnic Health Disparities*, *4*(5), 884–894. https://doi.org/10.1007/s40615-016-0291-3
33. Augustyn, M. B., & Jackson, D. B. (2020). An intersectional look at the "rush to adulthood": Considering the role of gender, race and SES in the link between

precocious transitions and adult antisocial behavior. *Youth & Society*, *52*(1), 3–26. https://doi.org/10.1177/0044118X17725245
34. Badas, A., & Stauffer, K. E. (2019). Michelle Obama as a political symbol: Race, gender, and public opinion toward the first lady. *Politics & Gender*, *15*(3), 431–459. https://doi.org/10.1017/S1743923X18000922
35. Baiden, P., LaBrenz, C. A., Asiedua-Baiden, G., & Muehlenkamp, J. J. (2020). Examining the intersection of race/ethnicity and sexual orientation on suicidal ideation and suicide attempt among adolescents: Findings from the 2017 Youth Risk Behavior Survey. *Journal of Psychiatric Research*, *125*, 13–20. https://doi.org/10.1016/j.jpsychires.2020.02.029
36. Baker, J. O., & Whitehead, A. L. (2016). Gendering (non)religion: Politics, education, and gender gaps in secularity in the United States. *Social Forces*, *94*(4), 1623–1645. https://doi.org/10.1093/sf/sov119
37. Ballo, J. G. (2020). Labour market participation for young people with disabilities: The Impact of gender and higher education. *Work, Employment, and Society*, *34*(2), 336–355. https://doi.org/10.1177/0950017019868139
38. Barden, E. P., Barry, R. A., Khalifian, C. E., & Bates, J. M. (2016). Sociocultural influences on positive affect: Social support adequacy from one's spouse and the intersections of race and SES. *Journal of Social and Clinical Psychology*, *35*(6), 455–470. https://doi.org/10.1521/jscp.2016.35.6.455
39. Barefoot, K. N., Warren, J. C., & Smalley, K. B. (2017). Women's health care: The experiences and behaviors of rural and urban lesbians in the USA. *Rural and Remote Health*, *17*(1) 111.
40. Bares, C. B., Weaver, A., & Kelso, M. F. (2019). Adolescent opioid use: Examining the intersection of multiple inequalities. *Journal of Prevention & Intervention in the Community*, *47*(4), 295–309. https://doi.org/10.1080/10852352.2019.1617382
41. Baskin-Sommers, A. R., Baskin, D. R., Sommers, I. B., & Newman, J. P. (2013). The intersectionality of sex, race, and psychopathology in predicting violent crimes. *Criminal Justice and Behavior*, *40*(10), 1068–1091. https://doi.org/10.1177/0093854813485412
42. Bastos, J. L., Harnois, C. E., & Paradies, Y. C. (2018). Health care barriers, racism, and intersectionality in Australia. *Social Science & Medicine*, *199*, 209–218. https://doi.org/10.1016/j.socscimed.2017.05.010
43. Battle, J., Alderman-Swain, W., & Tyner, A. R. (2005). Using an intersectionality model to explain the educational outcomes for Black students in a variety of family configurations. *Race, Gender & Class*, *12*(1), 126–151. https://www.jstor.org/stable/41675153
44. Battle, J., & Linville, D. (2006). Race, sexuality and schools: A quantitative assessment of intersectionality. *Race, Gender & Class*, *13*(3/4), 180-199. https://www.jstor.org/stable/41675180
45. Battle, J., & Smiley, C. (2020). Familia y educación: a quantitative assessment of the impact of parental configuration on educational attainment for a national sample of Latinx students. *Race Ethnicity and Education*, *23*(1), 21–38. https://doi.org/10.1080/13613324.2018.1497963

46. Battle, J. L., & Browne, A. P. (2018). The relative importance of parental configuration for Latinx students' educational attainment. *Hispanic Journal of Behavioral Sciences*, *40*(3), 351–368. https://doi.org/10.1177/0739986318777464
47. Baumgartner, F. R., Bell, K., Beyer, L., Boldrin, T., Doyle, L., Govan, L., Halpert, J., Hicks, J., Kyriakoudes, K., Lee, C., Leger, M., McAdon, S., Michalak, S., Murphy, C., Neal, E., O'Malley, O., Payne, E., Sapirstein, A., Stanley, S., & Thacker, K. (2020). Intersectional encounters, representative bureaucracy, and the routine traffic stop. *Policy Studies Journal*. https://doi.org/10.1111/psj.12382
48. Bécares, L., & Priest, N. (2015). Understanding the influence of race/ethnicity, gender, and class on inequalities in academic and non-academic outcomes among eighth-grade students: Findings from an intersectionality approach. *PLoS ONE*, *10*(10), e0141363. https://doi.org/10.1371/journal.pone.0141363
49. Beccia, A. L., Baek, J., Jesdale, W. M., Austin, S. B., Forrester, S., Curtin, C., & Lapane, K. L. (2019). Risk of disordered eating at the intersection of gender and racial/ethnic identity among U.S. high school students. *Eating Behaviors*, *34*, 101299. https://doi.org/10.1016/j.eatbeh.2019.05.002
50. Bedolla, L. G., & Scola, B. (2006). Finding intersection: Race, class, and gender in the 2003 California Recall Vote. *Politics & Gender*, *2*(1), 5–27. http://dx.doi.org/10.1017/S1743923X06066003X
51. Bell, C. N., & Blackman Carr, L. T. (2020). The role of weight perception in race differences in BMI among college graduate and non–college graduate women. *Obesity*, *28*(5), 970–976. https://doi.org/10.1002/oby.22765
52. Bell, K. E. (2013). Young adult offending: Intersectionality of gender and race. *Critical Criminology*, *21*(1), 103–121. https://doi.org/10.1007/s10612-012-9170-3
53. Beltran, T., Allen, A. M., Lin, J., Turner, C., Ozer, E. J., & Wilson, E. C. (2019). Intersectional discrimination is associated with housing instability among trans women living in the San Francisco Bay Area. *International Journal of Environmental Research and Public Health*, *16*(22), 4521. https://doi.org/10.3390/ijerph16224521
54. Bengiamin, M. I., Capitman, J. A., & Ruwe, M. B. (2010). Disparities in initiation and adherence to prenatal care: Impact of insurance, race-ethnicity and nativity. *Maternal and Child Health Journal*, *14*(4), 618–624. https://doi.org/10.1007/s10995-009-0485-y
55. Berdahl, T. A., & McQuillan, J. (2018). Self-rated health trajectories among married Americans: Do disparities persist over 20 years? *Journal of Aging Research*, *2018*. https://doi.org/10.1155/2018/1208598
56. Berg, J A. (2010). Race, class, gender, and social space: Using an intersectional approach to study immigration attitudes. *Sociological Quarterly*, *51*(2), 278–302. https://doi.org/10.1111/j.1533-8525.2010.01172.x
57. Berg, J. A., & Morley, S. (2014). Intersectionality and the foreign-born: explaining the variation in the immigration attitudes of immigrants. *Race, Gender & Class*, *21*(3/4), 32–47. https://www.jstor.org/stable/43496983
58. Bergersen, M., Klar, S., & Schmitt, E. (2018). Intersectionality and engagement among the LGBTQ+ community. *Journal of Women Politics, & Policy*, *39*(2), 196–219. https://doi.org/10.1080/1554477X.2018.1449527
59. Berrington, A., Roberts, S., & Tammes, P. (2016). Educational aspirations among UK young teenagers: Exploring the role of gender, class and ethnicity. *British Educational Research Journal*, *42*(5), 729–755. https://doi.org/10.1002/berj.3235

60. Best, R. K., Edelman, L. B., Krieger, L. H., & Eliason, S. R. (2011). Multiple disadvantages: An empirical test of intersectionality theory in eeo litigation. *Law and Society Review*, *45*(4), 991–1025. https://doi.org/10.1111/j.1540-5893.2011.00463.x
61. Bey, G. S., Jesdale, B., Forrester, S., Person, S. D., & Kiefe, C. (2019). Intersectional effects of racial and gender discrimination on cardiovascular health vary among Black and white women and men in the CARDIA study. *SSM Population Health, 8,* 100446. https://doi.org/10.1016/j.ssmph.2019.100446
62. Bey, G. S., Person, S. D., & Kiefe, C. (2020). Gendered race and setting matter: Sources of complexity in the relationships between reported interpersonal discrimination and cardiovascular health in the CARDIA Study. *Journal of Racial and Ethnic Health Disparities*, 1-11. https://doi.org/10.1007/s40615-020-00699-6
63. Biernat, M., & Sesko, A. K. (2013). Evaluating the contributions of members of mixed-sex work teams: Race and gender matter. *Journal of Experimental Social Psychology*, *49*(3), 471–476. https://doi.org/10.1016/j.jesp.2013.01.008
64. Bijedić, T., & Piper, A. (2019). Different strokes for different folks: The job satisfaction of the self-employed and the intersection of gender and migration background. *International Journal of Gender and Entrepreneurship*, *11*(3), 227–247. https://doi.org/10.1108/IJGE-01-2019-0021
65. Birkett, M., Neray, B., Janulis, P., Phillips II, G., & Mustanski, B. (2019). Intersectional identities and HIV: Race and ethnicity drive patterns of sexual mixing. *AIDS and Behavior*, *23*(6), 1452–1459. https://doi.org/10.1007/s10461-018-2270-7
66. Black, P., & Joseph, L. J. (2014). Still dazed and confused: Midlife marijuana use by the baby boom generation. *Deviant Behavior*, *35*(10), 822–841. https://doi.org/10.1080/01639625.2014.889994
67. Blankenship, B. T., & Stewart, A. J. (2017). Intersectional identities, identity dimensions, and academic contingencies of self-worth. *Identity*, *17*(3), 109–124. https://doi.org/10.1080/15283488.2017.1340159
68. Blom, N., Huijts, T., & Kraaykamp, G. (2016). Ethnic health inequalities in Europe. The moderating and amplifying role of healthcare system characteristics. *Social Science and Medicine*, *158*, 43–51. https://doi.org/10.1016/j.socscimed.2016.04.014
69. Blumell, L. E., & Rodriguez, N. S. (2020). Ambivalent sexism and gay men in the US and UK. *Sexuality & Culture*, *24*(1), 209–229. https://doi.org/10.1007/s12119-019-09635-1
70. Boggess, L. N., Powers, R. A., & Chamberlain, A. W. (2018). Sex, race, and place: Taking an intersectional approach to understanding neighborhood-level violent crime across race and sex. *Journal of Research in Crime and Delinquency*, *55*(4), 493–537. https://doi.org/10.1177/0022427818770790
71. Bones, P. D. C. (2013). Perceptions of vulnerability: A target characteristics approach to disability, gender, and victimization. *Deviant Behavior*, *34*(9), 727–750. https://doi.org/10.1080/01639625.2013.766511
72. Booysen, L. A. E., & Nkomo, S. M. (2010). Gender role stereotypes and requisite management characteristics: The case of South Africa. *Gender in Management*, *25*(4), 285–300. https://doi.org/10.1108/17542411011048164
73. Borowski, E., & Stathopoulos, A. (2020). On-demand ridesourcing for urban emergency evacuation events: An exploration of message content, emotionality, and

intersectionality. *International Journal of Disaster Risk Reduction*, *44*, 101406. https://doi.org/10.1016/j.ijdrr.2019.101406

74. Bostwick, W. B., Hughes, T. L., Steffen, A., Veldhuis, C. B., & Wilsnack, S. C. (2019). Depression and victimization in a community sample of bisexual and lesbian women: An intersectional approach. *Archives of Sexual Behavior*, *48*(1), 131–141. https://doi.org/10.1007/s10508-018-1247-y
75. Bouris, A., & Hill, B. J. (2017). Exploring the mother–adolescent relationship as a promotive resource for sexual and gender minority youth. *Journal of Social Issues*, *73*(3), 618–636. https://doi.org/10.1111/josi.12234
76. Bratton, K. A., Haynie, K. L., & Reingold, B. (2007). Agenda setting and African American women in state legislatures. *Journal of Women, Politics and Policy*, *28*(3-4), 71–96. https://doi.org/10.1300/J501v28n03_04
77. Brondolo, E., Rahim, R., Grimaldi, S. J., Ashraf, A., Bui, N., & Schwartz, J. C. (2015). Place of birth effects on self-reported discrimination: Variations by type of discrimination. *International Journal of Intercultural Relations*, *49*, 212–222. https://doi.org/10.1016/j.ijintrel.2015.10.001
78. Brooks, W. T., & Redlin, M. (2009). Occupational aspirations, rural to urban migration, and intersectionality: A comparison of white, Black, and Hispanic male and female group chances for leaving rural counties. *Southern Rural Sociology*, *24*(1), 130–152.
79. Brown, D. L., Blackmon, S., & Shiflett, A. (2018). Safer sexual practices among African American women: intersectional socialisation and sexual assertiveness. *Culture Health & Sexuaity*, *20*(6), 673–689. https://doi.org/10.1080/13691058.2017.1370132
80. Brown, R. L. (2014). Psychological distress and the intersection of gender and physical disability: Considering gender and disability-related risk factors. *Sex Roles*, *71*(3-4), 171–181. https://doi.org/10.1007/s11199-014-0385-5
81. Brown, R. L., & Moloney, M. E. (2019). Intersectionality, work, and well-being: The effects of gender and disability. *Gender & Society*, *33*(1), 94–122. https://doi.org/10.1177/0891243218800636
82. Brown, T. (2012). The intersection and accumulation of racial and gender inequality: Black women's wealth trajectories. *Review of Black Political Economy*, *39*(2), 239–258. https://doi.org/10.1007/s12114-011-9100-8
83. Brown, T. H. (2018). Racial stratification, immigration, and health inequality: A life course-intersectional approach. *Social Forces*, *96*(4), 1507–1540. https://doi.org/10.1093/sf/soy013
84. Brown, T. H., & Hargrove, T. W. (2018). Psychosocial mechanisms underlying older Black men's health. *The Journals of Gerontology: Series B, 73*(2), 188-197. https://doi.org/10.1093/geronb/gbx091
85. Brown, T. H., Richardson, L. J., Hargrove, T. W., & Thomas, C. S. (2016). Using multiple-hierarchy stratification and life course approaches to understand health inequalities: The intersecting consequences of race, gender, SES, and age. *Journal of Health and Social Behavior*, *57*(2), 200–222. https://doi.org/10.1177/0022146516645165
86. Browne, A. P., & Battle, J. (2018). Black family structure and educational outcomes: The role of household structure and intersectionality. *Journal of African American Studies*, *22*(1), 77–93. https://doi.org/10.1007/s12111-018-9395-7

87. Bruch, S. K., & Soss, J. (2018). Schooling as a formative political experience: Authority relations and the education of citizens. *Perspectives on Politics*, *16*(1), 36–57. https://doi.org/10.1017/S1537592717002195
88. Brynin, M., Longhi, S., & Zwysen, W. (2019). The diversification of inequality. *The British Journal of Sociology*, *70*(1), 70–89. https://doi.org/10.1111/1468-4446.12341
89. Bubriski-McKenzie, A., & Jasinski, J. L. (2013). Mental jealth effects of intimate terrorism and situational couple violence among Black and Hispanic women. *Violence Against Women*, *19*(12), 1429–1448. https://doi.org/10.1177/1077801213517515
90. Buchanan, N. T., Settles, I. H., Wu, I. H. C., & Hayashino, D. S. (2018). Sexual harassment, racial harassment, and well-being among Asian American women: An intersectional approach. *Women & Therapy*, *41*(3-4), 261–280. https://doi.org/10.1080/02703149.2018.1425030
91. Buckley, T. R. (2018). Black adolescent males: Intersections among their gender role identity and racial identity and associations with self-concept (global and school). *Child Development*, *89*(4), e311–e322. https://doi.org/10.1111/cdev.12950
92. Budge, S. L., Thai, J. L., Tebbe, E. A., & Howard, K. A. S. (2016). The intersection of race, sexual orientation, socioeconomic status, trans identity, and mental health outcomes. *Counseling Psychologist*, *44*(7), 1025–1049. https://doi.org/10.1177/0011000015609046
93. Buffarini, R., Abdalla, S., Weber, A. M., Costa, J. C., Menezes, A. M. B., Gonçalves, H., Wehrmeister, F. C., Meausoone, V., Darmstadt, G. L., & Victora, C. G. (2020). The intersectionality of gender and wealth in adolescent health and behavioral outcomes in Brazil: The 1993 Pelotas Birth Cohort. *Journal of Adolescent Health*, *66*(1), S51–S57. https://doi.org/10.1016/j.jadohealth.2019.08.029
94. Bunyasi, T. L., & Smith, C. W. (2019). Do all Black lives matter equally to Black people? Respectability politics and the limitations of linked fate. *Journal of Race, Ethnicity, and Politics*, *4*(1), 180–215. https://doi.org/10.1017/rep.2018.33
95. Burgard, S., Castiglione, D. P., Lin, K. Y., Nobre, A. A., Aquino, E. M. L., Pereira, A. C., Bensenor, I. J. M., Barreto, S. M., & Chor, D. (2017). Differential reporting of discriminatory experiences in Brazil and the United States. *Cadernos de Saude Publica*, *33*, e00110516. https://doi.org/10.1590/0102-311X00110516
96. Burns, V. L., Eaton, A. A., Long, H., & Zapp, D. (2019). Exploring the role of race and gender on perceived bystander ability and intent: Findings before and after exposure to an online training program to prevent sexual assault on campus. *Violence Against Women*, *25*(8), 999–1017. https://doi.org/10.1177/1077801218807089
97. Button, D. M., & Worthen, M. G. F. (2014). General strain theory for LGBQ and SSB youth: The importance of intersectionality in the future of feminist criminology. *Feminist Criminology*, *9*(4), 270–297. https://doi.org/10.1177/1557085114525988
98. Byars-Winston, A., & Rogers, J. G. (2019). Testing intersectionality of race/ethnicity × gender in a social-cognitive career theory model with science identity. *Journal of Counseling Psychology*, *66*(1), 30–44. https://doi.org/10.1037/cou0000309.supp
99. Byrd, C. M., & Carter Andrews, D. J. (2016). Variations in students' perceived reasons for, sources of, and forms of in-school discrimination: A latent class analysis. *Journal of School Psychology*, *57*, 1–14. https://doi.org/10.1016/j.jsp.2016.05.001
100. Byrd, K. M., Kahle, L. L., Peguero, A. A., & Popp, A. M. (2015). Social control and intersectionality: A multilevel analysis of school misconduct, location, race, ethnicity,

and sex. *Sociological Spectrum*, *35*(2), 109–135. https://doi.org/10.1080/02732173.2014.1000552
101. Caceres, B. A., Veldhuis, C. B., & Hughes, T. L. (2019). Racial/ethnic differences in cardiometabolic risk in a community sample of sexual minority women. *Health Equity*, *3*(1), 350–359. https://doi.org/10.1089/heq.2019.0024
102. Caceres, B. A., Hickey, K. T., Heitkemper, E. M., & Hughes, T. L. (2019). An intersectional approach to examine sleep duration in sexual minority adults in the United States: Findings from the Behavioral Risk Factor Surveillance System. *Sleep Health*, *5*(6), 621–629. https://doi.org/10.1016/j.sleh.2019.06.006
103. Caceres, B. A., Ancheta, A. J., Dorsen, C., Newlin-Lew, K., Edmondson, D., & Hughes, T. L. (2020). A population-based study of the intersection of sexual identity and race/ethnicity on physiological risk factors for CVD among U.S. adults (ages 18–59). *Ethnicity & Health*, 1-22. https://doi.org/10.1080/13557858.2020.1740174
104. Cage, J., Corley, N. A., & Harris, L. A. (2018). The educational attainment of maltreated youth involved with the child welfare system: Exploring the intersection of race and gender. *Children and Youth Services Review, 88,* 550-557. https://doi.org/10.1016/j.childyouth.2018.04.006
105. Cairney, J., Veldhuizen, S., Vigod, S., Streiner, D. L., Wade, T. J., & Kurdyak, P. (2014). Exploring the social determinants of mental health service use using intersectionality theory and CART analysis. *Journal of Epidemiology and Community Health*, *68*(2), 145–150. https://doi.org/10.1136/jech-2013-203120
106. Calabrese, S. K., Meyer, I. H., Overstreet, N. M., Haile, R., & Hansen, N. B. (2015). Exploring discrimination and mental health disparities faced by Black sexual minority women using a minority stress framework. *Psychology of Women Quarterly*, *39*(3), 287–304. https://doi.org/10.1177/0361684314560730
107. Calderon-Villarreal, A., Mujica, O. J., & Bojorquez, I. (2020). Social inequalities and prevalence of depressive symptoms: A cross-sectional study of women in a Mexican border city, 2014. *Revista Panamericana de Salud Pública, 44,* e9. https://doi.org/10.26633/RPSP.2020.9
108. Calderón Gómez, D. (2019). Technological capital and digital divide among young people: An intersectional approach. *Journal of Youth Studies*, *22*(7), 941–958. https://doi.org/10.1080/13676261.2018.1559283
109. Calzada, E. J., Kim, Y., & O'Gara, J. L. (2019). Skin color as a predictor of mental health in young Latinx children. *Social Science & Medicine, 238,* 112467. https://doi.org/10.1016/j.socscimed.2019.112467
110. Carberry, E. J., & Meyers, J. S. M. (2017). Are the "best" better for everyone? Demographic variation in employee perceptions of Fortune's "Best Companies to Work For." *Equality, Diversity and Inclusion*, *36*(7), 647–669. https://doi.org/10.1108/EDI-01-2017-0017
111. Carlerby, H., Viitasara, E., Knutsson, A., & Gillander Gadin, K. (2011). Subjective health complaints among boys and girls in the Swedish HBSC study: Focussing on parental foreign background. *International Journal of Public Health*, *56*(5), 457–464. https://doi.org/10.1007/s00038-011-0246-8
112. Carliner, H., Delker, E., Fink, D. S., Keyes, K. M., & Hasin, D. S. (2016). Racial discrimination, socioeconomic position, and illicit drug use among US Blacks. *Social*

Psychiatry and Psychiatric Epidemiology, *51*(4), 551–560. https://doi.org/10.1007/s00127-016-1174-y
113. Carrington, P. J. (2016). Gender and age segregation and stratification in criminal collaborations. *Journal of Quantitative Criminology*, *32*(4), 613–649. https://doi.org/10.1007/s10940-015-9269-2
114. Cassan, G. (2019). Affirmative action, education and gender: Evidence from India. *Journal of Development Economics*, *136*, 51–70. https://doi.org/10.1016/j.jdeveco.2018.10.001
115. Castro, J., & Landry, B. (2005). Race, gender, and class variation in the effect of neighborhood violence on adolescent use of violence. *Race, Gender & Class*, *12*(1), 97–120. https://www.jstor.org/stable/41675151
116. Ceccato, V., Näsman, P., & Langefors, L. (2020). Sexual violence on the move: An assessment of youth's victimization in public transportation. *Women & Criminal Justice*, 1-19. https://doi.org/10.1080/08974454.2020.1733732
117. Cech, E. A., & Rothwell, W. R. (2020). LGBT workplace inequality in the federal workforce: Intersectional processes, organizational contexts, and turnover considerations. *ILR Review*, *73*(1), 25–60. https://doi.org/10.1177/0019793919843508
118. Cerezo, A., & Ramirez, A. (2020). Perceived discrimination, alcohol use disorder and alcohol-related problems in sexual minority women of color. *Journal of Social Service Research*, 47(1), 33-46. https://doi.org/10.1080/01488376.2019.1710657
119. Chadwick, C., & DeBlaere, C. (2019). The power of sisterhood: The moderating role of womanism in the discrimination-distress link among women of color in the United States. *Sex Roles*, *81*(5-6), 326–337. https://doi.org/10.1007/s11199-018-0991-8
120. Chakraborty, A., & Mukhopadhayay, S. (2017). Decomposing nutritional inequality by caste and class: A quantitative approach to reckon intersectionality. *Economics Bulletin*, *37*(2), 1339–1350.
121. Chapman, S. J., & Benis, N. (2017). Ceteris non paribus: The intersectionality of gender, race, and region in the gender wage gap. *Women's Studies International Forum*, *65*, 78–86. https://doi.org/10.1016/j.wsif.2017.10.001
122. Chappell, N. L., Dujela, C., & Smith, A. (2015). Caregiver well-being: Intersections of relationship and gender. *Research on Aging*, *37*(6), 623–645. https://doi.org/10.1177/0164027514549258
123. Chesla, C. A., Kwan, C. M. L., Chun, K. M., & Stryker, L. (2014). Gender differences in factors related to diabetes management in Chinese American immigrants. *Western Journal of Nursing Research, 36*(9), 1074–1090. https://doi.org/10.1177/0193945914522718
124. Chiang, S.-Y., Fleming, T., Lucassen, M., Fenaughty, J., Clark, T., & Denny, S. (2017). Mental health status of double minority adolescents: Findings from national cross-sectional health surveys. *Journal of Immigrant and Minority Health, 19*(3), 499–510. https://doi.org/10.1007/s10903-016-0530-z
125. Christensen, M. C., & Harris, R. J. (2019). Correlates of bystander readiness to help among a diverse college student population: An intersectional perspective. *Research in Higher Education*, *60*(8), 1195–1226. https://doi.org/10.1007/s11162-018-09544-6
126. Chua, V., Mathews, M., & Loh, Y. C. (2016). Social capital in Singapore: Gender differences, ethnic hierarchies, and their intersection. *Social Networks*, *47*, 138–150. https://doi.org/10.1016/j.socnet.2016.06.004

127. Cockerham, W. C., Hamby, B. W., Hankivsky, O., Baker, E. H., & Rouhani, S. (2017). Self-rated health and barriers to healthcare in Ukraine: The pivotal role of gender and its intersections. *Communist and Post-Communist Studies*, *50*(1), 53–63. https://doi.org/10.1016/j.postcomstud.2017.01.001
128. Cohen, S. A., Sabik, N. J., Cook, S. K., Azzoli, A. B., & Mendez-Luck, C. A. (2019). Differences within differences: Gender inequalities in caregiving intensity vary by race and ethnicity in informal caregivers. *Journal of Cross-Cultural Gerontology*, *34*(3), 245–263. https://doi.org/10.1007/s10823-019-09381-9
129. Colbert, S. M., & Chan, J. Y. (2020). Effects of sexual orientation, disability, and gender identity on others' prosocial behavior. *Rehabilitation Counseling Bulletin, 64*(1), 52-63. https://doi.org/10.1177/0034355220902240
130. Coleman-Minahan, K. (2017). The socio-political context of migration and reproductive health disparities: The case of early sexual initiation among Mexican-origin immigrant young women. *Social Science and Medicine*, *180*, 85–93. https://doi.org/10.1016/j.socscimed.2017.03.011
131. Colley, L. (2014). Aging public services and the position of older women: An Australian case study. *Journal of Women and Aging*, *26*(2), 160–181. https://doi.org/10.1080/08952841.2014.883232
132. Collier, K. L., Bos, H. M. W., Merry, M. S., & Sandfort, T. G. M. (2013). Gender, ethnicity, religiosity, and same-sex sexual attraction and the acceptance of same-sex sexuality and gender non-conformity. *Sex Roles*, *68*(11-12), 724–737. https://doi.org/10.1007/s11199-012-0135-5
133. Collings, S., Dew, A., Gordon, T., Spencer, M., & Dowse, L. (2018). Intersectional disadvantage: Exploring differences between Aboriginal and non-Aboriginal parents with intellectual disability in the New South Wales child protection system. *Journal of Public Child Welfare*, *12*(2), 170–189. https://doi.org/10.1080/15548732.2017.1379456
134. Collins, T. A., Dumas, T. L., & Moyer, L. P. (2017). Intersecting disadvantages: Race, gender, and age discrimination among attorneys. *Social Science Quarterly*, *98*(5), 1642-1658. https://doi.org/10.1111/ssqu.12376
135. Collins, T.A., & Moyer, L. (2008). Gender, race, and intersectionality on the federal appellate bench. *Political Research Quarterly*, *61*(2), 219–227. https://doi.org/10.1177/1065912907306467
136. Collins, T. W., Grineski, S. E., Chakraborty, J., & McDonald, Y. J. (2011). Understanding environmental health inequalities through comparative intracategorical analysis: Racial/ethnic disparities in cancer risks from air toxics in El Paso County, Texas. *Health and Place*, *17*(1), 335–344. https://doi.org/10.1016/j.healthplace.2010.11.011
137. Conover, K. J., & Israel, T. (2019). Microaggressions and social support among sexual minorities with physical disabilities. *Rehabilitation Psychologoy*, *64*(2), 167–178. https://doi.org/10.org/10.1037/rep0000250
138. Corsbie-Massay, C. L. 'P., Miller, L. C., Christensen, J. L., Appleby, P. R., Godoy, C., & Read, S. J. (2017). Identity conflict and sexual risk for Black and Latino YMSM. *AIDS and Behavior*, *21*(6), 1611–1619. https://doi.org/10.1007/s10461-016-1522-7
139. Coston, B. M. (2020). Patterns of post-traumatic health care service need and access among bisexual and non-monosexual women in the U.S. *Journal of Aggression,*

Maltreatment, & Trauma, *29*(3), 348–364. https://doi.org/10.1080/10926771.2019.1572401
140. Covarrubias, A. (2011). Quantitative intersectionality: A critical race analysis of the Chicana/o educational pipeline. *Journal of Latinos and Education*, *10*(2), 86–105. https://doi.org/10.1080/15348431.2011.556519
141. Covarrubias, A., & Lara, A. (2014). The undocumented (im)migrant educational pipeline: The influence of citizenship status on educational attainment for people of Mexican origin. *Urban Education*, *49*(1), 75–110. https://doi.org/10.1177/0042085912470468
142. Covarrubias, A., & Liou, D. D. (2014). Asian American education and income attainment in the era of post-racial America. *Teachers College Record*, *116*(6), 1-38.
143. Cravens III, R. G. (2018). The politics of queer religion. *Politics & Religion*, *11*(3), 576–623. https://doi.org/http://dx.doi.org/10.1017/S1755048318000056
144. Cummings, J. L. (2020). Assessing U.S. racial and gender differences in happiness, 1972–2016: An intersectional approach. *Journal of Happiness Studies: An Interdisciplinary Forum on Subjective Well-Being*, *21*(2), 709–732. https://doi.org/10.1007/s10902-019-00103-z
145. Cummings, J. L., & Jackson, P. B. (2008). Race, gender, and SES disparities in self-assessed health, 1974-2004. *Research on Aging*, *30*(2), 137–167. https://doi.org/10.1177/0164027507311835
146. Dale, S. K., & Safren, S. A. (2019). Gendered racial microaggressions predict posttraumatic stress disorder symptoms and cognitions among Black women living with HIV. *Psychological Trauma: Theory, Research, Practice, and Policy*, *11*(7), 685-694. https://doi.org/10.1037/tra0000467
147. Daoud, N., Ali Saleh-Darawshy, N., Gao, M., Sergienko, R., Sestito, S. R., & Geraisy, N. (2019). Multiple forms of discrimination and postpartum depression among indigenous Palestinian-Arab, Jewish immigrants and non-immigrant Jewish mothers. *BMC Public Health*, *19*(1) 1-14. https://doi.org/10.1186/s12889-019-8053-x
148. Davis, B. W., & Bowers, A. J. (2019). Examining the career pathways of educators with superintendent certification. *Educational Administration Quarterly*, *55*(1), 3–41. https://doi.org/10.1177/0013161X18785872
149. Davis, S. N., Jacobsen, S. K., & Ryan, M. (2015). Gender, race, and inequality in higher education: An intersectional analysis of faculty-student undergraduate research pairs at a diverse university. *Race, Gender & Class*, *22*(3-4), 7-30. https://www.jstor.org/stable/26505348
150. DeBlaere, C., & Bertsch, K. N. (2013). Perceived sexist events and psychological distress of sexual minority women of color: The moderating role of womanism. *Psychology of Women Quarterly*, *37*(2), 167–178. https://doi.org/10.1177/0361684312470436
151. DeBlaere, C., Brewster, M. E., Bertsch, K. N., DeCarlo, A. L., Kegel, K. A., & Presseau, C. D. (2014). The protective power of collective action for sexual minority women of color: An investigation of multiple discrimination experiences and psychological distress. *Psychology of Women Quarterly*, *38*(1), 20–32. https://doi.org/10.1177/0361684313493252
152. Demant, D., Oviedo-Trespalacios, O., Carroll, J.-A., Ferris, J. A., Maier, L., Barratt, M. J., & Winstock, A. R. (2018). Do people with intersecting identities report more high-

risk alcohol use and lifetime substance use? *International Journal of Public Health*, *63*(5), 621–630. https://doi.org/10.1007/s00038-018-1095-5
153. Denis, A., & Paulos, A. (2011). Ethnicity and the internet use of Barbadian and Franco-Ontarian minority young people. *Journal of Intercultural Studies*, *32*(5), 495–513. https://doi.org/10.1080/07256868.2011.599488
154. De Rosa, E. (2019). Migrant women breadwinners in Italy during the crisis: Improvement or trap? *Journal of Gender Studies*, *28*(3), 288–303. https://doi.org/10.1080/09589236.2018.1441017
155. Dewachter, S., Holvoet, N., & Van Aelst, K. (2018). How does water information flow? Intersectionality in water information networks in a rural Ugandan community. *Water International*, *43*(5), 553–569. https://doi.org/10.1080/02508060.2018.1495047
156. DeWilde, C., Carrington, J., Abbate, A., Burton, C. W., Bearman, G., & Salyer, J. (2019). Structural stress and otherness: How do they influence psychological stress? *Journal of Transcultural Nursing*, *30*(5), 478–491. https://doi.org/10.1177/1043659618823915
157. Dillaway, H., & Broman, C. (2001). Race, class, and gender differences in marital satisfaction and divisions of household labor among dual-earner couples: A case for intersectional analysis. *Journal of Family Issues*, *22*(3), 309–327. https://doi.org/10.1177/019251301022003003
158. Dillbary, J. S., & Edwards, G. (2019). An empirical analysis of sexual orientation discrimination. *The University of Chicago Law Review*, *86*(1), 1–75. https://www.jstor.org/stable/26554392
159. Divney, A. A., Echeverria, S. E., Thorpe, L. E., Trinh-Shevrin, C., & Islam, N. S. (2019). Hypertension prevalence jointly influenced by acculturation and gender in US immigrant groups. *American Journal of Hypertension*, *32*(1), 104–111. https://doi.org/10.1093/ajh/hpy130
160. Dlugonski, D., Martin, T. R., Mailey, E. L., & Pineda, E. (2017). Motives and barriers for physical activity among low-income Black single mothers. *Sex Roles*, *77*(5-6), 379–392. https://doi.org/10.1007/s11199-016-0718-7
161. Donovan, B. (2012). Intersectionality and the substantive representation of migrant interests in Germany. *German Politics and Society*, *30*(4), 23–44. https://doi.org/10.3167/gps.2012.300402
162. Donovan, R. A. (2011). Tough or tender: (Dis)similarities in white college students' perceptions of Black and white women. *Psychology of Women Quarterly*, *35*(3), 458–468. https://doi.org/10.1177/0361684311406874
163. Doren, C., & Lin, K. Y. (2019). Diverging trajectories or parallel pathways? An intersectional and life course approach to the gender earnings gap by race and education. *Socius: Sociological Research for a Dynamic World*, *5*, 2378023119873816. https://doi.org/http://dx.doi.org/10.1177/2378023119873816
164. Dorimé-Williams, M. L., & Giani, M. S. (2019). Impact of race, class, and involvement on collegiate degree attainment. *Journal of College Student Retention: Research, Theory & Practice*, 1521025119885445. https://doi.org/10.1177/1521025119885445
165. Dotson, H., & Carter, J. S. (2012). Changing views toward the death penalty? The intersecting impact of race and gender on attitudes, 1974–2006. *Justice System Journal*, *33*(1), 1–21. https://doi.org/10.1080/0098261X.2012.10767999

166. Douds, A. S., Howard, D., Hummer, D., & Gabbidon, S. L. (2016). Public opinion on the affluenza defense, race, and sentencing decisions: Results from a statewide poll. *Journal of Crime and Justice*, *39*(1), 230-242. https://doi.org/10.1080/0735648X.2015.1108550
167. Drange, I., & Karlsen, H. J. (2016). Simply a matter of being male? Nurses' employment outcomes in the Norwegian labour market. *NORA : Nordic Journal of Women's Studies*, *24*(2), 76–94. https://doi.org/10.1080/08038740.2016.1165732
168. Dubrow, J. K. (2008). How can we account for intersectionality in quantitative analysis of survey data? Empirical illustration for Central and Eastern Europe. *ASK: Spoleczenstwo Badania Metody*, *17*, 85–100.
169. Duffy, M. (2007). Doing the dirty work: Gender, race, and reproductive labor in historical perspective. *Gender and Society*, *21*(3), 313–336. https://doi.org/10.1177/0891243207300764
170. Duncan, D. T., Callander, D., Bowleg, L., Park, S. H., Brinkley-Rubinstein, L., Theall, K. P., & Hickson, D. A. (2020). Intersectional analysis of life stress, incarceration and sexual health risk practices among cisgender Black gay, bisexual and other men who have sex with men in the Deep South of the US: The MARI Study. *Sexual Health*, *17*(1), 38–44. https://doi.org/10.1071/SH19062
171. Dunn, C. E., Hood, K. B., & Owens, B. D. (2019). Loving myself through thick and thin: Appearance contingent self-worth, gendered racial microaggressions and African American women's body appreciation. *Body Image*, *30*, 121–126. https://doi.org/10.1016/j.bodyim.2019.06.003
172. DuPont-Reyes, M. J., Villatoro, A. P., Phelan, J. C., Painter, K., & Link, B. G. (2019). Adolescent views of mental illness stigma: An intersectional lens. *American Journal of Orthopsychiatry*, *90*(2), 201–211. https://doi.org/10.1037/ort0000425
173. Durbin, A., Moineddin, R., Lin, E., Steele, L. S., & Glazier, R. H. (2015). Examining the relationship between neighbourhood deprivation and mental health service use of immigrants in Ontario, Canada: A cross-sectional study. *BMJ Open*, *5*(3) e006690. https://doi.org/10.1136/bmjopen-2014-006690
174. Dyar, C., Feinstein, B. A., Stephens, J., Zimmerman, A. R., Newcomb, M. E., & Whitton, S. W. (2020). Nonmonosexual stress and dimensions of health: Within-group variation by sexual, gender, and racial/ethnic identities. *Psychology of Sexual Orientation and Gender Diversity*, *7*(1), 12–25. https://doi.org/10.1037/sgd0000348
175. Earnshaw, V. A., Rosenthal, L., Gilstad-Hayden, K., Carroll-Scott, A., Kershaw, T. S., Santilli, A., & Ickovics, J. R. (2018). Intersectional experiences of discrimination in a low-resource urban community: An exploratory latent class analysis. *Journal of Community & Applied Social Psychology*, *28*(2), 80–93. https://doi.org/10.1002/casp.2342
176. Earnshaw, V. A., Smith, L. R., Cunningham, C. O., & Copenhaver, M. M. (2015). Intersectionality of internalized HIV stigma and internalized substance use stigma: Implications for depressive symptoms. *Journal of Health Psychology*, *20*(8), 1083–1089. https://doi.org/10.1177/1359105313507964
177. Eeckhaut, M. C. W. (2020). Intersecting inequalities: Education, race/ethnicity, and sterilization. *Journal of Family Issues*, *41*(10), 1905-1929. https://doi.org/10.1177/0192513X19900529

178. Eliason, M. J., Martinson, M., & Carabez, R. M. (2015). Disability among sexual minority women: Descriptive data from an invisible population. *LGBT Health*, *2*(2), 113–120. https://doi.org/http://dx.doi.org/10.1089/lgbt.2014.0091
179. Elmeroth, E. (2012). Winners and losers in the Swedish school system: An intersectional perspective. *Race, Gender & Class*, *19*(3/4), 113–129. https://www.jstor.org/stable/43497491
180. Elu, J. U., & Loubert, L. (2013). Earnings inequality and the intersectionality of gender and ethnicity in Sub-Saharan Africa: The case of Tanzanian manufacturing. *American Economic Review*, *103*(3), 289–292.
181. English, D., Carter, J. A., Forbes, N., Bowleg, L., Malebranche, D. J., Talan, A. J., & Rendina, H. J. (2020). Intersectional discrimination, positive feelings, and health indicators among Black sexual minority men. *Health Psychology*, *39*(3), 220–229. https://doi.org/10.1037/hea0000837
182. English, D., Rendina, H. J., & Parsons, J. T. (2018). The effects of intersecting stigma: A longitudinal examination of minority stress, mental health, and substance use among Black, Latino, and multiracial gay and bisexual men. *Psychology of Violence*, *8*(6), 669–679. https://doi.org/10.1037/vio0000218
183. Escobar, K. M., Sivaram, M., & Gorey, K. M. (2019). Multiplicative advantages of hispanic men living in hispanic enclaves: Intersectionality in colon cancer care: A research note. *Journal of Sociology & Social Welfare*, *46*(1), 37–48.
184. Etherington, N. (2015). Race, gender, and the resources that matter: An investigation of intersectionality and health. *Women and Health*, *55*(7), 754–777. https://doi.org/10.1080/03630242.2015.1050544
185. Evans, C. R., & Erickson, N. (2019). Intersectionality and depression in adolescence and early adulthood: A MAIHDA analysis of the national longitudinal study of adolescent to adult health, 1995–2008. *Social Science & Medicine*, *220*, 1–11. https://doi.org/10.1016/j.socscimed.2018.10.019
186. Everett, B. G., Steele, S. M., Matthews, A. K., & Hughes, T. L. (2019). Gender, race, and minority stress among sexual minority women: An intersectional approach. *Archives of Sexual Behavior*, *48*(5), 1505–1517. https://doi.org/10.1007/s10508-019-1421-x
187. Fattoracci, E. S. M., Revels-Macalinao, M., & Huynh, Q.-L. (2020). Greater than the sum of racism and heterosexism: Intersectional microaggressions toward racial/ethnic and sexual minority group members. *Cultural Diversity and Ethnic Minority Psychology*. https://doi.org/10.1037/cdp0000329
188. Fedock, G., Cummings, C., Kubiak, S., Bybee, D., Campbell, R., & Darcy, K. (2019). Incarcerated women's experiences of staff-perpetrated rape: Racial disparities and justice gaps in institutional responses. *Journal of Interperonal Violence*, 0886260519850531. https://doi.org/10.1177/0886260519850531
189. Ferlatte, O., Salway, T., Hankivsky, O., Trussler, T., Oliffe, J. L., & Marchand, R. (2018). Recent suicide attempts across multiple social identities among gay and bisexual men: An intersectionality analysis. *Journal of Homosexuality*, *65*(11), 1507–1526. https://doi.org/10.1080/00918369.2017.1377489
190. Ferlatte, O., Salway, T., Rice, S. M., Oliffe, J. L., Knight, R., & Ogrodniczuk, J. S. (2019). Inequities in depression within a population of sexual and gender minorities.

Journal of Mental Health, *29*(5), 573-580. https://doi.org/10.1080/09638237.2019.1581345

191. Ferlatte, O., Salway, T., Trussler, T., Oliffe, J. L., & Gilbert, M. (2018). Combining intersectionality and syndemic theory to advance understandings of health inequities among Canadian gay, bisexual and other men who have sex with men. *Critical Public Health*, *28*(5), 509–521. https://doi.org/10.1080/09581596.2017.1380298
192. Fernandez-Lozano, I. (2018). If you dare to ask: Self-perceived possibilities of Spanish fathers to reduce work hours. *Community, Work & Family*, *21*(4), 482–498. https://doi.org/10.1080/13668803.2017.1365692
193. Fisher, A. R. M., Oddsson, G., & Wada, T. (2013). Policing class and race in urban America. *International Journal of Sociology and Social Policy*, *33*(5), 309–327. https://doi.org/10.1108/IJSSP-09-2012-0085
194. Fisher, S., Middleton, K., Ricks, E., Malone, C., Briggs, C., & Barnes, J. (2015). Not just Black and white: Peer victimization and the intersectionality of school diversity and race. *Journal of Youth and Adolescence*, *44*(6), 1241–1250. https://doi.org/10.1007/s10964-014-0243-3
195. Fisk, S. A., Mulinari, S., Wemrell, M., Leckie, G., Perez Vicente, R., & Merlo, J. (2018). Chronic obstructive pulmonary disease in Sweden: An intersectional multilevel analysis of individual heterogeneity and discriminatory accuracy. *SSM Population Health*, *4*, 334–346. https://doi.org/10.1016/j.ssmph.2018.03.005
196. Fitzsimmons, S. R., Baggs, J., & Brannen, M. Y. (2020). Intersectional arithmetic: How gender, race and mother tongue combine to impact immigrants' work outcomes. *Journal of World Business*, *55*(1), 101013. https://doi.org/10.1016/j.jwb.2019.101013
197. Flippen, C. A. (2014). Intersectionality at work: Determinants of labor supply among immigrant Latinas. *Gender and Society*, *28*(3), 404–434. https://doi.org/10.1177/0891243213504032
198. Florian, S. M. (2018). Motherhood and employment among whites, Hispanics, and Blacks: A life course approach. *Journal of Marriage and Family*, *80*(1), 134–149. https://doi.org/10.1111/jomf.12448
199. Folke, O., Freidenvall, L., & Rickne, J. (2015). Gender quotas and ethnic minority representation: Swedish evidence from a longitudinal mixed methods study. *Politics and Gender*, *11*(2), 345–381. https://doi.org/10.1017/S1743923X15000069
200. Foster, C. H. (2008). The welfare queen: Race, gender, class, and public opinion. *Race, Gender & Class*, *15*(3-4), 162–179. https://www.jstor.org/stable/41674659
201. Foster, H. (2011). The influence of incarceration on children at the intersection of parental gender and race/ethnicity: A focus on child living arrangements. *Journal of Ethnicity in Criminal Justice*, *9*(1), 1–21. https://doi.org/http://dx.doi.org/10.1080/15377938.2011.535457
202. Frasure-Yokley, L. (2018). Choosing the velvet glove: Women voters, ambivalent sexism, and vote choice in 2016. *Journal or Race, Ethnicity, and Politics*, *3*(1), 3–25. https://doi.org/10.1017/rep.2017.35
203. Friedman, C., & Leaper, C. (2010). Sexual-minority college women's experiences with discrimination: Relations with identity and collective action. *Psychology of Women Quarterly*, *34*(2), 152–164. https://doi.org/10.1111/j.1471-6402.2010.01558.x

204. Fuller, E., Hollingworth, L., & An, B. P. (2019). Exploring intersectionality and the employment of school leaders. *Journal of Educational Administration*, *57*(2), 134–151. https://doi.org/10.1108/JEA-07-2018-0133
205. Gagné, T., & Veenstra, G. (2017). Inequalities in hypertension and diabetes in Canada: Intersections between racial identity, gender, and income. *Ethnicity & Disease*, *27*(4), 371–378. https://doi.org/10.18865/ed.27.4.371
206. Galupo, M. P. (2009). Cross-category friendship patterns: Comparison of heterosexual and sexual minority adults. *Journal of Social and Personal Relationships*, *26*(6-7), 811–831. https://doi.org/10.1177/0265407509345651
207. Galupo, M. P., & Gonzalez, K. A. (2013). Friendship values and cross-category friendships: Understanding adult friendship patterns across gender, sexual orientation and race. *Sex Roles*, *68*(11-12), 779–790. https://doi.org/10.1007/s11199-012-0211-x
208. Galupo, M. P., Mitchell, R. C., & Davis, K. S. (2015). Sexual minority self-identification: Multiple identities and complexity. *Psychology of Sexual Orientation and Gender Diversity*, *2*(4), 355–364. https://doi.org/http://dx.doi.org/10.1037/sgd0000131
209. Galvan, F. H., Chen, Y.-T., Contreras, R., & O'Connell, B. (2019). Violence inflicted on Latina transgender women living with HIV: Rates and associated factors by perpetrator type. *AIDS and Behavior*, 1-11. https://doi.org/10.1007/s10461-019-02751-x
210. Garnett, B. R., Masyn, K. E., Austin, S. B., Miller, M., Williams, D. R., & Viswanath, K. (2014). The intersectionality of discrimination attributes and bullying among youth: An applied latent class analysis. *Journal of Youth and Adolescence*, *43*(8), 1225–1239. https://doi.org/10.1007/s10964-013-0073-8
211. Gatica-Domínguez, G., Victora, C., & Barros, A. J. D. (2019). Ethnic inequalities and trends in stunting prevalence among Guatemalan children: An analysis using national health surveys 1995-2014. *International Journal of Equity in Health*, *18*(1), 110. https://doi.org/10.1186/s12939-019-1016-0
212. Gattamorta, K. A., Salerno, J. P., & Castro, A. J. (2019). Intersectionality and health behaviors among US high school students: Examining race/ethnicity, sexual identity, and sex. *Journal of School Health*, *89*(10), 800–808. https://doi.org/10.1111/josh.12817
213. Gaughan, M., Melkers, J., & Welch, E. (2018). Differential social network effects on scholarly productivity: An intersectional analysis. *Science, Technology, & Human Values*, *43*(3), 570–599. https://doi.org/10.1177/0162243917735900
214. Gazard, B., Chui, Z., Harber-Aschan, L., MacCrimmon, S., Bakolis, I., Rimes, K., Hotopf, M., & Hatch, S. L. (2018). Barrier or stressor? The role of discrimination experiences in health service use. *BMC Public Health*, *18*(1) 1-14. https://doi.org/10.1186/s12889-018-6267-y
215. Gazard, B., Frissa, S., Nellums, L., Hotopf, M., & Hatch, S. L. (2015). Challenges in researching migration status, health and health service use: An intersectional analysis of a South London community. *Ethnicity and Health*, *20*(6), 564–593. https://doi.org/10.1080/13557858.2014.961410
216. Ghosh, S., Choi, W. Y., Williams, A., Duggleby, W., Ploeg, J., Markle-Reid, M., & Peacock, S. (2019). Burden on caregivers of adults with multiple chronic conditions: Intersectionality of age, gender, education level, employment status, and impact on

social life. *Canadian Journal on Aging/La Revue canadienne du vieillissement, 39*(3), 456-467. https://doi.org/10.1017/S071498081900045X
217. Gilliard-Matthews, S. (2017). Intersectional race effects on citizen-reported traffic ticket decisions by police in 1999 and 2008. *Race Justice*, *7*(4), 299–324. https://doi.org/10.1177/2153368716648002
218. Glass, J. E., Rathouz, P. J., Gattis, M., Joo, Y. S., Nelson, J. C., & Williams, E. C. (2017). Intersections of poverty, race/ethnicity, and sex: Alcohol consumption and adverse outcomes in the United States. In *Social Psychiatry and Psychiatric Epidemiology 52*(5), 515-524. https://doi.org/10.1007/s00127-017-1362-4
219. Godbolt, D., Vaghela, P., Burdette, A. M., & Hill, T. D. (2018). Religious attendance and body mass: An examination of variations by race and gender. *Journal of Religion and Health*, *57*(6), 2140–2152. https://doi.org/10.1007/s10943-017-0490-1
220. Godwin, A., Klotz, L., Hazari, Z., & Potvin, G. (2016). Sustainability goals of students underrepresented in engineering: An intersectional study. *International Journal of Engineering Education*, *32*(4), 1742–1748.
221. Goldenberg, T., Jadwin-Cakmak, L., Popoff, E., Reisner, S. L., Campbell, B. A., & Harper, G. W. (2019). Stigma, gender affirmation, and primary healthcare use among Black transgender youth. *Journal of Adolescent Health*, *65*(4), 483–490. https://doi.org/10.1016/j.jadohealth.2019.04.029
222. Goldstein, N. D., Burstyn, I., LeVasseur, M. T., & Welles, S. L. (2016). Drug use among men by sexual behaviour, race and ethnicity: Prevalence estimates from a nationally representative US sample. *International Journal of Drug Policy*, *36*, 148–150. https://doi.org/http://dx.doi.org/10.1016/j.drugpo.2016.01.008
223. Gonçalves, M., & Matos, M. (2020). Interpersonal violence in immigrant women in Portugal: An intersectional approach. *Journal of Immigrant & Refugee Studies*, *18*(1), 22–41. https://doi.org/10.1080/15562948.2018.1557312
224. Gonzales, G., Dedania, R., & Driscoll, R. (2019). Health insurance coverage and access to care among US-born and foreign-born sexual minorities. *Journal of Immigrant and Minority Health*, *21*(3), 540–548. https://doi.org/10.1007/s10903-018-0774-x
225. Gonzales, G., & Ortiz, K. (2015). Health insurance disparities among racial/ethnic minorities in same-sex relationships: An intersectional approach. *American Journal of Public Health*, *105*(6), 1106–1113. https://doi.org/10.2105/AJPH.2014.302459
226. Goodwin, L., Gazard, B., Aschan, L., MacCrimmon, S., Hotopf, M., & Hatch, S. L. (2018). Taking an intersectional approach to define latent classes of socioeconomic status, ethnicity and migration status for psychiatric epidemiological research. *Epidemiology and Psychiatric Sciences*, *27*(6), 589–600. https://doi.org/10.1017/S2045796017000142
227. Gorman, B. K., Altman, C. E., Guerra, R., & Chavez, S. (2020). Gender, education, and physical health among adults in central mexico. *Sociological Perspectives*, *63*(2), 181–200. https://doi.org/10.1177/0731121419836964
228. Gorman, B. K., Denney, J. T., Dowdy, H., & Medeiros, R. A. (2015). A new piece of the puzzle: Sexual orientation, gender, and physical health status. *Demography*, *52*(4), 1357–1382. https://doi.org/10.1007/s13524-015-0406-1
229. Graham, L. M., Lanier, P., Finno-Velasquez, M., & Johnson-Motoyama, M. (2018). Substantiated reports of sexual abuse among Latinx children: Multilevel models of

national data. *Journal of Family Violence*, *33*(7), 481–490. https://doi.org/10.1007/s10896-018-9967-2
230. Greaves, L. M., Barlow, F. K., Huang, Y., Stronge, S., & Sibley, C. G. (2017). Personality across sexual identity and gender in a national probability sample in New Zealand. *Sex Roles*, *77*(9), 653-662. https://doi.org/10.1007/s11199-017-0752-0
231. Green, B. N. (2019). Strong like my mama: The legacy of "strength," depression, and suicidality in African American women. *Women & Therapy*, *42*(3-4), 265–288. https://doi.org/10.1080/02703149.2019.1622909
232. Greene, M. Z., Hughes, T. L., Hanlon, A., Huang, L., Sommers, M. S., & Meghani, S. H. (2019). Predicting cervical cancer screening among sexual minority women using classification and regression tree analysis. *Preventive Medicine Reports*, *13*, 153–159. https://doi.org/10.1016/j.pmedr.2018.11.007
233. Gregus, S. J., Stevens, K. T., Seivert, N. P., Tucker, R. P., & Callahan, J. L. (2019). Student perceptions of multicultural training and program climate in clinical psychology doctoral programs. *Training and Education in Professional Psychology*, *14*(4), 293–307. https://doi.org/10.1037/tep0000289
234. Griffin, B. J., Worthington Jr., E. L., Leach, J. D., Hook, J. N., Grubbs, J., Exline, J. J., & Davis, D. E. (2016). Sexual congruence moderates the associations of hypersexual behavior with spiritual struggle and sexual self-concept. *Sexual Addiction and Compulsivity*, *23*(2-3), 279–295. https://doi.org/10.1080/10720162.2016.1150924
235. Grollman, E. A. (2012). Multiple forms of perceived discrimination and health among adolescents and young adults. *Journal of Health and Social Behavior*, *53*(2), 199–214. https://doi.org/10.1177/0022146512444289
236. Grollman, E. A. (2018). Sexual orientation differences in whites' racial attitudes. *Socioogical Forum*, *33*(1), 186–210. https://doi.org/10.1111/socf.12405
237. Grollman, E. A. (2019). Americans' gender attitudes at the intersection of sexual orientation and gender. *Journal of Homosexuality*, *66*(2), 141–172. https://doi.org/10.1080/00918369.2017.1398022
238. Guenther, E. A., & Koeszegi, S. T. (2017). Intertwined effects of gender and migration status on persistence in SET study programmes. *European Journal of Engineering Education*, *42*(6), 890–915. https://doi.org/10.1080/03043797.2016.1228613
239. Guittar, N. A., & Pals, H. (2014). Intersecting gender with race and religiosity: Do unique social categories explain attitudes toward homosexuality? *Current Sociology*, *62*(1), 41–62. https://doi.org/10.1177/0011392113516985
240. Gupta, N. (2019). Intersectionality of gender and caste in academic performance: Quantitative study of an elite Indian engineering institute. *Gender, Technology and Development*, *23*(2), 165–186. https://doi.org/10.1080/09718524.2019.1636568
241. Gwartney, P. A. (2007). Race, gender, class, and perceptions of terrorism in the immediate aftermath of September 11, 2001. *Race, Gender & Class*, *14*(3-4), 77–97. https://www.jstor.org/stable/41675291
242. Haeberer, M., León-Gómez, I., Pérez-Gómez, B., Tellez-Plaza, M., Rodríguez-Artalejo, F., & Galán, I. (2020). Social inequalities in cardiovascular mortality in Spain from an intersectional perspective. *Revista Española de Cardiología*, *73*(4), 282–289. https://doi.org/10.1016/j.recesp.2019.07.007
243. Haile, R., Rowell-Cunsolo, T. L., Parker, E. A., Padilla, M. B., & Hansen, N. B. (2014). An empirical test of racial/ethnic differences in perceived racism and affiliation with

the gay community: Implications for HIV risk. *Journal of Social Issues*, *70*(2), 342–359. https://doi.org/10.1111/josi.12063
244. Halim, N., Yount, K. M., & Cunningham, S. (2013). Do scheduled caste and scheduled tribe women legislators mean lower gender-caste gaps in primary schooling in India? *Social Science Research*, *58*, 122-134. https://doi.org/10.1016/j.ssresearch.2016.01.002
245. Halim, N., Yount, K. M., Cunningham, S. A., & Pande, R. P. (2016). Women's political empowerment and investments in primary schooling in India. *Social Indicators Research*, *125*(3), 813–851. https://doi.org/10.1007/s11205-015-0870-4
246. Hamidullah, M. F., & Riccucci, N. M. (2017). Intersectionality and family-friendly policies in the federal government: Perceptions of women of color. *Administration and Society*, *49*(1), 105–120. https://doi.org/10.1177/0095399715623314
247. Hansson, L., & Aavik, K. (2012). The effect of gender and ethnicity and their intersection on work satisfaction and earnings in Estonia, 1993-2008. *Studies of Transition States and Societies*, *4*(2), 3–19.
248. Harackiewicz, J. M., Canning, E. A., Tibbetts, Y., Priniski, S. J., & Hyde, J. S. (2016). Closing achievement gaps with a utility-value intervention: Disentangling race and social class. *Journal of Personality and Social Psychology*, *111*(5), 745–765. https://doi.org/10.1037/pspp0000075
249. Harell, A. (2017). Intersectionality and gendered political behaviour in a multicultural Canada. *Canadian Journal of Political Science*, *50*(2), 495–514. https://doi.org/10.1017/S000842391700021X
250. Hargrove, T. W. (2018a). BMI trajectories in adulthood: The intersection of skin color, gender, and age among African Americans. *Journal of Health and Social Behavior*, *59*(4), 501–519. https://doi.org/10.1177/0022146518802439
251. Hargrove, T. W. (2018b). Intersecting social inequalities and body mass index trajectories from adolescence to early adulthood. *Journal of Health and Social Behavior*, *59*(1), 56–73. https://doi.org/10.1177/0022146517746672
252. Hargrove, T. W., Halpern, C. T., Gaydosh, L., Hussey, J. M., Whitsel, E. A., Dole, N., Hummer, R. A., & Harris, K. M. (2020). Race/ethnicity, gender, and trajectories of depressive symptoms across early- and mid-life among the Add Health Cohort. *Journal of Racial and Ethnic Health Disparities*, *7*(4), 619-629. https://doi.org/10.1007/s40615-019-00692-8
253. Harnois, C. E. (2005). Different paths to different feminisms?: Bridging multiracial feminist theory and quantitative sociological gender research. *Gender & Society*, *19*(6), 809–828. https://doi.org/10.1177/0891243205280026
254. Harnois, C. E. (2010). Race, gender, and the Black women's standpoint. *Sociological Forum*, *25*(1), 68–85. https://doi.org/10.1111/j.1573-7861.2009.01157.x
255. Harnois, C. E. (2014). Are perceptions of discrimination unidimensional, oppositional, or intersectional? Examining the relationship among perceived racial-ethnic-, gender-, and age-based discrimination. *Sociological Perspectives*, *57*(4), 470–487. https://doi.org/10.1177/0731121414543028
256. Harnois, C. E. (2015a). Jeopardy, consciousness, and multiple discrimination: intersecting inequalities in contemporary Western Europe. *Sociological Forum*, *30*(4), 971–994. https://doi.org/10.1111/socf.12204

257. Harnois, C. E. (2015b). Race, ethnicity, sexuality, and women's political consciousness of gender. *Social Psychology Quarterly*, *78*(4), 365–386. https://doi.org/10.1177/0190272515607844
258. Harnois, C. E. (2017). Intersectional masculinities and gendered political consciousness: How do race, ethnicity and sexuality shape men's awareness of gender inequality and support for gender activism? *Sex Roles*, *77*(3-4), 141–154. https://doi.org/10.1007/s11199-016-0702-2
259. Harnois, C. E., & Bastos, J. L. (2018). Discrimination, harassment, and gendered health inequalities: Do perceptions of workplace mistreatment contribute to the gender gap in self-reported health? *Journal of Health and Social Behavior*, *59*(2), 283–299. https://doi.org/10.1177/0022146518767407
260. Harnois, C. E., & Ifatunji, M. (2011). Gendered measures, gendered models: Toward an intersectional analysis of interpersonal racial discrimination. *Ethnic and Racial Studies*, *34*(6), 1006–1028. https://doi.or/10.1080/01419870.2010.516836
261. Harr, B. E., & Kane, E. W. (2008). Intersectionality and queer student support for queer politics. *Race, Gender & Class*, *15*(3-4), 283–299. https://www.jstor.org/stable/41674665
262. Harris, J., & Kruger, A. C. (2019). Exploring the influence of racial-ethnic and gender identity on the prosocial behaviors of African American adolescent males. *Youth & Society*, *53*(3), 512-535. https://doi.org/10.1177/0044118X19854936
263. Harris, L., Kleiber, D., Goldin, J., Darkwah, A., & Morinville, C. (2017). Intersections of gender and water: Comparative approaches to everyday gendered negotiations of water access in underserved areas of Accra, Ghana and Cape Town, South Africa. *Journal of Gender Studies*, *26*(5), 561–582. https://doi.org/10.1080/09589236.2016.1150819
264. Hatchel, T., & Marx, R. (2018). Understanding intersectionality and resiliency among transgender adolescents: Exploring pathways among peer victimization, school belonging, and drug use. *International Journal of Environmental Research and Public Health*, *15(6)*, 1289. https://doi.org/10.3390/ijerph15061289
265. Hatchel, T., Valido, A., De Pedro, K. T., Huang, Y., & Espelage, D. L. (2019). Minority stress among transgender adolescents: The role of peer victimization, school belonging, and ethnicity. *Journal of Child and Family Studies*, *28*(9), 2467–2476. https://doi.org/10.1007/s10826-018-1168-3
266. Haukenes, I., Löve, J., Hensing, G., Knudsen, A. K., Øverland, S., Vahtera, J., Sivertsen, B., Tell, G. S., & Skogen, J. C. (2019). Inequity in disability pension: An intersectional analysis of the co-constitution of gender, education and age. The Hordaland Health Study. *Critical Public Health*, *29*(3), 302–313. https://doi.org/10.1080/09581596.2018.1469730
267. Henry, D. A., Cortés, L. B., & Votruba-Drzal, E. (2020). Black-white achievement gaps differ by family socioeconomic status from early childhood through early adolescence. *Journal of Educational Psychology*, *112*(8), 1471–1489. https://doi.org/10.1037/edu0000439
268. Hernández-Yumar, A., Wemrell, M., Alessón, I. A., López-Valcárcel, B. G., Leckie, G., & Merlo, J. (2018). Socioeconomic differences in body mass index in Spain: An intersectional multilevel analysis of individual heterogeneity and discriminatory accuracy. *PLoS ONE*, *13*(12). https://doi.org/10.1371/journal.pone.0208624

269. Hesse-Biber, S., & An, C. (2017). Within-gender differences in medical decision making among male carriers of the BRCA genetic mutation for hereditary breast cancer. *American Journal of Men's Health*, *11*(5), 1444–1459. https://doi.org/10.1177/1557988315610806
270. Himmelstein, M. S., Puhl, R. M., & Quinn, D. M. (2017). Intersectionality: An understudied rramework for addressing weight stigma. *American Journal of Preventive Medicine*, *53*(4), 421–431. https://doi.org/10.1016/j.amepre.2017.04.003
271. Hinze, S. W., Lin, J., & Andersson, T. E. (2012). Can we capture the intersections? Older black women, education, and health. *Women's Health Issues*, *22*(1), e91–e98. https://doi.org/10.1016/j.whi.2011.08.002
272. Holland, M. M. (2020). Fast-tracking justice: An examination of the role of gender in the application of federal early disposition decisions and other departure outcomes for immigration offenses. *Women & Criminal Justice*, *30*(2), 126–146. https://doi.org/10.1080/08974454.2019.1664968
273. Holley, L. C., Mendoza, N. S., Del-Colle, M. M., & Bernard, M. L. (2016). Heterosexism, racism, and mental illness discrimination: Experiences of people with mental health conditions and their families. *Journal of Gay and Lesbian Social Services*, *28*(2), 93–116. https://doi.org/10.1080/10538720.2016.1155520
274. Holmsten, S S, Moser, R. G., & Slosar, M. C. (2010). Do ethnic parties exclude women? *Comparative Political Studies*, *43*(10), 1179–1201. https://doi.org/10.1177/0010414009347831
275. Homewood, K., Nielsen, M. R., & Keane, A. (2020). Women, wellbeing and wildlife management areas in Tanzania. *The Journal of Peasant Studies*, 1-28. https://doi.org/10.1080/03066150.2020.1726323
276. Hsieh, N., & Ruther, M. (2016). Sexual minority health and health risk factors: Intersection effects of gender, race, and sexual identity. *American Journal of Preventive Medicine*, *50*(6), 746–755. https://doi.org/10.1016/j.amepre.2015.11.016
277. Hughes, A. E., Tiro, J. A., Balasubramanian, B. A., Skinner, C. S., & Pruitt, S. L. (2018). Social disadvantage, healthcare utilization, and colorectal cancer screening: Leveraging longitudinal patient address and health records data. *Cancer Epidemiology, Biomarkers & Prevention*, *27*(12), 1424–1432. https://doi.org/10.1158/1055-9965.EPI-18-0446
278. Hughes, M. M. (2011). Intersectionality, quotas, and minority women's political representation worldwide. *American Political Science Review*, *105*(3), 604–620. https://doi.org/10.1017/S0003055411000293
279. Hum, D., & Simpson, W. (2003). Labour market training of new Canadians and limitations to the intersectionality framework. *Canadian Ethnic Studies/Etudes Ethniques Au Canada*, *35*(3), 56–69.
280. Huynh, P. T. A., & Resurreccion, B. P. (2014). Women's differentiated vulnerability and adaptations to climate-related agricultural water scarcity in rural Central Vietnam. *Climate and Development*, *6*(3), 226–237. https://doi.org/10.1080/17565529.2014.886989
281. Ibrahim, H., & Johnson Jr, O. (2020). School discipline, race–gender and STEM readiness: A hierarchical analysis of the impact of school discipline on math achievement in high school. *The Urban Reviewer*, *52*(1), 75–99. https://doi.org/10.1007/s11256-019-00513-6

282. Ikeler, P., & Limonic, L. (2018). Middle class decline? The growth of professional-managers in the neoliberal era. *The Sociological Quarterly*, *59*(4), 549–570. https://doi.org/10.1080/00380253.2018.1479197
283. Ilunga, B. B., Eales, O. O., Marcus, T. S., Smith, S., & Hugo, J. F. (2020). Interpreting mamelodi community-oriented primary care data on tuberculosis loss to follow-up through the lens of intersectionality. *African Journal of Primary Health Care & Family Medicine*, *12*(1), 1–6. https://doi.org/10.4102/phcfm.v12i1.2081
284. Imdorf, C., Koomen, M., Murdoch, J., & Guegnard, C. (2017). Do vocational pathways improve higher education access for women and men from less privileged social backgrounds? A comparison of vocational tracks to higher education in France and Switzerland. *Rassegna Italiana di Sociologia*, *58*(2), 283–314. https://doi.org/10.1423/87310
285. Irizarry, Y., May, D. C., Davis, A., & Wood, P. B. (2016). Mass incarceration through a different lens. *Race and Justice*, *6*(3), 236–256. https://doi.org/10.1177/2153368715603103
286. Jackman, K., Kreuze, E. J., Caceres, B. A., & Schnall, R. (2020). Bullying and peer victimization of minority youth: Intersections of sexual identity and race/ethnicity. *Journal of School Health*, *90*(5), 368–377. https://doi.org/10.1111/josh.12883
287. Jackson, M. C., Galvez, G., Landa, I., Buonora, P., & Thoman, D. B. (2016). Science that matters: The importance of a cultural connection in underrepresented students' science pursuit. *CBE Life Sciences Education*, *15*(3), ar42. https://doi.org/10.1187/cbe.16-01-0067
288. Jackson, S. D., Mohr, J. J., Sarno, E. L., Kindahl, A. M., & Jones, I. L. (2020). Intersectional experiences, stigma-related stress, and psychological health among Black LGBQ individuals. *Journal of Consulting and Clinical Psychology*, *88*(5), 416–428. https://doi.org/10.1037/ccp0000489
289. Jang, S., & Kim, J. (2018). Remedying food policy invisibility with spatial intersectionality: A case study in the detroit metropolitan area. *Journal of Public Policy & Marketing*, *37*(1), 167–187. https://doi.org/10.1509/jppm.16.194
290. Jang, S. T. (2018). The implications of intersectionality on Southeast Asian female students' educational outcomes in the United States: A critical quantitative intersectionality analysis. *American Educational Research Journal*, *55*(6), 1268–1306. https://doi.org/10.3102/0002831218777225
291. Jang, S. T. (2019). Schooling experiences and educational outcomes of Latinx secondary school students living at the intersections of multiple social constructs. *Urban Education*, 0042085919857793. https://doi.org/10.1177/0042085919857793
292. Jefferson, K., Neilands, T. B., & Sevelius, J. (2013). Transgender women of color: Discrimination and depression symptoms. *Ethnicity and Inequalities in Health and Social Care*, *6*(4), 121–136. https://doi.org/10.1108/EIHSC-08-2013-0013
293. Jensenius, F. R. (2016). Competing inequalities? On the intersection of gender and ethnicity in candidate nominations in Indian elections. *Government and Opposition*, *51*(3), 440–463. https://doi.org/10.1017/gov.2016.8
294. Jerald, M. C., Cole, E. R., Ward, L. M., & Avery, L. R. (2017). Controlling images: How awareness of group stereotypes affects Black women's well-being. *Journal of Counseling Psychology*, *64*(5), 487–499. https://doi.org/10.1037/cou0000233

295. Joe, W. (2013). Health inequalities, level differentials and progress assessment: Measles vaccination coverage in India. *Artha Vijñana*, *55*(1), 56–70.
296. Joe, W. (2015a). Distressed financing of household out-of-pocket health care payments in India: Incidence and correlates. *Health Policy and Planning*, *30*(6), 728–741. https://doi.org/10.1093/heapol/czu050
297. Joe, W. (2015b). Intersectional inequalities in immunization in India, 1992-93 to 2005-06: A progress assessment. *Health Policy and Planning*, *30*(4), 407–422. https://doi.org/10.1093/heapol/czu023
298. Johnson, L., Slayter, E., & Livingstone, A. (2020). Locating the intersections of disability, race and ethnicity in adoption rates among foster children introduction. *Adoption Quarterly*, *23*(2), 110–134. https://doi.org/10.1080/10926755.2020.1719252
299. Jones, A. (2015). For Black models scroll down: Webcam modeling and the racialization of erotic labor. *Sexuality and Culture*, *19*(4), 776–799. https://doi.org/10.1007/s12119-015-9291-4
300. Jones, A. M., Finkelstein, R., & Koehoorn, M. (2018). Disability and workplace harassment and discrimination among Canadian federal public service employees. *Canadian Journal of Public Health*, *109*(1), 79–88. https://doi.org/10.17269/s41997-018-0022-0
301. Joseph, J. (2006). Drug offenses, gender, ethnicity, and nationality: Women in prison in England and Wales. *The Prison Journal*, *86*(1), 140–157. https://doi.org/10.1177/0032885505283926
302. Juhasz Liebermann, A., Suter, C., & Iglesias Rutishauser, K. (2014). Segregation or integration? Immigrant self-employment in Switzerland. *Journal of International Migration and Integration*, *15*(1), 93–115. https://doi.org/10.1007/s12134-012-0270-4
303. Kanchi, R., Perlman, S. E., Chernov, C., Wu, W., Tabaei, B. P., Trinh-Shevrin, C., Islam, N., Seixas, A., Rodriguez-Lopez, J., & Thorpe, L. E. (2018). Gender and race disparities in cardiovascular disease risk factors among New York City adults: New York City Health and Nutrition Examination Survey (NYC HANES) 2013–2014. *Urban Health*, *95*(6), 801–812. https://doi.org/10.1007/s11524-018-0287-x
304. Kattari, S. K., Atteberry-Ash, B., Eugene Walls, N., Rusow, J., Klemmer, C., & Kattari, L. (2019). Differential sexual behavior experiences of LGBQ and transgender/nonbinary young people in Colorado. *Youth & Society*, *53*(3), 371-391. https://doi.org/10.1177/0044118X19854783
305. Kattari, S. K., Bakko, M., Langenderfer-Magruder, L., & Holloway, B. T. (2020). Transgender and nonbinary experiences of victimization in health care. *Journal of Interpersonal Violence*, 0886260520905091. https://doi.org/10.1177/0886260520905091
306. Kattari, S. K., Walls, N. E., & Speer, S. R. (2017). Differences in experiences of discrimination in accessing social services among transgender/gender nonconforming individuals by (dis)ability. *Journal of Social Work in Disability & Rehabilitation*, *16*(2), 116–140. https://doi.org/10.1080/1536710X.2017.1299661
307. Kavanaugh, S. A., Taylor, A. B., Stuhlsatz, G. L., Neppl, T. K., & Lohman, B. J. (2020). Family and community support among sexual minorities of color: The role of sexual minority identity prominence and outness on psychological well-being. *Journal of GLBT Family Studies*, *16*(1), 1–17. https://doi.org/10.1080/1550428X.2019.1593279

308. Kelly, M, & Gauchat, G. (2016). Feminist identity, feminist politics: U.S. feminists' attitudes toward social policies. *Sociological Perspectives*, *59*(4), 855–872. https://doi.org/10.1177/0731121415594281
309. Kennedy, A. C., Bybee, D., Kulkarni, S. J., & Archer, G. (2012). Sexual victimization and family violence among urban African American adolescent women: Do violence cluster profiles predict partner violence victimization and sex trade exposure? *Violence Against Women*, *18*(11), 1319–1338. https://doi.org/10.1177/1077801212470544
310. Kershaw, P., & Forer, B. (2010). Selection of area-level variables from administrative data: An intersectional approach to the study of place and child development. *Health and Place*, *16*(3), 500–511. https://doi.org/10.1016/j.healthplace.2009.12.008
311. Khambhaita, P., & Bhopal, K. (2015). Home or away? The significance of ethnicity, class and attainment in the housing choices of female university students. *Race Ethnicity and Education*, *18*(4), 535–566. https://doi.org/10.1080/13613324.2012.759927
312. Khan, M., Ilcisin, M., & Saxton, K. (2017). Multifactorial discrimination as a fundamental cause of mental health inequities. *International Journal for Equity in Health*, *16*(1), 1-12. https://doi.org/10.1186/s12939-017-0532-z
313. Khattab, N., & Hussein, S. (2018). Can religious affiliation explain the disadvantage of Muslim women in the British labour market? *Work, Employment and Society*, *32*(6), 1011–1028. https://doi.org/10.1177/0950017017711099
314. Kim, E. J., Parish, S. L., & Skinner, T. (2019). The impact of gender and disability on the economic well-being of disabled women in the United Kingdom: A longitudinal study between 2009 and 2014. *Social Policy & Administration*, *53*(7), 1064–1080. https://doi.org/10.1111/spol.12486
315. Kim, E. J., Skinner, T., & Parish, S. L. (2019). A study on intersectional discrimination in employment against disabled women in the UK. *Disability & Society, 35*(5), 715-737. https://doi.org/10.1080/09687599.2019.1702506
316. Kim, Y.-M. (2017). Rethinking double jeopardy: Differences in the gender disadvantage between organizational insiders and outsiders in Korea. *Sociological Perspectives*, *60*(6), 1082–1096. https://doi.org/10.1177/0731121417726978
317. Kim, Y., & Calzada, E. J. (2019). Skin color and academic achievement in young, Latino children: Impacts across gender and ethnic group. *Cultural Diversity & Ethnic Minority Psychology*, *25*(2), 220–231. https://doi.org/10.1037/cdp0000230
318. King, M. T., Merrin, G. J., Espelage, D. L., Grant, N. J., & Bub, K. L. (2018). Suicidality and intersectionality among students identifying as nonheterosexual and with a disability. *Exceptional Children*, *84*(2), 141–158. https://doi.org/10.1177/0014402917736261
319. King, T. L., Shields, M., Shakespeare, T., Milner, A., & Kavanagh, A. (2019). An intersectional approach to understandings of mental health inequalities among men with disability. *SSM Population Health*, *9*, 100464. https://doi.org/10.1016/j.ssmph.2019.100464
320. Kivlighan III, D. M., Hooley, I. W., Bruno, M. G., Ethington, L. L., Keeton, P. M., & Schreier, B. A. (2019). Examining therapist effects in relation to clients' race-ethnicity and gender: An intersectionality approach. *Journal of Counseling Psychology*, *66*(1), 122–129. https://doi.org/10.1037/cou0000316

321. Kobayashi, K. M. (2003). Do intersections of diversity matter? An exploration of the relationship between identity markers and health for mid- to later-life Canadians. *Canadian Ethnic Studies/Etudes Ethniques Au Canada*, *35*(3), 85–98.
322. Koc, Y, & Vignoles, V. L. (2016). Global identification predicts gay–male identity integration and well-being among Turkish gay men. *British Journal of Social Psychology*, *55*(4), 643–661. https://doi.org/10.1111/bjso.12160
323. Kohlman, M. H. (2006). Intersection theory: A more elucidating paradigm of quantitative analysis. *Race, Gender & Class*, *13*(3-4), 42–59. https://www.jstor.org/stable/41675172
324. Köllen, T. (2015). The impact of demographic factors on the way lesbian and gay employees manage their sexual orientation at work: An intersectional perspective. *Management Research Review*, *38*(9), 992–1015. https://doi.org/10.1108/MRR-05-2014-0099
325. Koning, S. M., & Ehrenthal, D. B. (2019). Stressor landscapes, birth weight, and prematurity at the intersection of race and income: Elucidating birth contexts through patterned life events. *SSM Population Health*, *8*, 100460. https://doi.org/10.1016/j.ssmph.2019.100460
326. Konkor, I., Lawson, E. S., Antabe, R., McIntosh, M. D., Husbands, W., Wong, J., & Luginaah, I. (2020). An intersectional approach to HIV vulnerabilities and testing among heterosexual African Caribbean and Black men in London, Ontario: Results from the weSpeak Study. *Journal of Racial and Ethnic Health Disparities*, 1-10. https://doi.org/10.1007/s40615-020-00737-3
327. Koons-Witt, B. A., & Schram, P. J. (2006). Does race matter? Examining the relationship between co-offending and victim characteristics for violence incidents involving remale offenders. *Feminist Criminology*, *1*(2), 125–146. http://doi.org/10.1177/1557085105285971
328. Kronberg, A.-K. (2014). Stay or leave? Race, education, and changing returns to the external labor market strategy, 1976-2009. *Work and Occupations*, *41*(3), 305–349. https://doi.org/10.1177/0730888414535218
329. Kule, A., Bumphus, V. W., & Iles, G. (2019). Intersectionality of race, class, and gender in predicting police satisfaction. *Journal of Ethnicity in Criminal Justice*, *17*(4), 321-338. https://doi.org/10.1080/15377938.2019.1658143
330. Kulick, A., Wernick, L. J., Woodford, M. R., & Renn, K. (2016). Heterosexism, depression, and campus engagement among LGBTQ college students: Intersectional differences and opportunities for healing. In *Journal of Homosexuality 64*(8), 1125-1141. https://doi.org/10.1080/00918369.2016.1242333
331. Kuo, Y.-L., Casillas, A., Walton, K. E., Way, J. D., & Moore, J. L. (2020). The intersectionality of race/ethnicity and socioeconomic status on social and emotional skills. *Journal of Research in Personality*, *84*, 103905. https://doi.org/10.1016/j.jrp.2019.103905
332. Lacroix, J., Gagnon, A., & Lortie, V. (2017). At the intersection of gender and national origin: The career trajectories of selected immigrants in Quebec. *Population*, *72*(3), 419–444. https://doi.org/10.3917/popu.1703.0435
333. Lahlah, E., Lens, K. M. E., Bogaerts, S., & van der Knaap, L. M. (2013). When love hurts. Assessing the intersectionality of ethnicity, socio-economic status, parental

connectedness, child abuse, and gender attitudes in juvenile violent delinquency. *Child Abuse and Neglect*, *37*(11), 1034–1049. https://doi.org/10.1016/j.chiabu.2013.07.001
334. Lam, C. B., Stanik, C., & Mchale, S. M. (2017). The development and correlates of gender role attitudes in African American youth. *British Journal of Developmental Psychology 35*(3) 406-419. https://doi.org/10.1111/bjdp.12182
335. Landale, N. S., Oropesa, R. S., & Noah, A. J. (2017). Experiencing discrimination in Los Angeles: Latinos at the intersection of legal status and socioeconomic status. *Social Science. Research*, *67*, 34–48. https://doi.org/10.1016/j.ssresearch.2017.05.003
336. Landstedt, E., & Gådin, K. G. (2012). Seventeen and stressed—Do gender and class matter? *Health Sociology Review,* 21(1), 82-98. https://doi.org/10.5172/hesr.2012.21.1.82
337. Lantz, M. M., & Davis, B. L. (2017). For whom the bills pile: An equity frame for an equity problem. *Training and Education in Professional Psychology,* 11(3), 166–173. http://dx.doi.org/10.1037/tep0000162
338. Lassiter, J. M., & Poteat, T. (2019). Religious coping and depressive symptoms among Black Americans living with HIV: An intersectional approach. *Psychology of Religion and Spirituality*, 12(3), 261-268. https://doi.org/10.1037/rel0000284
339. Lavaysse, L. M., Probst, T. M., & Arena Jr., D. F. (2018). Is more always merrier? Intersectionality as an antecedent of job insecurity. *International Journal of Environment Research and Public Health*, 15(11), 2559. https://doi.org/10.3390/ijerph15112559
340. Lavis, V. (2019). Intersectionality and the prison crisis: What is it as a concept and why does it matter today in understanding current problems in prison? *Prison Service Journal,* 243, 29-35.
341. Le, T. P., Kuo, L., & Yamasaki, V. (2020). Gendered racial microaggressions, feminism, and Asian American women's eating pathology: An intersectional investigation. *Sex Roles*, 83, 127–142. https://doi.org/10.1007/s11199-019-01100-5
342. Lee, H.-W., Robertson, P. J., & Kim, K. (2019). Determinants of job satisfaction among U.S. Federal employees: An investigation of racial and gender differences. *Public Personnel Management*, 49(3), 336-366. https://doi.org/10.1177/0091026019869371
343. Lee, J. (2018). Black LGB identities and perceptions of same-sex marriage. *Journal of Homosexuality*, 65(14), 2005–2027. https://doi.org/10.1080/00918369.2017.1423214
344. Lefevor, G. T., Blaber, I. P., Huffman, C. E., Schow, R. L., Beckstead, A. L., Raynes, M., & Rosik, C. H. (2019). The role of religiousness and beliefs about sexuality in well-being among sexual minority mormons. *Psychology of Religion and Spirituality*, 12(4), 460-470. https://doi.org/10.1037/rel0000261
345. Lefevor, G. T., Janis, R. A., Franklin, A., & Stone, W.-M. (2019). Distress and therapeutic outcomes among transgender and gender nonconforming people of color. *The Counseling Psychologist*, 47(1), 34-58. https://doi.org/10.1177/0011000019827210
346. Lefevor, G. T., Janis, R. A., & Park, S. Y. (2017). Religious and sexual identities: An intersectional, longitudinal examination of change in therapy. *The Counseling Psychologist*, 45(3), 868-899. https://doi.org/10.1177/0011000017702721
347. Lefevor, G. T., Park, S. Y., & Pedersen, T. R. (2018). Psychological distress among sexual and religious minorities: An examination of power and privilege. *Journal of Gay*

& Lesbian Mental Health, 22(2), 90-104. https://doi.org/10.1080/19359705.2017.1418696
348. Lefevor, G. T., Smack, A. C. P., & Giwa, S. (2020). Religiousness, support, distal stressors, and psychological distress among black sexual minority college students. *Journal of GLBT Family Studies*, 16(2), 148-162. https://doi.org/10.1080/1550428X.2020.1723369
349. Lefevor, G. T., Sorrell, S. A., Kappers, G., Plunk, A., Schow, R. L., Rosik, C. H., & Beckstead, A. L. (2020). Same-sex attracted, not LGBQ: The associations of sexual identity labeling on religiousness, sexuality, and health among mormons. *Journal of Homosexuality*, 67(7), 940-964. https://doi.org/10.1080/00918369.2018.1564006
350. Lehavot, K., Beckman, K. L., Chen, J. A., Simpson, T. L., & Williams, E. C. (2019). Race/ethnicity and sexual orientation disparities in mental health, sexism, and social support among women veterans. *Psychology of Sexual Orientation and Gender Diversity,* 6(3), 347–358. https://doi.org/10.1037/sgd0000333
351. Leiber, M. J., & Beaudry-Cyr, M. (2017). The intersection of race/ethnicity, gender and the treatment of probation violators in juvenile justice proceedings. *In Sociology of Crime, Law, and Deviance* (Vol. 22) (pp. 269–290). Emerald Group Publishing Limitd. https://doi.org/10.1108/S1521-613620170000022020
352. Leiber, M. J., Brubaker, S. J., & Fox, K. C. (2009). A closer look at the individual and joint effects of gender and race on juvenile justice decision making. *Feminist Criminology,* 4(4), 333-358. https://doi.org/10.1177/1557085109338564
353. Leinonen, M., Nikkanen, R., & Otonkorpi-Lehtoranta, K. (2018). Organizational change and employee concerns in the Finnish defence forces. *Nordic Journal of Working Life Studies*, 8(3), 135-153.
354. Levant, R. F., & Wong, Y. J. (2013). Race and gender as moderators of the relationship between the endorsement of traditional masculinity ideology and alexithymia: An intersectional perspective. *Psychology of Men & Masculinity,*14(3), 329-333. http://dx.doi.org/10.1037/a0029551
355. LeVasseur, M. T., Kelvin, E. A., & Grosskopf, N. A. (2013). Intersecting identities and the association between bullying and suicide attempt among New York city youths: Results from the 2009 New York city youth risk behavior survey. *American Journal of Public Health,* 103(6), 1082-1089. https://doi.org/10.2105/AJPH.2012.300994
356. Lewis, B. J., Hesse, C. L., Cook, B. C., & Pedersen, C. L. (2020). Sexistential crisis: An intersectional analysis of gender expression and sexual orientation in masculine overcompensation. *Journal of Homosexuality,* 67(1), 58-78. https://doi.org/10.1080/00918369.2018.1525943
357. Lewis, J. A., Williams, M. G., Peppers, E. J., & Gadson, C. A. (2017). Applying intersectionality to explore the relations between gendered racism and health among Black women. *Journal of Counseling Psychologist,* 64(5), 475-486. https://doi.org/10.1037/cou0000231
358. Lien, P. (2015). Reassessing descriptive representation by women and men of color: New evidence at the subnational level. *Urban Affairs Review*, 51(2), 239-262. https://doi.org/10.1177/1078087414529124
359. Liévanos, R. S. (2015). Race, deprivation, and immigrant isolation: The spatial demography of air-toxic clusters in the continental United States. *Social Science Research*, 54, 50–67. https://doi.org/10.1016/j.ssresearch.2015.06.014

360. Liévanos, R. S. (2017). Sociospatial dimensions of water injustice: The distribution of surface water toxic releases in California's Bay-Delta. *Sociological Perspectives*, 60(3), 575–599. https://doi.org/10.1177/0731121416648935
361. Liévanos, R. S. (2018). Impaired water hazard zones: Mapping intersecting environmental health vulnerabilities and polluter disproportionality. *ISPRS International Journal of Geo-Information,* 7(11), 433. https://doi.org/10.3390/ijgi7110433
362. Liévanos, R. S. (2019). Air-toxic clusters revisited: Intersectional environmental inequalities and Indigenous deprivation in the U.S. Environmental Protection Agency Regions. *Race and Social Problems,* 11(2), 161–184. https://doi.org/10.1007/s12552-019-09260-5
363. Liévanos, R. S., & Horne, C. (2017). Unequal resilience: The duration of electricity outages. Energy Policy, 108, 201–211. https://doi.org/10.1016/j.enpol.2017.05.058
364. Liew, H. (2019). Explaining disparities in transitions among visual-functioning states. *Longitudinal and Life Course Studies,* 10(3), 327-345. https://doi.org/10.1332/175795919X15628474680709
365. Lipperman-Kreda, S., Antin, T. M. J., & Hunt, G. P. (2019). The role of multiple social identities in discrimination and perceived smoking-related stigma among sexual and gender minority current or former smokers. *Drugs: Education, Prevention and Policy,* 26(6), 475–483. https://doi.org/10.1080/09687637.2018.1490391
366. Litzler, E., Samuelson, C. C., & Lorah, J. A. (2014). Breaking it down: Engineering student STEM confidence at the intersection of race/ethnicity and gender. *Research in Higher Education,* 55(8), 810–832. https://doi.org/10.1007/s11162-014-9333-z
367. Liu, B. C.-P. (2017). Intersectional impact of multiple identities on social work education in the UK. *Journal of Social Work,* 17(2), 226-242. https://doi.org/10.1177/1468017316637220
368. Liu, H., Reczek, C., Mindes, S. C. H., & Shen, S. (2017). The health disparities of same-sex cohabitors at the intersection of race-ethnicity and gender. *Sociological Perspectives,* 60(3), 620–639. https://doi.org/10.1177/0731121416663685
369. Livingston, J., Patel, N., Bryson, S., Hoong, P., Lal, R., Morrow, M., & Guruge, S. (2018). Stigma associated with mental illness among Asian men in Vancouver, Canada. International Journal of Social Psychiatry, 64(7), 679-689. https://doi.org/10.1177/0020764018805125
370. Lloyd, M. H. (2018). Poverty and family reunification for mothers with substance use disorders in child welfare. Child Abuse Review, 27(4), 301-316. https://doi.org/10.1002/car.2519
371. Loeb, M., Madans, J., Weeks, J., Miller, K., Dahlhamer, J. & Golden, C. (2013), "Disability and participation: assessing employment and education outcomes in the National Health Interview Survey (2010). *In Disability and Intersecting Statuses (Research in Social Science and Disability*) (pp. 171-188). (Vol. 7). Emerald Group Publishing Limited. https://doi.org/10.1108/S1479-3547(2013)0000007008
372. Logan, T D. (2010). Personal characteristics, sexual behaviors, and male sex work: A quantitative approach. American Sociological Review, 75(5), 679-704. https://doi.org/10.1177/0003122410379581
373. Logie, C. H., Wang, Y., Lacombe-Duncan, A., Wagner, A. C., Kaida, A., Conway, T., Webster, K., de Pokomandy, A., & Loutfy, M. R. (2018). HIV-related stigma, racial

discrimination, and gender discrimination: Pathways to physical and mental health-related quality of life among a national cohort of women living with HIV. *Preventive Medicine*, 107, 36–44. https://doi.org/10.1016/j.ypmed.2017.12.018

374. Logie, C. H., Williams, C. C., Wang, Y., Marcus, N., Kazemi, M., Cioppa, L., Kaida, A., Webster, K., Beaver, K., de Pokomandy, A., & Loutfy, M. (2019). Adapting stigma mechanism frameworks to explore complex pathways between intersectional stigma and HIV-related health outcomes among women living with HIV in Canada. *Social Science & Medicine*, 232, 129–138. https://doi.org/10.1016/j.socscimed.2019.04.044
375. Longman Marcellin, R., Bauer, G. R., & Scheim, A. I. (2013). Intersecting impacts of transphobia and racism on HIV risk among trans persons of colour in Ontario, Canada. Ethnicity and Inequalities in Health and Social Care, 6(4), 97-107. https://doi.org/10.1108/EIHSC-09-2013-0017
376. López, N., Erwin, C., Binder, M., & Chavez, M. J. (2018). Making the invisible visible: advancing quantitative methods in higher education using critical race theory and intersectionality. *Race Ethnicity and Education*, 21(2), 180-207. https://doi.org/10.1080/13613324.2017.1375185
377. Lord, S. M., Camacho, M. M., Layton, R. A., Long, R. A., Ohland, M. W., & Wasburn, M. H. (2009). Who's persisting in engineering? A comparative analysis of female and male Asian, Black, Hispanic, Native American, and white students. Journal of Women and Minorities in Science and Engineering, 15(2), 167–190. https://doi.org/10.1615/JWomenMinorScienEng.v15.i2.40
378. Lord, S. M., Ohland, M. W., Layton, R. A., & Camacho, M. M. (2019). Beyond pipeline and pathways: Ecosystem metrics. *Journal of Engineering Education*, 108(1), 32–56. https://doi.org/10.1002/jee.20250
379. Love, C. D., Booysen, L. A. E., & Essed, P. (2018). An exploration of the intersection of race, gender and generation in African American women doing social justice work. *Gender, Work and Organization*, *25*(5), 475–494. https://doi.org/10.1111/gwao.12095
380. Lowery, P. G. (2019). The independent and joint effects of race, crime, and social location on the dispositional decisions of juvenile girls. *Journal of Ethnicity in Criminal Justice*, 17(2), 81–109. https://doi.org/10.1080/15377938.2019.1575780
381. Lytle, M. C., De Luca, S. M., Blosnich, J. R., & Brownson, C. (2015). Associations of racial/ethnic identities and religious affiliation with suicidal ideation among lesbian, gay, bisexual, and questioning individuals. *Journal of Affective Disorders*, 178, 39–45. https://doi.org/10.1016/j.jad.2014.07.039
382. Ma, Y., & Liu, Y. (2017). Entry and degree attainment in STEM: The intersection of gender and race/ethnicity. *Social Sciences*, 6(3), 89. https://doi.org/10.3390/socsci6030089
383. Macdonald, S. J., & Deacon, L. (2019). Twice upon a time: Examining the effect socio-economic status has on the experience of dyslexia in the United Kingdom. *Dyslexia*, 25(1), 3–19. https://doi.org/10.1002/dys.1606
384. Maggard, S. R., Higgins, J. L., & Chappell, A. T. (2013). Pre-dispositional juvenile detention: An analysis of race, gender and intersectionality. *Journal of Crime and Justice*, 36(1), 67–86. https://doi.org/10.1080/0735648X.2011.651793
385. Mahalingam, R., Balan, S., & Haritatos, J. (2008). Engendering immigrant psychology: An intersectionality perspective. *Sex Roles*, 59, 326–336. https://doi.org/10.1007/s11199-008-9495-2

386. Mair, C. A. (2010). Social ties and depression: An intersectional examination of Black and White community-dwelling older adults. *Journal of Applied Gerontology*, 29(6), 667–696. https://doi.org/10.1177/0733464809350167
387. Malmusi, D., Vives, A., Benach, J., & Borrell, C. (2014). Gender inequalities in health: Exploring the contribution of living conditions in the intersection of social class. *Global Health Action*, 7(1), 23189. https://doi.org/10.3402/gha.v7.23189
388. Mamani Ortiz, Y., Gustafsson, P. E., San Sebastián Chasco, M., Armaza Céspedes, A. X., Luizaga López, J. M., Illanes Velarde, D. E., & Mosquera Méndez, P. A. (2019). Underpinnings of entangled ethnical and gender inequalities in obesity in Cochabamba-Bolivia: An intersectional approach. *International Journal for Equity in Health*, 18(1), 1-13. https://doi.org/10.1186/s12939-019-1062-7
389. Mancenido, A., Williams, E. C., & Hajat, A. (2020). Examining psychological distress across intersections of immigrant generational status, race, poverty, and gender. *Community Mental Health Journal*, 56, 1269–1274. https://doi.org/10.1007/s10597-020-00584-w
390. Mandel, H., & Semyonov, M. (2016). Going back in time? Gender differences in trends and sources of the racial pay gap, 1970 to 2010. *American Sociological Review,* 81(5), 1039–1068. https://doi.org/10.1177/0003122416662958
391. Mansouri, F., & Johns, A. (2017). Social networks and perceptions of intergenerational difference among migrant youth in Australia. *Journal of Sociology*, 53(1), 127–144. https://doi.org/10.1177/1440783316636244
392. Manzi, C., Paderi, F., Benet-Martínez, V., & Coen, S. (2019). Age-based stereotype threat and negative outcomes in the workplace: Exploring the role of identity integration. *European Journal of Social Psychology*, 49(4), 705–716. https://doi.org/10.1002/ejsp.2533
393. Marlow, S., Greene, F. J., & Coad, A. (2017). Advancing gendered analyses of entrepreneurship: A critical exploration of entrepreneurial activity among gay men and lesbian women. *British Journal of Management*, 21(1), 118-135. https://doi.org/10.1111/1467-8551.12221
394. Maroto, M., Pettinicchio, D., & Patterson, A. C. (2019). Hierarchies of categorical disadvantage: economic insecurity at the intersection of disability, gender, and race. *Gender and Society*, 33(1), 64–93. https://doi.org/10.1177/0891243218794648
395. Marshal, M. P., Dermody, S. S., Shultz, M. L., Sucato, G. S., Stepp, S. D., Chung, T., Burton, C. M., Markovic, N., & Hipwell, A. E. (2013). Mental health and substance use disparities among urban adolescent lesbian and bisexual girls. *Journal of the American Psychiatric Nurses Association*, 19(5), 271–279. https://doi.org/10.1177/1078390313503552
396. Maxwell, M, Brevard, J., Abrams, J., & Belgrave, F. (2015). What’s color got to do with it? Skin color, skin color satisfaction, racial identity, and internalized racism among African American college students. *Journal of Black Psychology,* 41(5), 438-461. https://doi.org/10.1177/0095798414542299
397. Mazziotta, A., Zerr, M., & Rohmann, A. (2015). The effects of multiple stigmas on discrimination in the German housing market. *Social Psychology*, 46(6), 325–334. https://doi.org/10.1027/1864-9335/a000249
398. McClendon, J., Jackson, J. J., Bogdan, R., & Oltmanns, T. F. (2019). Trajectories of racial and gender health disparities during later midlife: Connections to personality.

Cultural Diversity and Ethnic Minority Psychology, 25(3), 359-370. https://doi.org/10.1037/cdp0000238
399. McCloud, R. F., Jung, M., Gray, S. W., & Viswanath, K. (2013). Class, race and ethnicity and information avoidance among cancer survivors. *British Journal of Cancer*, 108(10), 1949–1956. https://doi.org/10.1038/bjc.2013.182
400. McConnell, E. A., Janulis, P., Phillips, G., Truong, R., & Birkett, M. (2018). Multiple minority stress and LGBT community resilience among sexual minority men. *Psychology of Sexual Orientation and Gender Diversity*, 5(1), 1-12. https://doi.org/10.1037/sgd0000265
401. McGee, M. G. (2014). Lost in the margins? Intersections between disability and other nondominant statuses with regard to peer victimization. *Journal of School Violence,* 13(4), 396–421. https://doi.org/10.1080/15388220.2014.894914
402. McGovern, J. (2017). The boundaries of Latino sport leadership: How skin tone, ethnicity, and nationality construct baseball's color line. *Sociological Inquiry*, 87(1), 49–74. https://doi.org/10.1111/soin.12152
403. McGregor, A. J., Bogart, L. M., Higgins-Biddle, M., Strolovitch, D. Z., & Ojikutu, B. (2019). Marginalized yet mobilized: Race, sexuality, and the role of political hypervigilance in African American political participation in 2016. *Du Bois Review*, 16(1), 131–156. https://doi.org/10.1017/S1742058X19000031
404. McKane, R. G., Satcher, L. A., Houston, S. L., & Hess, D. J. (2018). Race, class, and space: an intersectional approach to environmental justice in New York City. *Environmental Sociology*, 4(1), 79–92. https://doi.org/10.1080/23251042.2018.1429177
405. McLaughlin, J. S. (2020). Falling between the cracks: Discrimination laws and older women. *labour*, 34(2), 215–238. https://doi.org/10.1111/labr.12175
406. Melzer, S. M., Tomaskovic-Devey, D., Schunck, R., & Jacobebbinghaus, P. (2018). A relational inequality approach to first- and second-generation immigrant earnings in German workplaces. *Social Forces*, 97(1), 91–128. https://doi.org/10.1093/SF/SOY021
407. Mereish, E. H. (2012). The intersectional invisibility of race and disability status: An exploratory study of health and discrimination facing Asian Americans with disabilities. *Ethnicity and Inequalities in Health and Social Care*, 5(2), 52-60. https://doi.org/10.1108/17570981211286796
408. Mereish, E. H., & Bradford, J. B. (2014). Intersecting identities and substance use problems: Sexual orientation, gender, race, and lifetime substance use problems. *Journal of Studies on Alcohol and Drugs*, 75(1), 179-188. https://doi.org/10.15288/jsad.2014.75.179
409. Meyer, D., & Grollman, E. A. (2014). sexual orientation and fear at night: Gender differences among sexual minorities and heterosexuals. *Journal of Homosexuality,* 61(4), 453–470. https://doi.org/10.1080/00918369.2013.834212
410. Meyer, S. J. (2019). The use of social services by older males. *Journal of Social Work,* 19(4), 450–467. https://doi.org/10.1177/1468017318757358
411. Michael, K. Y., & Alsup, P. R. (2016). Differences between the sexes among protestant christian middle school students and their attitudes toward Science, Technology, Engineering and Math (STEM). *Journal of Research on Christian Education*, 25(2), 147–168. https://doi.org/10.1080/10656219.2016.1191396

412. Love, C. D., Booysen, L. A. E., & Essed, P. (2018). An exploration of the intersection of race, gender and generation in African American women doing social justice work. Gender, Work and Organization, 25(5), 475–494. https://doi.org/10.1111/gwao.12095
413. Millard, A. D., Raab, G., Lewsey, J., Eaglesham, P., Craig, P., Ralston, K., & McCartney, G. (2015). Mortality differences and inequalities within and between "protected characteristics" groups, in a Scottish Cohort 1991-2009. *International Journal for Equity in Health,* 14(1), 1-14. https://doi.org/10.1186/s12939-015-0274-8
414. Minkler, M., & Fuller-Thomson, E. (2005). African American grandparents raising grandchildren: a national study using the Census 2000 American Community Survey. *The Journals of Gerontology Series B: Psychological Sciences and Social Sciences,* 60(2), S82-92. https://doi.org/10.1093/geronb/60.2.s82
415. Minta, M. D., & Brown, N. E. (2013). Intersecting interests : Gender, race, and congressional attention to women's issues. *Du Bois Review*, 11(2), 253-272. https://doi.org/10.1017/S1742058X14000186
416. Molina, K. M., Alegría, M., & Mahalingam, R. (2013). A multiple-group path analysis of the role of everyday discrimination on self-rated physical health among Latina/os in the USA. *Annals of Behavioral Medicine*, 45(1), 33-43. https://doi.org/10.1007/s12160-012-9421-2
417. Molina, K. M., Jackson, B., & Rivera-Olmedo, N. (2016). Discrimination, racial/ethnic identity, and substance use among latina/os: are they gendered? *Annals of Behavioral Medicine*, 50(1), 119–129. https://doi.org/10.1007/s12160-015-9738-8
418. Monnat, S. M., & Bunyan, L. A. (2008). Capitalism and welfare reform: who really benefits from welfare-to-work policies*? Race, Gender & Class*, 15(1-2), 115–133. https://www.jstor.org/stable/41675361
419. Moodley, J., & Graham, L. (2015). The importance of intersectionality in disability and gender studies. *Agend*a, 29(2), 24–33. https://doi.org/10.1080/10130950.2015.1041802
420. Moody, A. T., & Lewis, J. A. (2019). Gendered racial microaggressions and traumatic stress symptoms among black women. *Psychology of Women Quarterly*, 43(2), 201-214. https://doi.org/10.1177/0361684319828288
421. Moore, K. K., & Ghilarducci, T. (2018). Intersectionality and stratification in the labor market. *Generations*, 42(2), 34-40. https://www.jstor.org/stable/26556358
422. Morales, D. X., Grineski, S. E., & Collins, T. W. (2019). School bullying, body size, and gender: an intersectionality approach to understanding US children's bullying victimization*. British Journal of Sociology of Education*, 40(8), 1121-1137. https://doi.org/10.1080/01425692.2019.1646115
423. Morales, M C. (2009). Ethnic-controlled economy or segregation? Exploring inequality in Latina/o co-ethnic jobsites. *Sociological Forum*, 24(3), 589-610. https://doi.org/10.1111/j.1573-7861.2009.01121.x
424. Moreau, J., Nuño-Pérez, S., & Sanchez, L. M. (2019). Intersectionality, linked fate, and LGBTQ Latinx political participation. *Political Research Quarterly*, 72(4), 976-990. https://doi.org/10.1177/1065912919847293
425. Morris, E. W., & Perry, B. L. (2017). Girls behaving badly? race, gender, and subjective evaluation in the discipline of African American girls. *Sociology of Education*, 90(2), 127–148. https://doi.org/10.1177/0038040717694876

426. Morris, T., Gomez, A., Naiman-Sessions, M., & Morton, C. H. (2018). Paradox lost on the U.S.-Mexico border: U.S. Latinas and cesarean rates. *BMC Pregnancy Childbirth*, 18, 82. https://doi.org/10.1186/s12884-018-1701-9
427. Mosley, E. A., Anderson, B. A., Harris, L. H., Fleming, P. J., & Schulz, A. J. (2019). Attitudes toward abortion, social welfare programs, and gender roles in the U.S. and South Africa. *Critical Public Healt*h, 30(4), 441-456. https://doi.org/10.1080/09581596.2019.1601683
428. Mousaid, S., De Moortel, D., Malmusi, D., & Vanroelen, C. (2016). New perspectives on occupational health and safety in immigrant populations: studying the intersection between immigrant background and gender. *Ethnicity and Health*, 21(3), 251-267. https://doi.org/10.1080/13557858.2015.1061103
429. Mügge, L. M., van der Pas, D. J., & van de Wardt, M. (2019). Representing their own? Ethnic minority women in the Dutch Parliament. *West European Politics*, 42(4), 705-727. https://doi.org/10.1080/01402382.2019.1573036
430. Mukhopadhyay, S. (2015). The intersection of gender, caste and class inequalities in child nutrition in rural India. *Asian Population Studies*, 11(1), 17-31. https://doi.org/10.1080/17441730.2015.995150
431. Mulinari, S., Wemrell, M., Rönnerstrand, B., Subramanian, S. V, & Merlo, J. (2018). Categorical and anti-categorical approaches to US racial/ethnic groupings: Revisiting the National 2009 H1N1 Flu Survey (NHFS*). Critical Public Health*, 28(2), 177-189. https://doi.org/10.1080/09581596.2017.1316831
432. Muñoz-Comet, J., & Steinmetz, S. (2020). Trapped in precariousness? Risks and opportunities of female immigrants and natives transitioning from part-time jobs in Spain*. Work, Employment and Society*, 3 4(5), 749-768. https://doi.org/10.1177/0950017020902974
433. Murzacheva, E., Sahasranamam, S., & Levie, J. (2019). Doubly disadvantaged: Gender, spatially concentrated deprivation and nascent entrepreneurial activity. *European Management Review*, 17(3), 669-685. https://doi.org/10.1111/emre.12370
434. Naqvi, J. B., Helgeson, V. S., Gary-Webb, T. L., Korytkowski, M. T., & Seltman, H. J. (2020). Sex, race, and the role of relationships in diabetes health: Intersectionality matters. *Journal of Behavioral Medicine*, 43(1), 69–79. https://doi.org/10.1007/s10865-019-00057-w
435. Nath, L, Holder-Webb, L., & Cohen, J. (2013). Will women lead the way? Differences in demand for corporate social responsibility information for investment decisions. *Journal of Business Ethics*, 118(1), 85–102.https://doi.org/10.1007/s10551-012-1573-2
436. Navarro, R. L., Flores, L. Y., Legerski, J.-P., Brionez, J., May, S. F., Suh, H. N., Slivensky, D. R., Tapio, F., Lee, H.-S., Garriott, P. O., Hunt, H. K., Desjarlais, C. D., Lee, B.-H., Diaz, D., Zhu, J., & Jung, A.-K. (2019). Social cognitive predictors of engineering students' academic persistence intentions, satisfaction, and engagement. *Journal of Counseling Psychology,* 66(2), 170-183. https://doi.org/10.1037/cou0000319
437. Nawyn, S J, & Park, J. (2019). Gendered segmented assimilation: Earnings trajectories of African immigrant women and men. *Ethnic and Racial Studies*, 42(2), 216-234. https://doi.org/10.1080/01419870.2017.1400085
438. Nawyn, Stephanie J, & Gjokaj, L. (2014). The magnifying effect of privilege: Earnings inequalities at the intersection of gender, race, and nativity. *Feminist Formations*, 26(2), 85–106. https://doi.org/10.1353/ff.2014.0015

439. Neergheen, V. L., Topel, M., Van Dyke, M. E., Sullivan, S., Pemu, P. E., Gibbons, G. H., Vaccarino, V., Quyyumi, A. A., & Lewis, T. T. (2019). Neighborhood social cohesion is associated with lower levels of interleukin-6 in African American women. *Brain Behavior and Immunity*, 76, 28-36. https://doi.org/10.1016/j.bbi.2018.10.008
440. Nejad, M. G., & O'Connor, G. (2016). An intersectional approach to evaluating consumer financial literacy. *Journal of Financial Services Marketing,* 21(4), 308-324. https://doi.org/10.1057/s41264-016-0014-1
441. Nelson, A., & Piatak, J. (2019). Intersectionality, leadership, and inclusion: How do racially underrepresented women fare in the federal government? *Review of Public Personnel Administration*. https://doi.org/10.1177/0734371X19881681
442. Ngoubene-Atioky, A J, Lu, C., Muse, C., & Tokplo, L. (2020). The influence of intersectional identities on the employment integration of Sub-Saharan African women immigrants in the U.S. *Journal of Immigrant & Refugee Studies*, 18(1), 75-94. https://doi.org/10.1080/15562948.2019.1570417
443. Niven, J., Faggian, A., & Ruwanpura, K. N. (2013). Exploring "underachievement" among highly educated young British-Bangladeshi Women. *Feminist Economics*, 19(1), 111–136. https://doi.org/10.1080/13545701.2012.748985
444. Nix, S., & Perez-Felkner, L. (2019). Difficulty orientations, gender, and race/ethnicity: An intersectional analysis of pathways to STEM degrees. *Social Sciences*, 8(2), 43.
445. Nobis, T., & El-Kayed, N. (2019). Social inequality and sport in Germany–A multidimensional and intersectional perspective. *European Journal for Sports and Society,* 16(1), 5–26. https://doi.org/10.1080/16138171.2019.1577328
446. Nooruddin, I. (2007). Blind justice: "Seeing" race and gender in cases of violent crime. *Politics and Gender*, 3(3), 321-348. https://doi.org/10.1017/S1743923X07000293
447. Novak Colwell, J. M., Axelrod, M., Salim, S. S., & Velvizhi, S. (2017). A gendered analysis of fisherfolk's livelihood adaptation and coping responses in the face of a seasonal fishing ban in Tamil Nadu & Puducherry, India. *World Development*, 98, 325. https://doi.org/10.1016/j.worlddev.2017.04.033
448. Nowacki, J. S. (2017). An intersectional approach to race/ethnicity, sex, and age disparity in federal sentencing outcomes: An examination of policy across time periods. *Criminology and Criminal Justice*, 17(1), 97-116. https://doi.org/10.1177/1748895816642502
449. Noy, S., & Ray, R. (2012). Graduate students' perceptions of their advisors: Is there systematic disadvantage in mentorship? *Journal of Higher Education*, 83(6), 876-914. https://doi.org/10.1353/jhe.2012.0036
450. Noy, S., & O'brien, T. L. (2018). An intersectional analysis of perspectives on science and religion in the United States. *The Sociological Quarterly*, 59(1), 40-61. https://doi.org/10.1080/00380253.2017.1383141
451. O'Brien, L. T., Blodorn, A., Adams, G., Garcia, D. M., & Hammer, E. (2015). Ethnic variation in gender-STEM stereotypes and STEM participation: An intersectional approach. *Cultural Diversity and Ethnic Minority Psychology*, 21(2), 169-180. https://doi.org/10.1037/a0037944
452. O'Brien, L. T., Garcia, D. M., Adams, G., Villalobos, J. G., Hammer, E., & Gilbert, P. (2015). The threat of sexism in a STEM educational setting: The moderating impacts of ethnicity and legitimacy beliefs on test performance. *Social Psychology of Education,* 18(4), 667–684. https://doi.org/10.1007/s11218-015-9310-1

453. Oikelome, F., & Healy, G. (2013). Gender, migration and place of qualification of doctors in the uk: perceptions of inequality, morale and career aspiration. *Journal of Ethnic and Migration Studies,* 39(4), 557-577. https://doi.org/10.1080/1369183X.2013.745233
454. Ojeda, C., & Slaughter, C. M. (2019). Intersectionality, depression, and voter turnout. *Journal of Health Politics, Policy and Law,* 44(3), 480-504. https://doi.org/10.1215/03616878-7367036
455. Ojeda, L., Piña-Watson, B., & Gonzalez, G. (2016). The role of social class, ethnocultural adaptation, and masculinity ideology on Mexican American college men's well-being. *Psychology of Men and Masculinity,* 17(4), 373-379. https://doi.org/10.1037/men0000023
456. Ondercin, H. (2013). What Scarlett O'Hara thinks: political attitudes of Southern women. *Political Science Quarterly*, 128(2), 233-359. https://www.jstor.org/stable/23563305?seq=1
457. Oney, C. N., Cole, E. R., & Sellers, R. M. (2011). Racial identity and gender as moderators of the relationship between body image and self-esteem for African Americans. *Sex Roles*, 65(7), 619–631. https://doi.org/10.1007/s11199-011-9962-z
458. Orey, B. D., Smooth, W., Adams, K. S., & Harris-Clark, K. (2007). Race and gender matter: Refining models of legislative policy making in state legislatures. *Journal of Women, Politics and Policy,* 28(3-4), 97–119. https://doi.org/10.1300/J501v28n03_05
459. Orr, L., Shebl, F. M., Heimer, R., Khoshnood, K., Barbour, R., Khouri, D., Aaraj, E., Mokhbat, J. E., & Crawford, F. W. (2019). Violence and discrimination against men who have sex with men in Lebanon: The role of international displacement and migration. *Journal of Interpersonal Violence.* https://doi.org/10.1177/0886260519884684
460. Orr, M. K., Ramirez, N. M., Lord, S. M., Layton, R. A., & Ohland, M. W. (2015). Student choice and persistence in aerospace engineering. *Journal of Aerospace Information Systems*, 12(4), 365–373. https://doi.org/10.2514/1.I010343
461. Ortiz, K., Cuevas, A. G., Salloum, R., Lopez, N., & LaVeist-Ramos, T. (2019). Intra-ethnic racial differences in waterpipe tobacco smoking among Latinos? *Substance Use & Misuse*, 54(1), 1–10. https://doi.org/10.1080/10826084.2018.1480040
462. Otonkorpi-Lehtoranta, K., Leinonen, M., Nikkanen, R., & Heiskanen, T. (2015). Intersections of gender, age and occupational group in the Finnish Defence Forces. *Equality, Diversity and Inclusion,* 34(7), 593–607. https://doi.org/10.1108/EDI-03-2015-0016
463. Owusu, M., Nursey-Bray, M., & Rudd, D. (2019). Gendered perception and vulnerability to climate change in urban slum communities in Accra, Ghana. *Regional Environmental Change,* 19(1), 13–25. https://doi.org/10.1007/s10113-018-1357-z
464. Paat, Y.-F. (2015). Parental depressive tendencies, gender, and immigrant status in fragile families. *International Journal of Culture and Mental Health*, 8(4), 396-413. https://doi.org/10.1080/17542863.2015.1034739
465. Pachankis, J. E., Hatzenbuehler, M. L., Berg, R. C., Fernández-Dávila, P., Mirandola, M., Marcus, U., Weatherburn, P., & Schmidt, A. J. (2017). Anti-LGBT and anti-immigrant structural stigma: An intersectional analysis of sexual minority men's HIV risk when migrating to or within Europe. *Journal of Acquired Immune Deficiency Syndromes*, 76(4), 356–366. https://doi.org/10.1097/QAI.0000000000001519

466. Pachankis, J. E., Hatzenbuehler, M. L., Wang, K., Burton, C. L., Crawford, F. W., Phelan, J. C., & Link, B. G. (2018). The burden of stigma on health and well-being: A taxonomy of concealment, course, disruptiveness, aesthetics, origin, and peril across 93 stigmas. *Personality and Social Psychology Bulletin*, 44(4), 451-474. https://doi.org/10.1177/0146167217741313
467. Pal, G. C. (2011). Disability, intersectionality and deprivation: An excluded agenda. *Psychology and Developing Societies*, 23(2), 159-176. https://doi.org/10.1177/097133361102300202
468. Panchap, L., Safavynia, S. A., Tangel, V., & White, R. S. (2020). Socioeconomic disparities in carotid revascularization procedures. *Journal of Cardiothoracic and Vascular Anesthesia,* 34(7), 1836-1845. https://doi.org/10.1053/j.jvca.2019.11.038
469. Parashar, S. (2014). Marginalized by race and place: A multilevel analysis of occupational sex segregation in post-apartheid South Africa*.* *International Journal of Sociology and Social Policy,* 34(11-12), 747–770. https://doi.org/10.1108/IJSSP-01-2014-0003
470. Parent, M. C., Hammer, J. H., Bradstreet, T. C., Schwartz, E. N., & Jobe, T. (2018). Men's mental health help-seeking behaviors: An intersectional analysis. *American Journal of Men's Health*, 12(1), 64–73. https://doi.org/10.1177/1557988315625776
471. Parker, K. F., & Hefner, M. K. (2015). Intersections of race, gender, disadvantage, and violence: Applying intersectionality to the macro-level study of female homicide. *Justice Quarterly*, 32(2), 223–254. https://doi.org/10.1080/07418825.2012.761719
472. Parra, L. A., & Hastings, P. D. (2020). Challenges to identity integration indirectly link experiences of heterosexist and racist discrimination to lower waking salivary cortisol in sexually diverse Latinx emerging adults. *Frontiers in Psychology*, 11, 228. https://doi.org/10.3389/fpsyg.2020.00228
473. Patterson, A. C., & Veenstra, G. (2016). Black-White health inequalities in Canada at the intersection of gender and immigration. *Canadian Journal of Public Health,* 107(3), e278–e284. https://doi.org/10.17269/CJPH.107.5336
474. Pearl, R. L., Wadden, T. A., Tronieri, J. S., Chao, A. M., Alamuddin, N., & Berkowitz, R. I. (2018). Everyday discrimination in a racially diverse sample of patients with obesity. *Clinical Obesity,* 8(2), 140–146. https://doi.org/10.1111/cob.12235
475. Peck, J. H., Leiber, M. J., & Brubaker, S. J. (2014). Gender, race, and juvenile court outcomes: An examination of status offenders. *Youth Violence and Juvenile Justice,* 12(3), 250–267. https://doi.org/10.1177/1541204013489713
476. Peguero, A. A., & Popp, A. M. (2012). Youth violence at school and the intersection of gender, race, and ethnicity. *Journal of Criminal Justice*, 40(1), 1–9. https://doi.org/10.1016/j.jcrimjus.2011.11.005
477. Pérez, L. M., & Llanos, P. M. (2017). Vulnerable women in a thriving country: An analysis of twenty-first-century domestic workers in Peru and recommendations for future research. *Latin American Research Review*, 52(4), 552–570. https://doi.org/10.25222/larr.67
478. Perry, B L, Harp, K. L. H., & Oser, C. B. (2013). Racial and gender discrimination in the stress process: Implications for African American women's health and well-being. *Sociological Perspectives*, 56(1), 25–48. https://doi.org/10.1525/sop.2012.56.1.25
479. Persmark, A., Wemrell, M., Evans, C. R., Subramanian, S. V, Leckie, G., & Merlo, J. (2019). Intersectional inequalities and the U.S. opioid crisis: Challenging dominant

narratives and revealing heterogeneities. *Critical Public Health*, 30(4), 398-414. https://doi.org/10.1080/09581596.2019.1626002

480. Persmark, A., Wemrell, M., Zettermark, S., Leckie, G., Subramanian, S. V, & Merlo, J. (2019). Precision public health: Mapping socioeconomic disparities in opioid dispensations at Swedish pharmacies by Multilevel Analysis of Individual Heterogeneity and Discriminatory Accuracy (MAIHDA). *PLoS ONE*, 14(8). https://doi.org/10.1371/journal.pone.0220322
481. Petts, A. L., & Petts, R. J. (2019). Gender matters: Racial variation and marital stability among intraracial couples. *Journal of Family Issues*, 40(13), 1808–1831. https://doi.org/10.1177/0192513X19849631
482. Phillips, C. (2018). Wanting, and weighting: White women and descriptive representation in the 2016 presidential election. *Journal of Race, Ethnicity and Politics*, 3(1), 29–51. https://doi.org/10.1017/rep.2017.39
483. Potter, L., Zawadzki, M. J., Eccleston, C. P., Cook, J. E., Snipes, S. A., Sliwinski, M. J., & Smyth, J. M. (2019). The intersections of race, gender, age, and socioeconomic status: Implications for reporting discrimination and attributions to discrimination. *Stigma and Health*, 4(3), 264–281. https://doi.org/10.1037/sah0000099
484. Powers, S. L., Lee, K. J., Pitas, N. A., Graefe, A. R., & Mowen, A. J. (2019). Understanding access and use of municipal parks and recreation through an intersectionality perspective. *Journal of Leisure Research*, 51(4), 377-396. https://doi.org/10.1080/00222216.2019.1701965
485. Preston, V., Kim, A., Hudyma, S., Mandell, N., Luxton, M., & Hemphill, J. (2012). Gender, race, and immigration: Aging and economic security in Canada. *Canadian Review of Social Policy/Revue Canadienne de Politique Sociale*, 68-69, 90.
486. Price, M., Polk, W., Hill, N. E., Liang, B., & Perella, J. (2019). The intersectionality of identity-based victimization in adolescence: A person-centered examination of mental health and academic achievement in a U.S. high school. *Journal of adolescence,* 76, 185-196. https://doi.org/10.1016/j.adolescence.2019.09.002
487. Price-Glynn, K., & Rakovski, C. (2012). Who rides the glass escalator? Gender, race and nationality in the National Nursing Assistant study. *Work, Employment and Society*, 26(5), 699–715. https://doi.org/10.1177/0950017012451634
488. Proctor, S. L., Kyle, J., Fefer, K., & Lau, Q. C. (2018). examining racial microaggressions, race/ethnicity, gender, and bilingual status with school psychology students: The role of intersectionality. *Contemporary School Psychology*, 22(3), 355–368. https://doi.org/10.1007/s40688-017-0156-8
489. Quandt, A. (2019). Variability in perceptions of household livelihood resilience and drought at the intersection of gender and ethnicity. *Climatic Change*, 152, 1-15. https://doi.org/10.1007/s10584-018-2343-7
490. Quiton, R. L., Leibel, D. K., Boyd, E. L., Waldstein, S. R., Evans, M. K., & Zonderman, A. B. (2020). Sociodemographic patterns of pain in an urban community sample: An examination of intersectional effects of sex, race, age, and poverty status. *Pain*, 161(5), 1044–1051. https://doi.org/10.1097/j.pain.0000000000001793
491. Radey, M. (2017). Unmarried mothers' postnatal school enrollment: The role and intersection of demographic and socioeconomic characteristics. *Journal of Social Service Research*, 43(1), 115–128. https://doi.org/10.1080/01488376.2016.1216918

492. Radey, M., & Cheatham, L. P. (2013). Do single mothers take their share?: FAFSA completion among aid-eligible female students. *Journal of Diversity in Higher Education*, 6(4), 261–275. https://doi.org/10.1037/a0035089
493. Rahman, L., Du Mont, J., O'Campo, P., & Einstein, G. (2020). Currently married women's present experiences of male intimate partner physical violence in Bangladesh: An intercategorical intersectional approach. *Global Public Health*, 15(1), 121–136. https://doi.org/10.1080/17441692.2019.1649447
494. Rahman, L., Du Mont, J., O'Campo, P., & Einstein, G. (2020). Intersectional community correlates of married women's experiences of male intimate partner physical violence in Bangladesh: A cross-sectional study. *Journal of Epidemiology and Community Health*, *74*(2), 182–189. https://doi.org/10.1136/jech-2019-212295
495. Rakovski, C. & Price-Glynn, K. (2012). "Intersectional Identities and Worker Experiences in Home Health Care: The National Home Health Aide Survey", Jacobs Kronenfeld, J. (Ed.), *Issues in Health and Health Care Related to Race/Ethnicity, Immigration, SES and Gender (Research in the Sociology of Health Care)* (Vol. 30). (pp. 261-280). Emerald Group Publishing Limited. https://doi.org/10.1108/S0275-4959(2012)0000030014
496. Rakovski, C C, & Price-Glynn, K. (2010). Caring labour, intersectionality and worker satisfaction: An analysis of the National Nursing Assistant Study (NNAS). *Sociology of Health and Illness*, 32(3), 400–414. https://doi.org/10.1111/j.1467-9566.2009.01204.x
497. Ramirez, M., & Kim, J. (2018). Traversing gender, sexual orientation, and race-ethnicity: Sexual victimization in a population-based sample of older adults. *Journal of Gay & Lesbian Social Services*, 30(2), 192–208. https://doi.org/10.1080/10538720.2018.1445054
498. Ravera, F., Martín-López, B., Pascual, U., & Drucker, A. (2016). The diversity of gendered adaptation strategies to climate change of Indian farmers: A feminist intersectional approach. *Ambio*, 45, 335–351. https://doi.org/10.1007/s13280-016-0833-2
499. Ray, R. (2017). Black people don't exercise in my neighborhood: Perceived racial composition and leisure-time physical activity among middle class Blacks and Whites. *Social Science Research*, 66, 42–57. https://doi.org/10.1016/j.ssresearch.2017.03.008
500. Read, J. G., & Eagle, D. E. (2011). Intersecting identities as a source of religious incongruence. *Journal for the Scientific Study of Religion*, 50(1), 116–132. https://doi.org/10.1111/j.1468-5906.2010.01555.x
501. Reese, C. C. (2019). The status of public sector pay equity for women of color in the United States. *Review of Public Personnel Administration*, 39(4), 594–610. https://doi.org/10.1177/0734371X18761123
502. Reingold, B., & Smith, A. R. (2012). Welfare policymaking and intersections of race, ethnicity, and gender in U.S. state legislatures. *American Journal of Political Science*, 56(1), 131–147. https://doi.org/10.1111/j.1540-5907.2011.00569.x
503. Reingold, B., Widner, K., & Harmon, R. (2019). Legislating at the Intersections: Race, gender, and representation. *Political Research Quarterly*, 73(4), 819–833. https://doi.org/10.1177/1065912919858405
504. Reinhardt, G. Y. (2019). The intersectionality of disasters' effects on trust in public officials. *Social Science Quarterly*, 100(7), 2567–2580. https://doi.org/10.1111/ssqu.12727

505. Reisen, C. A., Brooks, K. D., Zea, M. C., Poppen, P. J., & Bianchi, F. T. (2013). Can additive measures add to an intersectional understanding? Experiences of gay and ethnic discrimination among HIV-positive Latino gay men. *Cultural Diversity and Ethnic Minority Psychology*, 19(2), 208–217. https://doi.org/10.1037/a0031906
506. Remedios, J. D., & Snyder, S. H. (2018). Intersectional oppression: Multiple stigmatized identities and perceptions of invisibility, discrimination, and stereotyping. *Journal of Social Issues*, 74(2), 265–281. https://doi.org/10.1111/josi.12268
507. Richardson, L. J., & Brown, T. H. (2016). (En)gendering racial disparities in health trajectories: A life course and intersectional analysis. *SSM – Population Health*, 2, 425–435. https://doi.org/10.1016/j.ssmph.2016.04.011
508. Richman, A. D. (2018). Concurrent social disadvantages and chronic inflammation: the intersection of race and ethnicity, gender, and socioeconomic status. *Journal of Racial and Ethnic Health Disparities,* 5(4), 787–797. https://doi.org/10.1007/s40615-017-0424-3
509. Riegle-Crumb, C., Peng, M., & Russo-Tait, T. (2020). Committed to STEM? Examining factors that predict occupational commitment among Asian and White female students completing stem U.S. postsecondary programs. *Sex Roles,* 82, 102–116. https://doi.org/10.1007/s11199-019-01038-8
510. Ro, H. K., Kim, S., & Bergom, I. (2019). Political engagement among undergraduate women of color. *Journal of Student Affairs Research and Practice*, 56(5), 564–581. https://doi.org/10.1080/19496591.2019.1669453
511. Ro, H. K., & Loya, K. I. (2015). The effect of gender and race intersectionality on student learning outcomes in engineering. *Review of Higher Education,* 38(3), 359–396. https://doi.org/10.1353/rhe.2015.0014
512. Roberts, L., Tamene, M., & Orta, O. R. (2018). The intersectionality of racial and gender discrimination among teens exposed to dating violence. *Ethnicity & Disease,* 28(Suppl 1), 253–260. https://doi.org/10.18865/ed.28.S1.253
513. Robnett, R. D., Nelson, P. A., Zurbriggen, E. L., Crosby, F. J., & Chemers, M. M. (2019). The form and function of STEM research mentoring: A mixed-methods analysis focusing on ethnically diverse undergraduates and their mentors. *Emerging Adulthood*, 7(3), 180–193. https://doi.org/10.1177/2167696818758734
514. Robson, K., Anisef, P., Brown, R. S., & Nagaoka, J. (2019). A comparison of factors determining the transition to postsecondary education in Toronto and Chicago. *Research in Comparative and International Education*, 14(3), 338–356. https://doi.org/10.1177/1745499919865140
515. Robson, K. L., Anisef, P., Brown, R. S., & Parekh, G. (2014). The intersectionality of postsecondary pathways: The case of high school students with special education needs. *Canadian Review of Sociology*, 51(3), 193–215. https://doi.org/10.1111/cars.12044
516. Rodriguez, E. M., Lytle, M. C., & Vaughan, M. D. (2013). Exploring the intersectionality of bisexual, religious/spiritual, and political identities from a feminist perspective. *Journal of Bisexuality*, 13(3), 285–309. https://doi.org/10.1080/15299716.2013.813001
517. Rodriguez-Modroño, P. (2019). Youth unemployment, NEETs and structural inequality in Spain. *International Journal of Manpower*, 40(3), 433–448. https://doi.org/10.1108/IJM-03-2018-0098

518. Rodriguez-Seijas, C., Eaton, N. R., & Pachankis, J. E. (2019). Prevalence of psychiatric disorders at the intersection of race and sexual orientation: Results from the National Epidemiologic Survey of Alcohol and Related Conditions-III. *Journal of Consulting and Clinical Psychology*, 87(4), 321–331. https://doi.org/10.1037/ccp0000377
519. Rogers, L. O., Scott, M. A., & Way, N. (2015). Racial and gender identity among black adolescent males: An intersectionality perspective. *Child Development*, 86(2), 407–424. https://doi.org/10.1111/cdev.12303
520. Rogers, S. A., & Rogers, B. A. (2020). Expanding our view: Demographic, behavioral, and contextual factors in college sexual victimization. *Journal of Interpersonal Violence*. https://doi.org/10.1177/0886260520905076
521. Ropes Berry, K., Kennedy, S. C., Lloyd, M., Veeh, C. A., & Tripodi, S. J. (2020). The intersectional effects of race and gender on time to reincarceration. *Justice Quarterly*, 37(1), 132–160. https://doi.org/10.1080/07418825.2018.1524508
522. Rosenfield, S. (2012). Triple jeopardy? Mental health at the intersection of gender, race, and class. *Social Science and Medicine*, 74(11), 1791–1801. https://doi.org/10.1016/j.socscimed.2011.11.010
523. Rosenthal, L., & Lobel, M. (2020). Gendered racism and the sexual and reproductive health of Black and Latina Women. *Ethnicity and Health*, 25(3), 367–392. https://doi.org/10.1080/13557858.2018.1439896
524. Roxburgh, S. (2014). Race, class, and gender differences in the marriage-health relationship. *Race, Gender & Class*, 21(3-4), 7–31. https://www.jstor.org/stable/43496982
525. Roy, M., Bhatta, T., & Burnette, J. D. (2020). Intersectional effects of gender, education, and geographic region on functional health disparities among older adults in India. *Journal of Aging and Health*, 32(9), 1188-1197. https://doi.org/10.1177/0898264319899246
526. Sachs, D., Sa'ar, A., & Aharoni, S. (2007). "How can I feel for others when I myself am beaten?" The impact of the armed conflict on women in Israel. *Sex Roles*, 57(7-8), 593–606. https://doi.org/10.1007/s11199-007-9222-4
527. Saint Onge, J. M., Cepeda, A., Lee King, P. A., & Valdez, A. (2013). The relationship between trajectories of family/cultural stressors and depression and suicidal ideation among substance using Mexican-American adults. *American Journal of Community Psychology*, 52(3-4), 263–272. https://doi.org/10.1007/s10464-013-9592-4
528. Samuels-Dennis, J. A., Ford-Gilboe, M., Wilk, P., Avison, W. R., & Ray, S. (2010). Cumulative trauma, personal and social resources, and post-traumatic stress symptoms among income-assisted single mothers. *Journal of Family Violence*, 25(6), 603–617. https://doi.org/10.1007/s10896-010-9323-7
529. Sanders, M. R., & Mahalingam, R. (2012). Social dominance orientation and John Henryism at the intersection of race and class. *Political Psychology*, 33(4), 553–573. https://doi.org/10.1111/j.1467-9221.2012.00889.x
530. Sangalang, C. C., & Gee, G. C. (2015). Racial discrimination and depressive symptoms among Cambodian American adolescents: The role of gender. *Journal of Community Psychology*, 43(4), 447–465. https://doi.org/10.1002/jcop.21696
531. Saw, G., Chang, C.-N., & Chan, H.-Y. (2018). Cross-sectional and longitudinal disparities in STEM career aspirations at the intersection of gender, race/ethnicity, and

socioeconomic status. *Educational Researcher*, 47(8), 525–531. https://doi.org/10.3102/0013189X18787818
532. Scheuerman, H. L. (2018). Intersectionality and crime: An exploratory look at how gender and race influence responses to injustice associated with strain. *Deviant Behavior*, 40(11), 1349-1371. https://doi.org/10.1080/01639625.2018.1512218
533. Schnabel, L. (2016). The gender pray gap: Wage labor and the religiosity of high-earning women and men. *Gender and Society*, 30(4), 643–669. https://doi.org/10.1177/0891243216644884
534. Schnabel, L, Hackett, C., & McClendon, D. (2018). Where men appear more religious than women: Turning a gender lens on religion in Israel. *Journal for the Scientific Study of Religion*, 57(1), 80–94. https://doi.org/10.1111/jssr.12498
535. Schug, J., Alt, N. P., Lu, P. S., Gosin, M., & Fay, J. L. (2017). Gendered race in mass media: Invisibility of Asian men and Black Women in popular magazines. *Psychology of Popular Media Culture*, 6(3), 222–236. https://doi.org/10.1037/ppm0000096
536. Scolese, A., Asghar, K., Pla Cordero, R., Roth, D., Gupta, J., & Falb, K. L. (2020). Disability status and violence against women in the home in North Kivu, Democratic Republic of Congo. *Global Public Health*, 1 5(7), 985-998. https://doi.org/10.1080/17441692.2020.1741661
537. Scott, A., & Martin, A. (2014). Perceived barriers to higher education in science, technology, engineering, and mathematics. *Journal of Women and Minorities in Science and Engineering*, 20(3), 235–256. https://doi.org/10.1615/JWomenMinorScienEng.2014006999
538. Scott, J. C., Pinderhughes, E. E., & Johnson, S. K. (2019). How does racial context matter?: Family preparation-for-bias messages and racial coping reported by Black youth. *Child Development*, 91(5), 1471-1490. https://doi.org/10.1111/cdev.13332
539. Seaton, E. K., Caldwell, C. H., Sellers, R. M., & Jackson, J. S. (2010). An intersectional approach for understanding perceived discrimination and psychological well-being among African American and Caribbean black youth. *Developmental Psychology*, 46(5), 1372–1379. https://doi.org/10.1037/a0019869
540. Sen, G, & Iyer, A. (2012). Who gains, who loses and how: Leveraging gender and class intersections to secure health entitlements. *Social Science and Medicine*, 74(11), 1802–1811. https://doi.org/10.1016/j.socscimed.2011.05.035
541. Seng, J. S., Lopez, W. D., Sperlich, M., Hamama, L., & Reed Meldrum, C. D. (2012). Marginalized identities, discrimination burden, and mental health: Empirical exploration of an interpersonal-level approach to modeling intersectionality. *Social Science and Medicine*, 75(12), 2437–2445. https://doi.org/10.1016/j.socscimed.2012.09.023
542. Seo, E., Shen, Y., & Alfaro, E. C. (2019). Adolescents' beliefs about math ability and their relations to STEM career attainment: Joint consideration of race/ethnicity and gender. *Journal of Youth and Adolescence*, 48(2), 306–325. https://doi.org/10.1007/s10964-018-0911-9
543. Settles, I. H. (2006). Use of an intersectional framework to understand Black women's racial and gender identities. *Sex Roles*, 54, 589–601. https://doi.org/10.1007/s11199-006-9029-8
544. Shangani, S., Gamarel, K. E., Ogunbajo, A., Cai, J., & Operario, D. (2020). Intersectional minority stress disparities among sexual minority adults in the USA: The

role of race/ethnicity and socioeconomic status. *Culture, Health & Sexuality*, 22(4), 398–412. https://doi.org/10.1080/13691058.2019.1604994
545. Shaw, L. R., Chan, F., & McMahon, B. T. (2012). Intersectionality and disability harassment: The interactive effects of disability, race, age, and gender. *Rehabilitation Counseling Bulletin*, 55(2), 82–91. https://doi.org/10.1177/0034355211431167
546. Shi, Q., & Watkinson, J. (2019). English language learners and school belonging: Implications for school counselor practice. *Professional School Counseling*, 22(1b). https://doi.org/10.1177/2156759X19834442
547. Shitima, C. M. (2018). Intersectionality and an intra-household analysis of the freedom to make decisions on the use of household products: Evidence from rural Tanzania. *Journal of International Women's Studies,* 19(6), 207–223. https://www.scopus.com/inward/record.uri?eid=2-s2.0-85082360030&partnerID=40&md5=203b982db52be318c923c77b31531829
548. Shramko, M., Toomey, R. B., & Anhalt, K. (2018). Profiles of minority stressors and identity centrality among sexual minority Latinx youth. *American Journal of Orthopsychiatry,* 88(4), 471–482. https://doi.org/10.1037/ort0000298
549. Sicotte, D. (2014). Diversity and intersectionality among environmentally burdened communities in the Philadelphia Metropolitan Area, USA. *Urban Studies*, 51(9), 1850–1870. *https://doi.org/10.1177/0042098013502827*
550. Sieben, A, Renner, L. M., Lust, K., Vang, W., & Nguyen, R. H. N. (2020). Adverse childhood experiences Among Asian/Pacific Islander sexual minority college students. *Journal of Family Violence*, 35(3), 297–303. https://doi.org/10.1007/s10896-019-00095-7
551. Siembida, E. J., Eaton, L. A., Maksut, J. L., Driffin, D. D., & Baldwin, R. (2016). A comparison of HIV-related risk factors between Black transgender women and Black men who have sex with men. *Transgender Health*, 1(1), 172-180. https://doi.org/10.1089/trgh.2016.0003
552. Sikora, J., & Biddle, N. (2015). How gendered is ambition? Educational and occupational plans of Indigenous youth in Australia. *International Journal of Educational Development,* 42(PG-1-13), 1-13. https://doi.org/10.1016/j.ijedudev.2015.02.011
553. Silva, A., & Skulley, C. (2019). Always running: Candidate emergence among women of color over time. *Political Research Quarterly*, 72(2), 342-359. https://doi.org/10.1177/1065912918789289
554. Silva, T., & Evans, C. R. (2020). Sexual identification in the United States at the intersections of gender, race/ethnicity, immigration, and education. *Sex Roles*, 83, 722-738. https://doi.org/10.1007/s11199-020-01145-x
555. Siordia, C. (2015). Prevalence of self-care and ambulatory disability in baby boom and generation-x birth-cohorts by intersectional markers of social stratification. *Race and Social Problems*, 7(4), 257–268. https://doi.org/10.1007/s12552-015-9155-4
556. Siordia, C., & Leyser-Whalen, O. (2017). Average age at last live birth in 'lucky few' and 'late baby boom' birth cohorts in the United States. *Journal of Family Studies*, 23(3), 296–308. https://doi.org/10.1080/13229400.2015.1106338
557. Smith, R. A. (2005). Do the determinants of promotion differ for white men versus women and minorities? an exploration of intersectionalism through sponsored and

contest mobility processes. *American Behavioral Scientist*, 48(9), 1157–1181. https://doi.org/10.1177/0002764205274814
558. Smith, R. A. (2012). Money, benefits, and power: A test of the glass ceiling and glass escalator hypotheses. *Annals of the American Academy of Political and Social Science*, 639(1), 149–172. https://doi.org/10.1177/0002716211422038
559. Smolen, J. R., De Araújo, E. M., De Oliveira, N. F., & De Araújo, T. M. (2018). Intersectionality of race, gender, and common mental disorders in northeastern Brazil. *Ethnicity & Disease*, 28(3), 207–214. https://doi.org/10.18865/ed.28.3.207
560. Sokoloff, N. J., & Pearce, S. C. (2011). Intersections, immigration, and partner violence: A view from a new gateway-Baltimore, Maryland. *Women and Criminal Justice*, 21(3), 250–266. https://doi.org/10.1080/08974454.2011.584468
561. Solazzo, A. L. (2019). Different and not equal: The uneven association of race, poverty, and abortion laws on abortion timing. *Social Problems*, 66(4), 519–547. https://doi.org/10.1093/socpro/spy015
562. Sridharan, S., Pereira, A., Hay, K., Dey, A., Chandurkar, D., Veldhuizen, S., & Nakaima, A. (2018). Heterogeneities in utilization of antenatal care in Uttar Pradesh, India: the need to contextualize interventions to individual contexts.*Global Health Action*, 11(1), 1517929. https://doi.org/10.1080/16549716.2018.1517929
563. Stanton, M. C., Werkmeister Rozas, L., & Asencio, M. (2019). Citizenship status matters: a Social factor influencing outness among a diverse national sample of LGBT individuals. *The British Journal of Social Work*, 49(3), 722-741. https://doi.org/10.1093/bjsw/bcy079
564. Steele, S. M., Collier, M., & Sumerau, J. E. (2018). Lesbian, gay, and bisexual contact with police in Chicago: Disparities across sexuality, race, and socioeconomic status. *Social Currents*, 5(4), 328–349. https://doi.org/10.1177/2329496517748332
565. Steele, S. M., Everett, B. G., & Hughes, T. L. (2020). Influence of perceived femininity, masculinity, race/ethnicity, and socioeconomic status on intimate partner violence among sexual-minority women. *Journal of Interpersonal Violence*, 35(1-2), 453-475. https://doi.org/10.1177/0886260516683176
566. Steffensmeier, D., Painter-Davis, N., & Ulmer, J. (2017). Intersectionality of race, ethnicity, gender, and age on criminal punishment. *Sociological Perspectives*, 60(4), 810–833. https://doi.org/10.1177/0731121416679371
567. Steinmetz, K. F., & Henderson, H. (2015). On the precipice of intersectionality: the influence of race, gender, and offense severity interactions on probation outcomes. *Criminal Justice Review*, 40(3), 361–377. https://doi.org/10.1177/0734016815577194
568. Stevens, N. R., Heath, N. M., Lillis, T. A., McMinn, K., Tirone, V., & Sha'ini, M. (2018). Examining the effectiveness of a coordinated perinatal mental health care model using an intersectional-feminist perspective. *Journal of Behavioral Medicine*, 41(5), 627–640. https://doi.org/10.1007/s10865-018-9973-0
569. Stokes-Brown, A. K., & Dolan, K. (2010). Race, gender, and symbolic representation: African American female candidates as mobilizing agents. *Journal of Elections, Public Opinion and Parties*, 20(4), 473–494. https://doi.org/10.1080/17457289.2010.511806
570. Stoll, L. C., & Block Jr., R. (2015). Intersectionality and cyberbullying: A study of cybervictimization in a Midwestern high school. *Computers in Human Behavior*, 52, 387–397. https://doi.org/10.1016/j.chb.2015.06.010

571. Storer, H. L., Mienko, J. A., Chang, Y.-L., Kang, J. Y., Miyawaki, C., & Schultz, K. (2012). Moving beyond dichotomies: How the intersection of race, class and place impacts high school graduation rates for African American students. *Journal of Sociology and Social Welfare*, 39(1), 17-44. https://www.scopus.com/inward/record.uri?eid=2-s2.0-84863282070&partnerID=40&md5=3611085ba94a319e4bdd2a08982a36c1
572. Stotzer, R. L. (2014). The intersection of suspect and victim race/ethnicity among anti-gay and anti-lesbian bias crimes. *Psychology and Sexuality*, 5(4), 357–373. https://doi.org/10.1080/19419899.2013.843580
573. Stout, C. T., Coulter, K., & Edwards, B. (2017). Blackrepresentation, intersectionality, and politicians' responses to black social movements on twitter. *Mobilization*, 22(4), 493–509.
574. Stout, C. T., Kretschmer, K., & Ruppanner, L. (2017). Gender linked fate, race/ethnicity, and the marriage gap in American politics. *Political Research Quarterly*, 70(3), 509–522. https://doi.org/10.1177/1065912917702499
575. Stout, J. G., & Wright, H. M. (2016). Lesbian, gay, bisexual, transgender, and queer students' sense of belonging in computing: An intersectional approach. *Computing in Science and Engineering*, 18(3), 24–30. https://doi.org/10.1109/MCSE.2016.45
576. Strand, S. (2014). Ethnicity, gender, social class and achievement gaps at age 16: Intersectionality and "getting it" for the white working class. *Research Papers in Education*, 29(2), 131–171. https://doi.org/10.1080/02671522.2013.767370
577. Stypińska, J., & Gordo, L. R. (2018). Gender, age and migration: An intersectional approach to inequalities in the labour market. *European Journal of Ageing*, 15(1), 23-33. https://doi.org/10.1007/s10433-017-0419-2
578. Sunil, T. S., & Xu, X. (2019). Substance abuse and HIV/STD prevention at a Hispanic-serving institution in south Texas: A study of racial/ethnic and gender heterogeneity and intersectionality. *Journal of Ethnicity in Substance Abuse,* 1-18. https://doi.org/10.1080/15332640.2019.1641173
579. Sutton, A., Langenkamp, A. G., Muller, C., & Schiller, K. S. (2018). Who gets ahead and who falls behind during the transition to high school? Academic performance at the intersection of race/ethnicity and gender. *Social Problems*, 65(2), 154-173. https://doi.org/10.1093/socpro/spx044
580. Swain, K. E. O., & Lien, P.-T. (2017). Structural and contextual factors regarding the accessibility of elective office for women of color at the local level. *Journal of Women, Politics & Policy*, 38(2) 128–150. https://doi.org/10.1080/1554477X.2016.1198653
581. Swank, E. (2018). Who voted for Hillary Clinton? Sexual identities, gender, and family influences. *Journal of GLBT Family Studies*, 14(1-2), 21–42. https://doi.org/10.1080/1550428X.2017.1421335
582. Swank, E., & Fahs, B. (2013). An intersectional analysis of gender and race for sexual minorities who engage in gay and lesbian rights activism. *Sex Roles*, 68(11), 660-674. https://doi.org/10.1007/s11199-012-0168-9
583. Sweetman, J. (2018). When similarities are more important than differences: "Politically black" union members' experiences of racism and participation in union leadership. *Journal of Social Issues*, 74(2), 244–264. https://doi.org/10.1111/josi.12267

584. Swendener, A., & Woodell, B. (2017). Predictors of family support and well-being among black and latina/o sexual minorities. *Journal of GLBT Family Studies*, 13(4), 357–379. https://doi.org/10.1080/1550428X.2016.1257400
585. Syed, J. (2007). "The other woman" and the question of equal opportunity in Australian organizations. *International Journal of Human Resource Management*, 18(11), 1954–1978. https://doi.org/10.1080/09585190701638184
586. Szmer, J, Christensen, R. K., & Kaheny, E. B. (2015). Gender, race, and dissensus on state supreme courts. *Social Science Quarterly*, 96(2), 553–575. https://doi.org/10.1111/ssqu.12133
587. Taggart, T., Powell, W., Gottfredson, N., Ennett, S., Eng, E., & Chatters, L. M. (2019). A person-centered approach to the study of Black adolescent religiosity, racial identity, and sexual initiation. *Journal of Research on Adolescence*, 29(2), 402–413. https://doi.org/10.1111/jora.12445
588. Tan, A. S. L., Hanby, E. P., Sanders-Jackson, A., Lee, S., Viswanath, K., & Potter, J. (2019). Inequities in tobacco advertising exposure among young adult sexual, racial and ethnic minorities: Examining intersectionality of sexual orientation with race and ethnicity. *Tobacco control*, 30(1), 84-93. https://doi.org/10.1136/tobaccocontrol-2019-055313
589. Tao, C., Scott, K. A., & McCarthy, K. S. (2020). Do African American male and female adolescents differ in technological engagement?: The effects of parental encouragement and adolescent technological confidence. *Sex Roles*, 83, 536–551. https://doi.org/10.1007/s11199-020-01134-0
590. Tao, Y. (2018). Earnings of academic scientists and engineers: Intersectionality of gender and race/ethnicity effects. *American Behavioral Scientist*, 62(5), 625-644. https://doi.org/10.1177/0002764218768870
591. Tao, Y. (2020). Earnings of foreign-born doctoral engineers in the United States: Intersectionality of citizenship status and gender. *Sociological Spectrum*, 40(3), 151-171. https://doi.org/10.1080/02732173.2020.1732250
592. Taylor, T., & Risman, B. J. (2006). Doing deference or speaking up: Deconstructing the experience and expression of anger. *Race, Gender & Class*, 13(3-4), 60–80. https://www.jstor.org/stable/41675173
593. Tejera, C. H., Horner-Johnson, W., & Andresen, E. M. (2019). Application of an intersectional framework to understanding the association of disability and sexual orientation with suicidal ideation among Oregon Teens. *Disability and Health Journal*, *12*(4), 557–563. https://doi.org/10.1016/j.dhjo.2019.05.006
594. Terriquez, V. (2015). Intersectional mobilization, social movement spillover, and queer youth leadership in the immigrant rights movement. *Social Problems*, 62(3), 343-362. https://doi.org/10.1093/socpro/spv010
595. Tezcür, G. M. (2019). A path out of patriarchy? Political agency and social identity of women fighters. *Perspectives on Politics*, 18(3), 722-739. https://doi.org/10.1017/S1537592719000288
596. Theobald, H. (2017). Care workers with migration backgrounds in formal care services in Germany: A multi-level intersectional analysis. *International Journal of Care and Caring*, 1(2), 209–226. https://doi.org/10.1332/239788217X14944099147786

597. Thomas, M. (2018). "It's hardly fair to bring a child into the world with the way things look…": Anomie, mistrust, and the impact of race, SES, and gender. *Sociological Inquiry*, 88(2), 254–273. https://doi.org/10.1111/soin.12191
598. Tichenor, V., McQuillan, J., Greil, A. L., Bedrous, A. V, Clark, A., & Shreffler, K. M. (2017). Variation in attitudes toward being a mother by race/ethnicity and education among women in the United States. *Sociological Perspectives*, 60(3), 600–619. https://doi.org/10.1177/0731121416662452
599. Todd, N. R., McConnell, E. A., & Suffrin, R. L. (2014). The role of attitudes toward white privilege and religious beliefs in predicting social justice interest and commitment. *American Journal of Community Psychology*, 53(1-2), 109–121. https://doi.org/10.1007/s10464-014-9630-x
600. Tomlinson, J., Valizade, D., Muzio, D., Charlwood, A., & Aulakh, S. (2019). Privileges and penalties in the legal profession: An intersectional analysis of career progression. *The British Journal of Sociology*, 70(3), 1043–1066. https://doi.org/10.1111/1468-4446.12375
601. Toosi, N. R., Babbitt, L. G., Ambady, N., & Sommers, S. R. (2012). Dyadic interracial interactions: A meta-analysis. *Psychological Bulletin*, 138(1), 1–27. https://doi.org/10.1037/a0025767
602. Toosi, N. R., Mor, S., Semnani-Azad, Z., Phillips, K. W., & Amanatullah, E. T. (2019). Who can lean in? The intersecting role of race and gender in negotiations. *Psychology of Women Quarterly*, 43(1), 7–21. https://doi.org/10.1177/0361684318800492
603. Torres Stone, R. A., Purkayastha, B., & Berdahl, T. A. (2006). Beyond Asian American: Examining conditions and mechanisms of earnings inequality for Filipina and Asian Indian women. *Sociological Perspectives*, 49(2), 261–281. https://doi.org/10.1525/sop.2006.49.2.261
604. Trahan, A., Dixon, A., & Nodeland, B. (2019). Public opinion of capital punishment: An intersectional analysis of race, gender, and class effects. *Criminal Justice Review*, 44(4), 452–469. https://doi.org/10.1177/0734016818818687
605. Tuthill, Z.O., & Gorman, B. (2019). Medical comfort at the intersection of sexual orientation and gender identity. *Social Currents*, 6(5), 464–486. https://doi.org/10.1177/2329496519852580
606. Twis, M. K. (2019). Predicting different types of victim-trafficker relationships: A multinomial logistic regression analysis. *Journal of Human Trafficking*, 6(4), 450-466. https://doi.org/10.1080/23322705.2019.1634963
607. Uddin, J., Acharya, S., Valles, J., Baker, E. H., & Keith, V. M. (2020). Caste differences in hypertension among women in India: Diminishing health returns to socioeconomic status for lower caste groups. *Journal of Racial and Ethnic Health Disparities*, 7, 987–995. https://doi.org/10.1007/s40615-020-00723-9
608. Uhlaner, C. J., & Scola, B. (2016). Collective representation as a mobilizer: Race/ethnicity, gender, and their intersections at the state level. *State Politics and Policy Quarterly*, 16(2), 227–263. https://doi.org/10.1177/1532440015603576
609. Uyheng, J., Nicdao, J. V, Carmona, C. L., & Canoy, N. A. (2020). Intersectional discourses of reproductive agency in the Philippines: A mixed methods analysis of classed constructions of pregnancy resolution. *Feminism & Psychology*, 30(4), 445-468. https://doi.org/10.1177/0959353520915829

610. Uzogara, E. E. (2019). Gendered racism biases: Associations of phenotypes with discrimination and internalized oppression among Latinx American women and men. *Race and Social Problems*, 11(1), 80–92. https://doi.org/10.1007/s12552-018-9255-z
611. Valdez, Z. (2015), "Intersectional Differences in Segmented Assimilation: Skill and Gender in the Context of Reception", Immigration and Work (Research in the Sociology of Work, Vol. 27), Emerald Group Publishing Limited, pp. 101-128. https://doi.org/10.1108/S0277-283320150000027012
612. Valiente, O., & Rambla, X. (2009). The new other Catalans at school: Decreasing unevenness but increasing isolation. *International Studies in Sociology of Education*, 19(2), 105–117. https://doi.org/10.1080/09620210903257190
613. Van Aelst, K., & Holvoet, N. (2016). Intersections of gender and marital status in accessing climate change adaptation: Evidence from Rural Tanzania. *World Development*, 79, 40–50. https://doi.org/10.1016/j.worlddev.2015.11.003
614. Van De Werfhorst, H. G. (2017). Gender segregation across fields of study in post-secondary education: Trends and social differentials. *European Sociological Review*, 33(3), 449–464. https://doi.org/10.1093/esr/jcx040
615. Van Dusen, B., & Nissen, J. (2020). Equity in college physics student learning: A critical quantitative intersectionality investigation. *Journal of Research in Science Teaching*, 57(1), 33–57. https://doi.org/10.1002/tea.21584
616. Van Dyke, M. E., Vaccarino, V., Dunbar, S. B., Pemu, P., Gibbons, G. H., Quyyumi, A. A., & Lewis, T. T. (2017). Socioeconomic status discrimination and C-reactive protein in African-American and White adults. *Psychoneuroendocrinology*, 82, 9-16. https://doi.org/10.1016/j.psyneuen.2017.04.009
617. Vargas, J C, & Alves, J. A. (2010). Geographies of death: An intersectional analysis of police lethality and the racialized regimes of citizenship in São Paulo. *Ethnic and Racial Studies*, 33(4), 611–636. https://doi.org/10.1080/01419870903325636
618. Vedam, S., Stoll, K., Taiwo, T. K., Rubashkin, N., Cheyney, M., Strauss, N., McLemore, M., Cadena, M., Nethery, E., Rushton, E., Schummers, L., & Declercq, E. (2019). The Giving Voice to Mothers study: Inequity and mistreatment during pregnancy and childbirth in the United States. *Reproductive Health*, 16, 77. https://doi.org/10.1186/s12978-019-0729-2
619. Veenstra, G. (2011). Race, gender, class, and sexual orientation: Intersecting axes of inequality and self-rated health in Canada. *International Journal for Equity in Health*, 10, 3. https://doi.org/10.1186/1475-9276-10-3
620. Veenstra, G. (2013a). Race, Gender, Class, Sexuality (RGCS) and hypertension. *Social Science and Medicine*, 89, 16–24. https://doi.org/10.1016/j.socscimed.2013.04.014
621. Veenstra, G. (2013b). The gendered nature of discriminatory experiences by race, class, and sexuality: a comparison of intersectionality theory and the subordinate male target hypothesis. *Sex Roles*, 68(11-12), 646–659. https://doi.org/10.1007/s11199-012-0243-2
622. Veenstra, G., & Patterson, A. C. (2016). South Asian-White health inequalities in Canada: intersections with gender and immigrant status. *Ethnicity and Health*, 21(6), 639–648. https://doi.org/10.1080/13557858.2016.1179725
623. Veldhuis, C. B., Hughes, T. L., Drabble, L. A., Wilsnack, S. C., & Matthews, A. K. (2020). Do relationships provide the same levels of protection against heavy drinking for lesbian and bisexual women? An intersectional approach. *Psychology of Sexual Orientation and Gender Diversity*, 7(3), 337–352. https://doi.org/10.1037/sgd0000383

624. Velez, B. L., Cox Jr., R., Polihronakis, C. J., & Moradi, B. (2018). Discrimination, work outcomes, and mental health among women of color: The protective role of womanist attitudes. *Journal of Counseling Psychology*, 65(2), 178-193. https://doi.org/10.1037/cou0000274
625. Vespa, J. (2009). Gender ideology construction: A life course and intersectional approach. *Gender and Society*, 23(3), 363–387. https://doi.org/10.1177/0891243209337507
626. Vigna, A. J., Poehlmann-Tynan, J., & Koenig, B. W. (2018). Does self-compassion covary with minority stress? Examining group differences at the intersection of marginalized identities. *Self and Identity*, 17(6), 687–709. https://doi.org/10.1080/15298868.2018.1457566
627. Vigna, A. J., Poehlmann-Tynan, J., & Koenig, B. W. (2020). Is self-compassion protective among sexual- and gender-minority adolescents across racial groups? *Mindfulness*, 11(3), 800–815. https://doi.org/10.1007/s12671-019-01294-5
628. Villanti, A. C., Gaalema, D. E., Tidey, J. W., Kurti, A. N., Sigmon, S. C., & Higgins, S. T. (2018). Co-occurring vulnerabilities and menthol use in U.S. young adult cigarette smokers: Findings from Wave 1 of the PATH Study, 2013–2014. *Preventive Medicine*, 117, 43–51. https://doi.org/10.1016/j.ypmed.2018.06.001
629. Villanueva, I., Carothers, T., Di Stefano, M., & Khan, M. T. H. (2018). "There is never a break": The hidden curriculum of professionalization for engineering Faculty. *Education Sciences*, 8(4), 157. https://doi.org/10.3390/educsci8040157
630. Villatoro, A. P., Mays, V. M., Ponce, N. A., & Aneshensel, C. S. (2018). Perceived need for mental health care: The intersection of race, ethnicity, gender, and socioeconomic status. *Society and Mental Health*, 8(1), 1–24. https://doi.org/10.1177/2156869317718889
631. Vu, M., Li, J., Haardörfer, R., Windle, M., & Berg, C. J. (2019). Mental health and substance use among women and men at the intersections of identities and experiences of discrimination: Insights from the intersectionality framework. *BMC Public Health*, 19(1), 108. https://doi.org/10.1186/s12889-019-6430-0
632. Walker, J. J., Longmire-Avital, B., & Golub, S. (2015). Racial and sexual identities as potential buffers to risky sexual behavior for Black gay and bisexual emerging adult men. *Health Psychology*, 34(8), 841–846. https://doi.org/10.1037/hea0000187
633. Walls, N. E., Atteberry-Ash, B., Kattari, S. K., Peitzmeier, S., Kattari, L., & Langenderfer-Magruder, L. (2019). Gender identity, sexual orientation, mental health, and bullying as predictors of partner violence in a representative sample of youth. *Journal of Adolescent Health*, 64(1), 86–92. https://doi.org/10.1016/j.jadohealth.2018.08.011
634. Walsemann, K. M., Goosby, B. J., & Farr, D. (2016). Life course SES and cardiovascular risk: Heterogeneity across race/ethnicity and gender. *Social Science and Medicine*, 152, 147–155. https://doi.org/10.1016/j.socscimed.2016.01.038
635. Walther, C. S. (2014). Skin tone, biracial stratification and tri-racial stratification among sperm donors. *Ethnic and Racial Studies*, 37(3), 517–536. https://doi.org/10.1080/01419870.2012.696666
636. Wang, Q. (2019). Gender, race/ethnicity, and entrepreneurship: Women entrepreneurs in a US South city. *International Journal of Entrepreneurial Behavior & Research*, 25(8), 1766–1785. https://doi.org/10.1108/IJEBR-05-2017-0156

637. Wang, Y., Cheng, C., & Bian, Y. (2018). More than double jeopardy: An intersectional analysis of persistent income disadvantages of Chinese female migrant workers. *Asian Journal of Women's Studies*, 24(2), 246–269. https://doi.org/10.1080/12259276.2018.1469722
638. Wanka, A., Wiesböck, L., Allex, B., Mayrhuber, E. A.-S., Arnberger, A., Eder, R., Kutalek, R., Wallner, P., Hutter, H.-P., & Kolland, F. (2019). Everyday discrimination in the neighbourhood: What a "doing" perspective on age and ethnicity can offer. *Ageing & Society*, 39(9), 2133–2158. https://doi.org/10.1017/S0144686X18000466
639. Warner, D. F., & Brown, T. H. (2011). Understanding how race/ethnicity and gender define age-trajectories of disability: An intersectionality approach. *Social Science and Medicine*, 72(8), 1236–1248. https://doi.org/10.1016/j.socscimed.2011.02.034
640. Watson, L. B., DeBlaere, C., Langrehr, K. J., Zelaya, D. G., & Flores, M. J. (2016). The influence of multiple oppressions on women of color's experiences with insidious trauma. *Journal of Counseling Psychology*, 63(6), 656–667. https://doi.org/10.1037/cou0000165
641. Watson, L B, Morgan, S. K., & Craney, R. (2018). Bisexual women's discrimination and mental health outcomes: The roles of resilience and collective action. *Psychology of Sexual Orientation and Gender Diversity*, 5(2), 182–193. https://doi.org/10.1037/sgd0000272
642. Wechsberg, W. M., Zule, W. A., Luseno, W. K., Kline, T. L., Browne, F. A., Novak, S. P., & Ellerson, R. M. (2011). Effectiveness of an adapted evidence-based woman-focused intervention for sex workers and non-sex workers: The women's health co-op in South Africa. J*ournal of Drug Issues*, 41(2), 233–252. https://doi.org/10.1177/002204261104100205
643. Weedon, E. (2017). The construction of under-representation in UK and Swedish higher education: Implications for disabled students. *Education, Citizenship and Social Justice*, 12(1), 75–88. https://doi.org/10.1177/1746197916683470
644. Wemrell, M., Bennet, L., & Merlo, J. (2019). Understanding the complexity of socioeconomic disparities in type 2 diabetes risk: A study of 4.3 million people in Sweden. *BMJ Open Diabetes Research and Care*, 7(1), e000749. https://doi.org/10.1136/bmjdrc-2019-000749
645. Wemrell, M., Mulinari, S., & Merlo, J. (2017). Intersectionality and risk for ischemic heart disease in Sweden: Categorical and anti-categorical approaches. *Social Science and Medicine*, 177, 213–222. https://doi.org/10.1016/j.socscimed.2017.01.050
646. Weston, R. E., Zeng, H., & Battle, J. (2020). Physical activity and GPA: Results from a national sample of Black students. *Journal of Human Behavior in the Social Environment*, 30(4), 383–398. https://doi.org/10.1080/10911359.2019.1687387
647. Whaley, A. L., & Dubose, J. (2018). Intersectionality of ethnicity/race and gender in the phenomenology of African American college students' presenting problems: A Profile analysis using nonmetric multidimensional scaling. *International Journal for the Advancement of Counselling*, 40(3), 279–297. https://doi.org/10.1007/s10447-018-9326-2
648. Wheldon, C. W., Watson, R. J., Fish, J. N., & Gamarel, K. (2019). Cigarette smoking among youth at the intersection of sexual orientation and gender identity. *LGBT Health*, 6(5), 235–241. https://doi.org/10.1089/lgbt.2019.0005

649. Whitman, C. N., & Nadal, K. L. (2015). Sexual minority identities: Outness and well-being among lesbian, gay, and bisexual adults. *Journal of Gay and Lesbian Mental Health*, 19(4), 370–396. https://doi.org/10.1080/19359705.2015.1038974
650. Willert, B., & Minnotte, K. L. (2019). Informal caregiving and strains: Exploring the impacts of gender, race, and income. *Applied Research in Quality of Life*, 1-22. https://doi.org/10.1007/s11482-019-09786-1
651. Williams, C C, Curling, D., Steele, L. S., Gibson, M. F., Daley, A., Green, D. C., & Ross, L. E. (2017). Depression and discrimination in the lives of women, transgender and gender liminal people in Ontario, Canada. *Health and Social Care in the Community*, 25(3), 1139–1150. https://doi.org/10.1111/hsc.12414
652. Williams, D. T., Simon, L., & Cardwell, M. (2019). Black intimacies matter: The role of family status, gender, and cumulative risk on relationship quality among Black parents. *Journal of African American Studies*, 23(1-2). https://doi.org/10.1007/s12111-019-09420-2
653. Williams, M. G., & Lewis, J. A. (2019). Gendered racial microaggressions and depressive symptoms among black Women: A moderated mediation model. *Psychology of Women Quarterly*, 43(3), 368–380. https://doi.org/10.1177/0361684319832511
654. Williamson, T. J., Mahmood, Z., Kuhn, T. P., & Thames, A. D. (2017). Differential relationships between social adversity and depressive symptoms by HIV status and racial/ethnic identity. *Health Psychology*, 36(2), 133–142. https://doi.org/10.1037/hea0000458
655. Winkler, M. R., Telke, S., Ahonen, E. Q., Crane, M. M., Mason, S. M., & Neumark-Sztainer, D. (2020). Constrained choices: Combined influences of work, social circumstances, and social location on time-dependent health behaviors. *SSM - Population Health*, 11, 100562. https://doi.org/10.1016/j.ssmph.2020.100562
656. Wittenberg, Y., de Boer, A., Plaisier, I., Verhoeff, A., & Kwekkeboom, R. (2019). Informal caregivers' judgements on sharing care with home care professionals from an intersectional perspective: The influence of personal and situational characteristics. *Scandinavian Journal of Caring Sciences*, 33(4), 1006–1016. https://doi.org/10.1111/scs.12699
657. Wong-Padoongpatt, G., Zane, N., Okazaki, S., & Saw, A. (2020). Individual variations in stress response to racial microaggressions among Asian Americans. *Asian American Journal of Psychology*, 11(3), 126–137. https://doi.org/10.1037/aap0000182
658. Woodhams, C., Lupton, B., & Cowling, M. (2015). The presence of ethnic minority and disabled men in feminised work: Intersectionality, vertical segregation and the glass escalator. *Sex Roles*, 72(7-8), 277–293. https://doi.org/10.1007/s11199-014-0427-z
659. Worthen, M. G. F. (2018). "Gay equals white"? Racial, ethnic, and sexual identities and attitudes toward LGBT Individuals among college students at a bible belt university. *The Journal of Sex Research*, 55(8), 995–1011. https://doi.org/10.1080/00224499.2017.1378309
660. Worthen, M. G. F., & Wallace, S. A. (2017). Intersectionality and perceptions about sexual assault education and reporting on college campuses. *Family Relations*, 66(1), 180–196. https://doi.org/10.1111/fare.12240
661. Yang, X., Li, X., Qiao, S., Li, L., Parker, C., Shen, Z., & Zhou, Y. (2020). Intersectional stigma and psychosocial well-being among MSM living with HIV in

Guangxi, China. *AIDS Care*, 32(sup2), 5–13. https://doi.org/10.1080/09540121.2020.1739205
662. Yap, S. C. Y., Settles, I. H., & Pratt-Hyatt, J. S. (2011). Mediators of the relationship between racial identity and life satisfaction in a community sample of African American women and men. *Cultural Diversity and Ethnic Minority Psychology,* 17(1), 89–97. https://doi.org/10.1037/a0022535
663. Yasun, S. (2018). Does education enable underprivileged women to achieve real equality in property rights? A case study of inheritance rights of women in Turkey. *Women's Studies International Forum*, 69, 100–114. https://doi.org/10.1016/j.wsif.2018.05.013
664. Yaussy, S. L. (2019). The intersections of industrialization: Variation in skeletal indicators of frailty by age, sex, and socioeconomic status in 18th- and 19th-century England. *American Journal of Physical Anthropology,* 170(1), 116–130. https://doi.org/10.1002/ajpa.23881
665. Young, J. L., Young, J. R., & Capraro, R. M. (2018). Gazing past the gaps: a growth-based assessment of the mathematics achievement of Black girls. *The Urban Review,* 50(1), 156–176. https://doi.org/10.1007/s11256-017-0434-9
666. Young, J. L., Foster, M. D., & Druery, D. M. (2018). A critical exploratory analysis of Black girls' achievement in 8th grade U.S. history. *Middle Grades Review,* 4(3), 1–16. https://scholarworks.uvm.edu/mgreview/vol4/iss3/2
667. Yu, H. H. (2020). Multiracial feminism: An intersectional approach to examining female officers' occupational barriers in federal law enforcement. *Women & Criminal Justice,* 1-15. https://doi.org/10.1080/08974454.2020.1734146
668. Zaykowski, H., Allain, E. C., & Campagna, L. M. (2019). Examining the paradox of crime reporting: Are disadvantaged victims more likely to report to the police? *Law & Society Review*, 53(4), 1305–1340. https://doi.org/10.1111/lasr.12440
669. Zimmermann, C. R., & Kao, G. (2020). Unequal returns to children's efforts. *Du Bois Review,* 16(2), 417-438. https://doi.org/10.1017/S1742058X20000016
670. Zuccotti, C. V, & O'Reilly, J. (2019). Ethnicity, gender and household effects on becoming NEET: An intersectional analysis. *Work, Employment and Society,* 33(3), 351–373. https://journals.sagepub.com/doi/full/10.1177/0950017017738945
671. Zurbrügg, L., & Miner, K. N. (2016). Gender, sexual orientation, and workplace incivility: Who is most targeted and who is most harmed? *Frontiers in Psychology*, 7, 565. https://doi.org/10.3389/fpsyg.2016.00565

B.4. Bibliography: Methods papers for quantitative intersectionality that included an original data analysis application

1. Bauer, G. R., & Scheim, A. I. (2019). Methods for analytic intercategorical intersectionality in quantitative research: Discrimination as a mediator of health inequalities. *Social Science & Medicine, 226,* 236-245. https://doi.org/10.1016/j.socscimed.2018.12.015
2. Evans, C. R., Williams, D. R., Onnela, J. P., & Subramanian, S. V. (2018). A multilevel approach to modeling health inequalities at the intersection of multiple social identities.

Social Science & Medicine, 203, 64-73.
https://doi.org/10.1016/j.socscimed.2017.11.011

3. Evans, C. R. (2019). Adding interactions to models of intersectional health inequalities: Comparing multilevel and conventional methods. *Social Science & Medicine, 221*, 95-105. https://doi.org/10.1016/j.socscimed.2018.11.036
4. Evans, C. R., Leckie, G., & Merlo, J. (2020). Multilevel versus single-level regression for the analysis of multilevel information: The case of quantitative intersectional analysis. *Social Science & Medicine, 245*, 112499. https://doi.org/10.1016/j.socscimed.2019.112499
5. Evans, C. R. (2019). Reintegrating contexts into quantitative intersectional analyses of health inequalities. *Health & Place*, *60*, 102214. https://doi.org/10.1016/j.healthplace.2019.102214
6. Guinea-Martin, D., Mora, R., & Ruiz-Castillo, J. (2015). The joint effect of ethnicity and gender on occupational segregation. An approach based on the mutual information index. *Social Science Research, 49,* 167-178. https://doi.org/10.1016/j.ssresearch.2014.08.007
7. Jackson, J. W., Williams, D. R., & VanderWeele, T. J. (2016). Disparities at the intersection of marginalized groups. *Social Psychiatry and Psychiatric Epidemiology, 51*(10), 1349-1359. https://doi.org/10.1007/s00127-016-1276-6
8. Scott, N. A., & Siltanen, J. (2017). Intersectionality and quantitative methods: Assessing regression from a feminist perspective. *International Journal of Social Research Methodology, 20*(4), 373-385. https://doi.org/10.1080/13645579.2016.1201328 [excluded from analyses on application papers, as used only hypothetical or published data]
9. Sen, G., Iyer, A., & Mukherjee, C. (2009). A methodology to analyse the intersections of social inequalities in health. *Journal of Human Development and Capabilities, 10*(3), 397-415. https://doi.org/10.1080/19452820903048894
10. Stirratt, M. J., Meyer, I. H., Ouellette, S. C., & Gara, M. A. (2008). Measuring identity multiplicity and intersectionality: Hierarchical classes analysis (HICLAS) of sexual, racial, and gender identities. *Self and Identity*, *7*(1), 89-111. https://doi.org/10.1080/15298860701252203
11. Ohman, S., Olofsson, A., Giritli, N. K. (2018) A methodological strategy for exploring intersecting inequalities: An example from Sweden. *The Journal of Social Policy Studies, 16*(3), 501-516. https://doi.org/10.17323/727-0634-2018-16-3-501-516

B.5. Bibliography: Methods-only papers for quantitative intersectionality

1. McCall, L. (2005). The complexity of intersectionality. *Signs: Journal of Women in Culture and Society*, *30*(3), 1771-1800. https://doi.org/10.1086/426800 [not included in the 707 papers in the systematic review, but added as one of 45 potential methods papers cited]
2. Weldon, S. L. (2006). The structure of intersectionality: A comparative politics of gender. *Politics & Gender*, *2*(2), 235 -248. https://doi.org/10.1017/S1743923X06231040

3. Hancock, A. M. (2007). When multiplication doesn't equal quick addition: Examining intersectionality as a research paradigm. *Perspectives on Politics, 5*(1), 63-79. https://doi.org/10.1017/S1537592707070065 [not included in the 707 papers in the systematic review, but added as one of 45 potential methods papers cited]
4. Bowleg, L. (2008). When Black+ lesbian+ woman≠ Black lesbian woman: The methodological challenges of qualitative and quantitative intersectionality research. *Sex Roles*, *59*(5), 312-325. https://doi.org/10.1007/s11199-008-9400-z [not included in the 707 papers in the systematic review, but added as one of 45 potential methods papers cited]
5. Nash, J. C. (2008). Re-thinking intersectionality. *Feminist Review*, *89*(1), 1-15. https://doi.org/10.1057/fr.2008.4 [not included in the 707 papers in the systematic review, but added as one of 45 potential methods papers cited]
6. Shields, S. A. (2008). Gender: An intersectionality perspective. *Sex Roles, 59*(5), 301-311. https://doi.org/10.1007/s11199-008-9501-8 [not included in the 707 papers in the systematic review, but added as one of 45 potential methods papers cited]
7. Warner, L. R. (2008). A best practices guide to intersectional approaches in psychological research. *Sex Roles*, *59*(5), 454-463. https://doi.org/10.1007/s11199-008-9504-5
8. Cole, E. R. (2009). Intersectionality and research in psychology. A*merican Psychologist, 64*(3), 170 -180. https://doi.org/10.1037/a0014564 [not included in the 707 papers in the systematic review, but added as one of 45 potential methods papers cited]
9. Bowleg, L. (2012). The problem with the phrase women and minorities: Intersectionality—an important theoretical framework for public health. *American Journal of Public Health, 10*2(7), 1267-1273. https://doi.org/10.2105/AJPH.2012.300750 [not included in the 707 papers in the systematic review, but added as one of 45 potential methods papers cited]
10. Bauer, G. R. (2014). Incorporating intersectionality theory into population health research methodology: Challenges and the potential to advance health equity. *Social Science & Medicine*, *110*, 10-17. https://doi.org/10.1016/j.socscimed.2014.03.022
11. Bowleg, L., & Bauer, G. (2016). Invited reflection: Quantifying intersectionality. *Psychology of Women Quarterly*, *40*(3), 337-341. https://doi.org/10.1177/0361684316654282
12. Bright, L. K., Malinsky, D., & Thompson, M. (2016). Causally interpreting intersectionality theory. *Philosophy of Science*, *83*(1), 60-81. https://doi.org/10.1086/684173
13. Del Toro, J., & Yoshikawa, H. (2016). Invited reflection: Intersectionality in quantitative and qualitative research. *Psychology of Women Quarterly*, *40*(3), 347-350. https://doi.org/10.1177/0361684316655768 [not included in the 707 papers in the systematic review, but added as one of 45 potential methods papers cited]
14. Else-Quest, N. M., & Hyde, J. S. (2016). Intersectionality in quantitative psychological research: I. Theoretical and epistemological issues. *Psychology of Women Quarterly*, *40*(2), 155-170. https://doi.org/10.1177/0361684316629797
15. Else-Quest, N. M., & Hyde, J. S. (2016). Intersectionality in quantitative psychological research: II. Methods and techniques. *Psychology of Women Quarterly, 40*(3), 319-336. https://doi.org/10.1177/0361684316647953

16. Else-Quest, N. M., & Hyde, J. S. (2016). The authors respond: Continuing the dialectic on intersectionality in psychology. *Psychology of Women Quarterly, 40*(3), 351-352. https://doi.org/10.1177/0361684316655757
17. Else-Quest, N. M., & Hyde, J. S. (2016). The authors respond: Intersectionality theory and epistemology. *Psychology of Women Quarterly, 40*(2), 182-183. https://doi.org/10.1177/0361684316641054
18. Marecek, J. (2016). Invited reflection: Intersectionality theory and feminist psychology. *Psychology of Women Quarterly, 40*(2), 177-181. https://doi.org/10.1177/0361684316641090
19. Warner, L. R. (2016). Invited reflection: Contested interpretations and methodological choices in quantitative research. *Psychology of Women Quarterly, 40*(3), 342-346. https://doi.org/10.1177/0361684316655453
20. Warner, L. R., Settles, I. H., & Shields, S. A. (2016). Invited reflection: Intersectionality as an epistemological challenge to psychology. *Psychology of Women Quarterly, 40*(2), 171-176. https://doi.org/10.1177/0361684316641384
21. Green, M. A., Evans, C. R., & Subramanian, S.V. (2017). Can intersectionality theory enrich population health research? *Social Science & Medicine, 178*, 214–216. https://doi.org/10.1016/j.socscimed.2017.02.029
22. Jackson, J. W. (2017). Explaining intersectionality through description, counterfactual thinking, and mediation analysis. *Social Psychiatry and Psychiatric Epidemiology, 52*(7), 785-793. https://doi.org/10.1007/s00127-017-1390-0
23. Schwartz, S. (2017). Commentary: On the application of potential outcomes-based methods to questions in social psychiatry and psychiatric epidemiology. *Social Psychiatry and Psychiatric Epidemiology, 52*(2), 139-142. https://doi.org/10.1007/s00127-016-1334-0 [not included in the 707 papers in the systematic review, but added as one of 45 potential methods papers cited]
24. Wemrell, M., Mulinari, S., & Merlo, J. (2017). An intersectional approach to multilevel analysis of individual heterogeneity (MAIH) and discriminatory accuracy. *Social Science and Medicine, 178*, 217-219. https://doi.org/10.1016/j.socscimed.2017.02.040
25. Merlo, J. (2018). Multilevel analysis of individual heterogeneity and discriminatory accuracy (MAIHDA) within an intersectional framework. *Social Science & Medicine, 203*, 74-80. https://doi.org/10.1016/j.socscimed.2017.12.026
26. Schudde, L. (2018). Heterogeneous effects in education: The promise and challenge of incorporating intersectionality into quantitative methodological approaches. *Review of Research in Education, 42*(1), 72-92. https://doi.org/10.3102/0091732X18759040
27. Bailey, J., Steeves, V., Burkell, J., Shade, L. R., Ruparelia, R., & Regan, P. (2019). Getting at equality: Research methods informed by the lessons of intersectionality. *International Journal of Qualitative Methods, 18*, 1609406919846753. https://doi.org/10.1177/1609406919846753
28. Bauer, G. R., & Scheim, A. I. (2019). Advancing quantitative intersectionality research methods: Intracategorical and intercategorical approaches to shared and differential constructs. *Social Science & Medicine, 226*, 260-262. https://doi.org/10.1016/j.socscimed.2019.03.018
29. Bell, A., Holman, D., & Jones, K. (2019) Using shrinkage in multilevel models to understand intersectionality. *Methodology, 15*(2), 88-96. https://doi.org/10.1027/1614-2241/a000167

30. Evans, C. R. (2019). Modeling the intersectionality of processes in the social production of health inequalities. *Social Science & Medicine, 226,* 249-253. https://doi.org/10.1016/j.socscimed.2019.01.017
31. Harnois, C. E., & Bastos, J. L. (2019). The promise and pitfalls of intersectional scale development. *Social Science & Medicine*, *223*, 73–76. https://doi.org/10.1016/j.socscimed.2019.01.039
32. Jackson, J. W., & VanderWeele, T. J. (2019). Intersectional decomposition analysis with differential exposure, effects, and construct. *Social Science & Medicine, 226,* 254-259. https://doi.org/10.1016/j.socscimed.2019.01.033
33. Richman, L. S., & Zucker, A. N. (2019). Quantifying intersectionality: An important advancement for health inequality research. *Social Science & Medicine, 226*, 246-248. https://doi.org/10.1016/j.socscimed.2019.01.036
34. Lizotte, D. J., Mahendran, M., Churchill, S. M., & Bauer, G. R. (2020). Math versus meaning in MAIHDA: A commentary on multilevel statistical models for quantitative intersectionality. *Social Science & Medicine*, *245*, 112500. https://doi.org/10.1016/j.socscimed.2019.112500

APPENDIX C. JOURNAL DISCIPLINE CATEGORIES

Our Subject Categories	Ulrich's Discipline Categories
BUSINESS AND ECONOMICS	BUSINESS AND ECONOMICS
CHILDREN AND YOUTH	CHILDREN AND YOUTH
DISABILITY	HANDICAPPED
EDUCATION	EDUCATION
ETHNIC STUDIES	ETHNIC INTERESTS
GENDER AND SEXUALITY STUDIES	MEN'S STUDIES<br>WOMEN'S INTERESTS<br>HOMOSEXUALITY<br>WOMEN'S STUDIES<br>WOMEN'S HEALTH<br>MEN'S HEALTH
HISTORY	HISTORY
HUMANITIES	HUMANITIES
LAW & CRIMINOLOGY	CRIMINOLOGY AND LAW ENFORCEMENT<br>LAW
MEDICINE & LIFE SCIENCES	MEDICAL SCIENCES<br>GERONTOLOGY AND GERIATRICES<br>DRUG ABUSE AND ALCOHOLISM<br>GERONTOLOGY AND GERIATRICS<br>NUTRITION AND DIETETICS<br>HEALTH FACILITIES AND ADMINISTRATION<br>PHARMACY AND PHARMACOLOGY<br>MEDICAL SCIENCE<br>BIOLOGY<br>DRUG ABUSE AND ALCOHOLISM<br>TOBACCO<br>PHYSICAL FITNESS AND HYGIENE
OTHER SCIENCES	COMPUTERS<br>SCIENCES<br>TECHNOLOGY
OTHER SOCIAL SCIENCES	LIFESTYLE<br>ANTHROPOLOGY<br>MATRIMONY<br>SOCIAL SCIENCES<br>SOCIAL SERVICES AND WELFARE<br>OCCUPATIONS AND CAREERS
PHILOSOPHY & RELIGION	RELIGIONS AND THEOLOGY<br>PHILOSOPHY
PHYSICAL, EARTH & SPACE SCIENCES	METEOROLOGY<br>AERONAUTICS AND SPACE FLIGHT<br>WATER RESOURCES<br>GEOGRAPHY<br>ENVIRONMENTAL STUDIES

	PHYSICS<br>ENGINEERING<br>ENERGY<br>PETROLEUM AND GAS
POLITICAL SCIENCE	POLITICAL SCIENCE<br>CIVIL DEFENSE
POPULATION/PUBLIC HEALTH & SAFETY	OCCUPATIONAL HEALTH AND SAFETY<br>PUBLIC HEALTH AND SAFETY<br>POPULATION STUDIES
PSYCHOLOGY<br>PUBLIC POLICY	PSYCHOLOGY<br>HOUSING AND URBAN PLANNING<br>PUBLIC ADMINISTRATION
SOCIOLOGY	SOCIOLOGY
SPORTS AND RECREATION	SPORTS AND GAMES<br>LEISURE AND RECREATION
STATISTICS	STATISTICS
